# La Maison Bleue: Strengthening resilience among migrant mothers living in Montreal, Canada

**DOI:** 10.1371/journal.pone.0220107

**Published:** 2019-07-25

**Authors:** Thalia Aubé, Sarah Pisanu, Lisa Merry

**Affiliations:** 1 Ingram School of Nursing, McGill University, Montreal, Quebec, Canada; 2 La Maison Bleue, Montreal, Quebec, Canada; 3 Faculty of Nursing, University of Montreal, Montreal, Quebec, Canada; 4 InterActions Centre de recherche et de partage des savoirs, Montreal, Quebec, Canada; 5 SHERPA centre de recherche, Montreal, Quebec, Canada; University of Alaska Anchorage, UNITED STATES

## Abstract

**Introduction:**

La Maison Bleue is a community-based perinatal health and social centre in Montreal that provides services during pregnancy up to age five to families living in vulnerable contexts. The study aimed to describe: 1) the challenges and protective factors that affect the well-being of migrant families receiving care at La Maison Bleue; and 2) how La Maison Bleue strengthens resilience among these families.

**Methods:**

We conducted a focused ethnography. Immigrants, refugees, asylum seekers and undocumented migrants were invited to participate. We collected data from November to December 2017 via semi-structured interviews and participant observation during group activities at La Maison Bleue. Data were thematically analysed.

**Results:**

Twenty-four mothers participated (9 interviewed, 17 observed). Challenges to well-being included family separation, isolation, loss of support, the immigration process, an unfamiliar culture and environment, and language barriers. Key protective factors were women’s intrinsic drive to overcome difficulties, their positive outlook and ability to find meaning in their adversity, their faith, culture and traditions, and supportive relationships, both locally and transnationally. La Maison Bleue strengthened resilience by providing a safe space, offering holistic care that responded to both medical and psychosocial needs, and empowering women to achieve their full potential towards better health for themselves and their families.

**Conclusion:**

Migrant mothers have many strengths and centres like La Maison Bleue can offer a safe space and be an empowering community resource to assist mothers in overcoming the multiple challenges that they face while resettling and raising their young children in a new country.

## Introduction

International migration is defined as the movement of persons who leave their country of origin or residence to establish themselves in another country [1]. Migrants include those who choose to move for education, economic or family reasons and also those who are forced to leave their country due to persecution, environmental disasters, war or civil unrest. In Canada, migrants with permanent residency status, including those who initially arrived as refugees, represented 21% of the population in 2011 with projected trends to increase up to 30% in the year 2036 [2]. In the province of Quebec, permanent migrants currently make up 18% of the population with 74% resettled in Montreal, the second largest urban centre in Canada [3]. Montreal is also a major receiving city for asylum-seekers (i.e., those who arrive and make a refugee claim at the border or once they are within the country) and undocumented migrants (i.e., those who enter and/or stay in a country without legal authorization) [1, 4]. A number of migrants move with their families, including their partners and children [5]. Many are also of reproductive age; in Montreal, births among migrants account for more than 50% of the total number of births [6].

Immigration and settlement is a complex process that can lead to economic uncertainty, poor living conditions, social isolation and loss of traditional forms of support from extended family and friends [7, 8]. Many migrants, especially recently-arrived (< 5 years) and those with more vulnerable or precarious (i.e., those in unstable situations or those without authorization to remain in the country) statuses, also face barriers in obtaining employment and accessing social and healthcare services. For migrant families, the stresses of raising children in a new environment may be amplified in the face of these linguistic, cultural, social and financial struggles [8]. Parenting challenges include disciplining and socializing children as they adjust to a new cultural context [9]. Socio-cultural differences may also give rise to tensions on issues related to nutrition, education and daycare [10]. Lack of good quality employment can contribute to inadequate financial resources towards raising and caring for their children [11]. These circumstances may have negative effects on the well-being of the whole family [11–13]. For very young children (0–5 years), during this sensitive stage, stresses may have long-term implications including mental health issues and delays in language and social development [7, 8, 14].

Despite these major stressors, many migrant families show resilience [15–19]. Resilience is a concept that has been defined as a dynamic process in which psychological, social and environmental factors help an individual or family regain, maintain or develop their well-being despite adversities [20–23]. Well-being may include feeling happy, safe and comfortable, having a sense of life satisfaction, and being physically well. Resilience is also demonstrated by adaptive behaviours and better than expected functioning. Protective factors may be internal or external. Internal protective factors may be intrinsic or developed by an individual, such as autonomy, self-esteem, positivity and sociability. External protective factors are elements that exist in an individual or family’s environment, such as supportive relationships with extended family members and friends and community resources.

There is a growing interest in understanding how protective factors operate towards making individuals and families resilient so that policies and practices may be implemented to sustain and promote resilience. Walsh (2003) developed a resilience framework which highlights key family processes that can reduce stresses, foster coping and recovery from life challenges, and empower families towards healing and growth [21]. The framework delineates three domains of key processes, including “belief systems” (how one makes sense of and approaches life challenges), “organizational patterns” (the ability to adjust and adapt, and to tap into social, economic and other resources to deal with challenges) and “communication/problem-solving”(sharing information, expressing emotions and “working-through” the challenges) which can be used as a map to guide the work of health and social care providers towards supporting families to become more resilient. The central tenet of the framework is that all families have strengths and have the potential to overcome and grow from adversity. The framework recognizes that challenges, functioning and well-being vary and that families also evolve over time and therefore may require different approaches depending on a family’s needs and circumstances.

Little research has examined how health centres based within the community promote resilience and support migrant families in adapting and navigating their new environments to raise their young children in a healthy way. La Maison Bleue is a perinatal health and social centre in Montreal that provides medical, educational and social services to families living in vulnerable contexts, from pregnancy up to age five [24, 25]. A significant portion of the families that they offer support and services to, are migrant families. They have three locations, each with an interdisciplinary team of health and social care professionals that provide pre-and post-natal care, psychosocial assessments, mental health and social support, and early childhood development and parenting group programs. These services are provided by a multidisciplinary team comprised of family physicians, nurses, midwives, social workers, specialized educators and psycho-educators. Their model of intervention is rooted in health promotion wherein the intention is not only prevention and treatment but also to support and enable women and their families to achieve their best state of health and well-being [26]. Fundamental to this model is providing a supportive environment, empowering women and families to develop their personal skills towards having more control and capacity to improve their health, and addressing and advocating for healthier social, living, and working conditions for young families [26, 27].

La Maison Bleue is a unique initiative in that it is a community-based non-profit organization that is also part of the publicly funded healthcare system. This private-public structure allows the organization to have some control and discretion over their functioning while simultaneously offering health and social services that are covered under the provincial health insurance scheme [25]. An evaluation was recently conducted to assess the implementation, effects, and economic value of La Maison Bleue’s approach and services and the results were very positive [28]. The report highlighted that La Maison Bleue has been successful in delivering integrated health and social services, providing continuity of care, and improving access to other services and community resources to a number of families living in vulnerable contexts. Based on interviews with women who received services at La Maison Bleue, the report also showed that the organization has increased women’s awareness of services, enhanced women’s sense of parental competence, and fostered the development of support networks for young families. Building on this report, the current study was developed based on an interest by the organization to explore and document more in-depth the perspectives of migrant families specifically, on how La Maison Bleue provides support and promotes well-being for these families. The intent was also to add to the body of literature on resilience, to further inform the approaches, practices, and development of similar community-based health centres working with young migrant families. Using the resilience framework developed by Walsh (2003) as a guide [21], our study aimed to address the following questions: 1) what are the challenges and protective factors that affect the well-being of young migrant families receiving services at La Maison Bleue? and 2) How does La Maison Bleue strengthen resilience among these families?

## Methods

This study was developed in partnership between McGill University and La Maison Bleue. The first author, TA, a nursing student at the time, conducted the project as part of her Masters’ degree and was responsible for developing the protocol, recruiting participants, and collecting and analysing the data. LM was the supervisor and provided guidance on all aspects in the development and carrying-out of the research. La Maison Bleue provided input regarding how best to approach clients and gather data and also facilitated these processes. They also contributed by giving feedback on the interpretation of the findings.

We designed this study using the principles of a focused ethnography, a qualitative methodology that involves describing and interpreting the shared and learned patterns of behaviours, beliefs and values among a specific group in a certain situation, in this case “young migrant families” [29]. Focused ethnographies are also defined by their short and intensive data collection and their focus on a particular social phenomenon [30]. Our focus was specifically on resilience and this concept guided all aspects of the research process, including the interviews, observations and analyses. This methodology was also selected as it has been deemed to be useful for informing the development of health services.

To expand on the earlier evaluation study where women from only one site were interviewed, we recruited and gathered data at two of La Maison Bleue’s locations. These two sites both have a high percentage (18% and 41%) of clients who are recently-arrived migrants and/or who have a precarious migration status. The third location was excluded because it was still in its implementation phase at the time of our study; it had been open for about six months and had only followed a few families, and only for a short period of time at that point. The study was conducted between November and December 2017; recruitment and data collection were limited to this timeframe given that the study was conducted within the context of a Master’s project. Data collection approaches included interviews, participant observation of families during their participation in La Maison Bleue organized group activities, and field-notes. The study was approved by the McGill University Institutional Review Board and the Research Review Office of the University Integrated Health and Social Services Center of West-Central Montreal.

### Recruitment and data collection

Mothers, fathers and extended family members who were migrants (i.e., immigrants, refugees, asylum seekers or undocumented migrants), and who were 18 years or older and could communicate in either English or French (the official language in the province of Quebec), were eligible for participation. We sought to recruit more recently-arrived (< 5 years) migrants as well as those who had been in Canada a little longer. Prior to conducting the study, TA spent two months doing clinical training at La Maison Bleue which provided her an opportunity to familiarize herself with its multidisciplinary teams, the families, and their services. During the recruitment period TA visited La Maison Bleue weekly based on times convenient to TA and staff, and when there were organized activities. La Maison Bleue staff facilitated recruitment for interviews by informing clients that a study was being conducted and introducing TA to them. None of the potential participants approached were individuals that TA had interacted with clinically. La Maison Bleue staff were also not informed of which clients agreed or not to participate in order to maintain confidentiality. For participant observation, groups were selected by the staff based on which groups they felt would be most beneficial to observe. For both the interviews and participant observation, TA was responsible for explaining the study, confirming eligibility and obtaining written consent (consent forms were available in English and French). For participant-observation, group attendees were provided the option to not sign the consent form and be observed -they were told that none of their interactions or quotes would be documented if they chose to not participate. It was also offered to not observe the group session at all if there were any group members who did not want TA to be present. For interviews, one person declined participation and another did not show for the interview at the scheduled time (reason unknown); for observation of the group activities, all attendees agreed to be observed and signed the consent form.

We interviewed a total of nine mothers. Participant characteristics are reported in Table 1. Three women were citizens, one was a refugee, two were asylum seekers and one had no status; three of the women arrived less than two years ago. For the women who had citizenship status, none had a refugee or asylum history. Women were from a range of countries including Morocco, Democratic Republic of Congo and Cameroon. For five women, their primary source of income was their husband’s employment; three were receiving social assistance. The time women were clients at La Maison Bleue ranged from 1.5 months to 9 years.

**Table 1:** Interview participant characteristics.

Variable	Participants (n = 9)
*Age*	
Mean, (SD)	31 years, (4)
Range	26–37 years
*Marital status*	
Married	8
Single	1
*Household Annual Income (Canadian dollars)*	
< $10,000	2
$10,000- $19,999	2
$20,000 - $49,999	2
≥ $50,000	1
Don’t know	2
*Education Level*	
High School	5
College or University	4
*Number of Children*	
Mean, (SD)	2 (1.4)
Range	0–4*
*Age of Children*^†^	
Pregnant	2
Infant	3
Toddler (1–3 years)	6
Pre-school (4–5 years)	4
School age (6–12 years)	5
Adolescent (13–18 years)	1
Unknown (not in Canada)	2
Range	0–15 years*
*Countries of Origin*	Bangladesh, Saint-Lucia, Democratic Republic of Congo^‡^, Morocco, Cameroon, Eritrea, Pakistan^‡^
*Current Immigration Status*	
Citizen	3
Immigrant	2
Asylum seeker	2
Refugee	1
No Status	1
*Time in Canada*	
0–4 years	4
5–7 years	1
8–11 years	4
Mean, (SD)	5.6 years (4.2)
Range	2 months- 11 years
*Health Insurance*	
Provincial health insurance	6
Interim Federal Health Program^§^	2
No coverage/Pays Out-of-Pocket	1
*Length of Time as Client of La Maison Bleue*	
Mean, (SD)	3.4 years (2.8)
Median	2.4 years
Range	1.5 months to 9 years
*La Maison Bleue Services Used*^║^	
Prenatal Care	9
Physical Health Consults	8
Evaluation of Child Development and Childcare Simulation	8
Postnatal Care	6
Vaccinations	6
Psychosocial Services	4
Rights Defense (Immigration, Housing, Education)	2

* 0 is defined as currently pregnant at the time of the interview

^†^ Women can be counted in more than one category

^‡^ 2 participants were from this country

^§^ The Interim Federal Health Program provides limited temporary healthcare coverage for asylum seekers, refugees and other protected persons; refugees however also have coverage under the provincial health insurance scheme

^║^ Included current services and those used in the past

Four interviews were conducted in French (the women from the Democratic Republic of Congo, Morocco and Cameroon), and five were conducted in English (the women from Eritrea, Bangladesh, Pakistan and Saint-Lucia). Interviews were conducted on site in a private office space at a time convenient for the participants. Interviews were 30 to 45 minutes and all but one was audio-recorded; the latter was recorded with hand-written notes. General topics explored included their migration journey, challenges experienced since arriving in Canada, especially in relation to raising and caring for children in a new country, and the resources and coping strategies that have enabled them to maintain the health and well-being of their family. We also asked questions about their experiences and perspectives on services offered La Maison Bleue, particularly regarding what they have found helpful (see S1 and S2 Files for the English and French versions of the interview guide). Participants were encouraged to speak freely and to tell their stories in their own words. Participants completed a sociodemographic form at the end of the interview (see S3 and S4 Files for the English and French versions of the Socio-demographic questionnaire).

We observed 15 migrant mothers during their participation in four group activities (two per site), equalling 8 hours and 45 minutes of observation time. Two were educator groups; one facilitated by a psycho-educator (n = 3), and the other by a specialized educator (n = 6). These groups provide early stimulation for children, strengthen parenting skills and promote the development of a secure attachment between parents and children through games; they also offer an opportunity for parents to learn about the development and age-appropriate behaviours of their children. The other two group activities observed were a pre-natal (n = 5) and post-natal group (n = 1). These groups were facilitated by midwives. In these groups, expectant and new mothers are encouraged to share their knowledge and experiences; information is also provided on various topics such as the stages of labour and breastfeeding. TA did not participate directly in the group activities and recorded all observations with hand-written notes. Observations focused on the setting, the participants (e.g., who was present and the number of group participants), and the reactions and interactions between the participants and the group facilitators with specific attention to discussions and interactions on resources and strengths of families (see S5 File for the observation guide). To limit the disruption of the group activities, we only asked a couple of demographic questions to these participants (country of origin and whether they have been in Canada less than, or more than 10 years). Migrant women observed were from Mexico, Sri Lanka, Bangladesh, India and Algeria and all had been in Canada for less than 10 years.

Additional observations and reflections were noted via field notes throughout the recruitment and data collection period. Notes consisted of general descriptions and impressions of the settings, including the physical environment, and the behaviours and interactions of the staff and families.

### Analysis

All interviews were transcribed verbatim by TA; transcripts, group observation notes and field notes were then compiled for analysis. The analysis was conducted iteratively throughout the data collection process. The process was led by TA who was responsible for coding all of the data, creating the codebook, identifying key themes and extracting relevant quotes. LM supported the analysis process; she listened to all audio-recordings and read the transcripts and independently coded a selection of three interviews. Themes were determined and finalised via back-and-forth discussions between TA and LM. The analysis included an inductive as well as a deductive approach using the resilience framework by Walsh (2003) as a guide [21]. The first step of analysis involved reading the transcripts and notes in order to gain a feel for the data and to document initial impressions. The data were then analyzed using open-coding, which involved a line by line analysis where codes that captured the essence of the data that were related to the research questions, were applied to sections of the text. The three domains of key processes, including “belief systems”, “organizational patterns” and “communication/problem-solving” specified by Walsh (2003) were then used to assign codes a priori. Data were also examined for patterns according to the particularities of certain participant characteristics (migration status, length of time in country). The coding framework was created iteratively to facilitate the coding process. Once codes were developed they were then grouped into categories [29]. Themes were generated by aggregating and clustering categories together which involved combining similar categories and reducing the data that shared commonalities [31]. We used Microsoft Word and Microsoft Excel to manage and analyse the data.

A final validation step of the analysis was done by presenting preliminary results to La Maison Bleue and asking for their feedback.

## Results

### Challenges to well-being

All of the mothers expressed migration specific challenges, including isolation, separation from family, the immigration process, financial strain, poor housing, language barriers, culture and environmental shocks such as the cold weather, and the general busyness and ‘running around’ after first arriving in Canada. The difficult isolation that some women experienced was well illustrated by a mother from Cameroon:

*“I didn’t know anyone*, *I didn’t have any friends*, *when I moved here*, *he* [my husband] *was the only the person I knew in Canada*. *It was an environment where you have to stay all alone*, *I was alone with the children*, *not like in Africa*, *where you see your neighbour*, *you walk around*, *go to the store*, *it is open there”*. Similarly, in speaking about her experience shortly after arrival in Canada, another mother from Bangladesh also shared her experience of feeling alone: *“I didn’t have any friends and also didn’t know much people*, *so all the day I stayed at home alone*, *it was really difficult”*.

In addition to feeling alone and isolated, some of the other challenges experienced had impacts on well-being by affecting women’s ability to resettle and integrate into Canadian society. For example, one mother from Saint Lucia explained how the immigration process was complex and how it had negative effects on day-to-day life for her and her daughter: *“Yes*, *I came with one child*. *So at the time*, *when I did the refugee* [application]*… it was okay* … *it is a long process*, *… but at least you’re getting somewhere*, *but when I didn’t get through*, *the challenges came*, *she* [my daughter] *had to stop school*, *… so it was really*, *really frustrating… they take away all the benefits*, *so you have to start all over again*, *and*, *it is a challenge with a kid*, *for sure*.*”* Another mother from Morocco described her challenges of finding a place to live and some of the discrimination she faced in this process: *“I am looking for another apartment*, *but the rent is high… The problem is that they* [landlords] *don’t accept children… the problem with* [subsidized apartments], *here in this area*, *the problem is*, *yes*, *you will end up in* [disadvantaged] *areas*. *Here*, *these areas*, [there are] *many immigrants*, *it remains like that*, *they don’t speak English or French*, *they have studied abroad*, *they have diplomas from there*, *because*, *they have not found work*, *they become*, *a class* [that is more] *vulnerable*.*”* And a third mother from Pakistan, highlighted weather and language as her main issues to overcome: “*Oh my god*, *in my country*, *there is not a lot of snow*, *but here*, *the weather was hard for us… the second thing was the language*.*”*

Other challenges related specifically to having a baby and parenthood in the context of resettlement. Approximately half of the participants gave birth to their first child shortly after their arrival in Canada. For many of the mothers, raising their children without the support from extended family members further compounded resettlement difficulties. One mother from Bangladesh expressed: *“After I come from the hospital*, *I have to do all the work at home*, *didn’t get much help*, *so everything was really difficult at that time because I was young*, *and didn’t know how to manage a baby*, *also I didn’t know about La Maison Bleue”*. A mother from the Democratic Republic of Congo also shared: *“So*, *looking how to find myself in all of this*, *with a child and the pregnancy and the cold*, *it was a real ordeal*, *you have to run to the left for this step*, *run to the right for another thing*, *it was a new universe*, *and it was very difficult for me*, *very difficult”*.

### Navigating challenges with resilience

Despite the many challenges that women faced, they demonstrated resilience. Support from family and friends, locally and back home, faith and maintaining their language and traditions, and individual strengths (agency, positive outlook) were key to women’s capacity to maintain and develop their well-being.

### Finding meaning, the protective function of relationships and maintaining identity

Attaining a better life and future for their children was often the motivation for the families to migrate to Canada. Children therefore offered a sense of purpose and gave the women courage to overcome their settlement difficulties. Two mothers from the Democratic Republic of Congo respectively stated: *“It was really hard*, *but I can still manage*, *I say*, *there are things that give me strength and I say to myself that it is for a good cause*, *and what is this cause*? *It is for my children”* and *“When I look at my daughter*, *I want to be in good health for her*. *If I’m not well who will take care of her for me*?*”*. As described by Walsh (2003), hope and a belief that life would be better in the future allowed women to make sense of their challenges and to find meaning in their experiences [21].

Close relationships with friends and family members, in Canada as well as back home, were also a key source of strength and support for women. For those who had extended family and friends in Canada the proximity of loved ones provided emotional support as they settled into their new and unknown environments. For some women, it was their close relationships with their husbands that they felt were essential in helping them persevere. As one mother from Pakistan described: *“I did not have any extended family when I arrived here*, *but I was with my husband and children*. *Because of family being here* [her husband and children], *it was not difficult*. *What has helped me settle here is my loving husband”*. For another mother from Morocco who had four children, when asked what helps her the most in raising her family here, she replied, *“the help from my husband”*. Although many husbands worked and were often away from home, the mothers were accepting of their husbands’ absence as they saw how their husbands were working hard to provide for their families. One participant stated: “[My husband is gone] *all day*, *all night*, *but I said it is worth it*, *it will help the family*, *it is very difficult but now it is going well*, *I take care of the house*, *I take care my children”* (Mother from Cameroon). Family cohesiveness and working together towards a common goal helped women to press on [21].

Some of the participants also had other relatives who were already resettled in Canada. In addition to emotional support, their family members, who were well-acquainted with ‘Canadian ways’, knew how systems worked, and who had established networks, were also able to provide informational and instrumental support. When asked about her initial settlement challenges, one participant, who was recently-arrived from Pakistan, said that she had had few because her family was able to assist her: *“Because my father was here for a long time*, *when we have some problem*, *he help[ed] us”*. Another participant from Saint Lucia also emphasized how the presence of extended family was invaluable to helping her adjust and cope with challenges of living in a new country: *“Canada is a good place to be*, *as long as you have assistance*, *that you have people surrounding with love*, *with me*, *I have my two brothers here… so I have two people I can count on*, *without family members though*, *it can be very*, *very challenging”*.

To address their loneliness and to better cope with the difficulties of separation from family and friends, most of the mothers also maintained connections with back home. For one mother, she explained that she kept regular contact with her older sister in Bangladesh who was an important source of social and emotional support for her. She stated: *“Whenever I was depressed*, *my big sister*, *I have a sister*, *she always tell me to try to find happiness* …*”*. For another participant, who had a refugee history and who had a husband and children who had remained in the home country in the Democratic Republic of Congo, communicating with family was vital to her well-being: “*communication with my children…it is the only thing that makes me feel close to them…I listen to them*, *even if I do not see them*, *it makes me happy”*. Almost all of the participants spoke to their loved ones on a daily basis, whether it be their parents, children or extended relatives, they maintained contact through mobile phone applications such as WhatsApp. A mother from Saint-Lucia stated: “[My mother and I speak] *almost everyday*, *and now with WhatsApp*, *we facetime all the time…so it’s easier*, *and it’s free*, *she is far*, *but she is a good support system”*. In accordance with Walsh (2003), exchanging with family and loved ones, whether they were close or far, about experiences and challenges provided an outlet to express and work through feelings and difficulties, it also provided a sense of connectedness and facilitated access to tangible resources which helped with resettlement [21].

Another benefit of having close relationships with family was the opportunity to preserve their language and culture, especially for their children. Practicing family traditions and their faith maintained their sense of identity and was a source of pride, and as per Walsh (2003), was a means to transcend beyond their negative experiences and to continue to live and look towards the future [21]. A mother from Morocco explained: *“We try to keep the traditions*, *to continue them*, *we speak Arab*, *we want to keep the good Arab for them…it is important for them* [my children] *when they return to Morocco*, *to be able to communicate and understand”*. Another mother, who was from Pakistan, echoed this response, and highlighted how it was important to keep celebrating important events: *“All the things we had in Pakistan*, *we will do here too*, *like a special occasion*, *we do the same thing…like when having babies*, [for] *newborn babies*, [we] *have parties*, *we invite friends and families”*. And when asked if anything from her culture helps her overcome challenges here in Canada, one mother from Bangladesh expressed how it was her deep faith and belief in God in particular, that provided comfort, meaning and confidence to continue to move forward: *“Yes*, *yes of course*, *religion*, *because if you trust in God you know you have that confidence*, *you know*, *to do things better*”.

### Staying positive and gaining independence over time

Motivated by their beliefs, values, and hope for a better future, especially for their children, the women maintained a positive outlook and were driven to succeed in their new country. One mother from Cameroon explained: “*For me what I do to keep healthy*, *is to just smile all the time*, *that is my secret*, *even if you are angry*, *you must remain calm and relax* … *Keep smiling to pass the difficulties…so for me it is my attitude*.*”* Women developed a range of skills to transform their realities that initially posed challenges for them. For instance, having the courage to leave the house, walk around a local park and to talk to other women to develop friendships to overcome their isolation. This also served for women to gain a sense of familiarity with their neighbourhoods and community. One participant commented on how the local parks in the neighbourhood allowed her to meet other mothers and their children: “*My husband would go to work and I would stay home*, *but I also went out too*, *to try to make friends… I was shy in English at first…but comfortable in Pashtu… I would go and say hello to people in Pashtu in the Parks to see if they would understand me* …*so I found friends in the Parks”* (Mother from Pakistan). Another participant from Pakistan had a similar experience, *“On our own*, *we go outside*, *we meet the people*.*…* [the first thing we did] *was to go outside and meet people*, *and when have some problems we ask them… in the park”*. The local environment of the park coupled with their sense of determination fostered relationship building with other women in the community.

At first hardships were related more to the unfamiliarity of their new environment, language barriers and isolation. As the women became more comfortable and felt more acquainted with Montreal they ventured to use public transportation on their own; some also obtained a drivers’ license, a skill that they would not have developed in their countries of origin. Learning to be more proficient in English or French was also an important step for mothers towards gaining independence. This process of adapting and overcoming challenges with time was well described by two of the participants (one mother from Pakistan and another from Morocco), as they reflected back on their earlier experiences: *“I understood English*, *but it was a problem*, *many problems*, *then I went to school*, *I took language classes for 2–3 months*, *I developed myself*, *now its fine”*; and *“I am well adapted*, *it* [my French] *has improved*, *I have my driver’s license*, *I am more autonomous than before* [when] *I had to wait for my husband to help with my everything*, *now I am good”*.

For more recently-arrived mothers they were in the beginning phases of gaining skills and becoming more independent and described how they were making plans to improve life in the future. One participant, who had been living in Canada for less than one year, said that she was planning to get both a job and her license. For her, she saw this as directly contributing to the well-being of her family: “*When I can get a job*, *my son will be better”… “After I get my license*, *it will be easy you know”* (Mother from Bangladesh).

The mothers were also motivated by their new found freedoms in Canada and the opportunities that this presented for them as women. An expectant mother from Eritrea explained how coming to Canada provided her possibilities that her country could not offer: *“What made me leave that country*, *was I couldn’t find what I wanted in the future…I could not continue my education*, *could not work*, *no freedom for me*, *everything was complicated…since I had a chance to come here*, *I prefer to stay here*, *I want to stay here”* Another mother from Pakistan also described how she appreciated the openness and the liberty to practice any religion and to do what you want in Canada: *“My mom always said*, *in Canada*, *here you can do what you want*, *you can follow your religion and they don’t force you to do this*, *you can do everything here*, *this is the best thing”*.

Learning skills and becoming autonomous reduced women’s isolation and contributed to their feelings of happiness and sense of accomplishment. As per Walsh (2003), the mothers’ determination to provide a better life for their children fostered an agency to adapt and overcome challenges and to build their personal resources, such as language skills. Over time they gained more confidence and independence, which further reinforced their capacity to move forward.

### A community bond: The role of La Maison Bleue

La Maison Bleue supported women’s resilience processes, including building relationships, finding meaning, maintaining identity, problem-solving and gaining independence, by creating a safe space, providing holistic care and using an empowerment approach.

### A safe space

La Maison Bleue offered an environment where families felt cared for. The team made efforts to get to know women, their husbands and their children on a personal level. They took the time to learn where families were from, their trajectory, and their struggles, strengths and hopes. Through these relationships, the care-providers also built trust, fostered hope and helped women feel safe and supported, including those who had a precarious status, or none at all and/or who had a traumatic migration experience. One mother from Saint Lucia explained: *“If they don’t have the services*, *they will ask another organization*, *but also*, [when you go] *through them*, *you also get a cheaper price*, *because if you don’t have your papers*, *like me*, *you pay so much money*, *so they are involved and make it less* [expensive].*”* Likewise, another mother said: *“The founder [of La Maison Bleue]*, *she’s a genius*, *doing something for people who do not have papers… for me I think it’s genius… I don’t like the [medical clinic] because I don’t have health insurance*, *but at La Maison Bleue I am comfortable*, *I don’t have to show [documents]*.*”*

Staff members also demonstrated genuine respect for families’ values and culture by encouraging, for instance, the women to use their mother-tongue with their children. Trust and safety were further nurtured by offering time and their availability to families. This sentiment was expressed in the following from a mother from Saint Lucia and also from another mother, from the Democratic Republic of Congo: *“You can always call*, *they are just a phone call away*, *they never turn me down*, *they are always there to assist as you need”*; and *“When I don’t know what to do*, *I call La Maison Bleue and I speak to the social worker*. *La Maison Bleue helps me a lot… a lot*, *a lot*, *a lot*.*”*

Families also found a sense of comfort and belonging in a welcoming home-like space. Muslim women felt at ease to remove their head scarves; women felt comfortable to breastfeed; and in group sessions women would smile, laugh, hug and share their fears and as well as their happy moments. The warmth and closeness that women felt with La Maison Bleue staff were well captured in the following quotes from two women:

*“It is a really simple and nice place*, *the people*, *midwife*, *doctors*, *all the social workers*, *they help you from the bottom of their heart*, *they do their best to make you satisfied*, *it’s a really nice place*, *and I am lucky to be here at Maison Bleue”* (Expectant mother from Eritrea).

*“I see them and consider them like my second family”* (Mother from the Democratic Republic of Congo).

By providing an environment where women could connect, relationships naturally and spontaneously formed between women, and helped to create a sense of community. These bonds provided not only practical support but emotional support as well. Women shared and exchanged on their experiences of giving birth in a new country, both positive and negative. For many of the mothers, meeting other women through group activities and socializing together at La Maison Bleue led to meaningful friendships. This experience was explicitly stated by one mother from the Democratic Republic of Congo who shared: *“I did not know anyone*, *even mothers from my own country*, *but thanks to La Maison Bleue*, *I got to meet them”*. Similarly, another mother from Cameroon also described how La Maison Bleue helped her meet other women and how this was a positive experience: “*I have friends that I have met here*, *at La Maison Bleue*, *we discuss*, *we hang out* …*we visit each other* …*when I had my daughter*, *we celebrated”*.

### Holistic care

A holistic care approach refers to a focus on the whole person by addressing both a person’s physical and psychosocial needs [32]. The team at La Maison Bleue offered much more than attending to only the physical health of mothers and their children. For many of the women, the organization helped in other areas such as assisting with immigration paperwork, finding an apartment, looking for employment or even with more simple matters such as how to use the transportation system. For example, one woman described her experience of being helped with using the subway: *“A staff member brought me and another client down into the metro system and explained how it worked”* (Mother from Pakistan).

La Maison Bleue also played an advocacy role and assisted in defending the rights of women and their families. For one woman, the team liaised with an organization to help her resolve a housing issue. The woman shared her experience: *“I was really scared*, *and in front of my doctor and midwife*, *they right away*, *took me into their arms and gave me assurance and companionship that I was not alone and that it would stop”* (Mother from the Democratic Republic of Congo). La Maison Bleue was also committed to standing up for and protecting the rights of their clients who had an undocumented status in Canada. This included providing health and social services and offering assistance to help stabilize their migration situation.

The support and services provided by La Maison Bleue were delivered by a multidisciplinary team all under one-roof. This facilitated access and the coordination of services. This was expressed by women as per the following quotes:

*“They have everything*, *and they try to find everything for you*, *they always have a solution or try to find a solution”* (Mother from Saint Lucia).

*“If I have a professional challenge*, *I will speak with the social worker and they give me an appointment…when we open the La Maison Bleue package*, *there is everything inside”* (Mother from the Democratic Republic of Congo).

When services were not available at La Maison Bleue, staff members ensured their clients were properly referred to other resources, often at a more cost-effective option. One mother stated: *“If they don’t have it*, *they will give you information*, *where to go*, *or the directions*, *if they don’t have the services*, *they will ask another organization”* (Mother from Bangladesh). La Maison Bleue also aimed to connect women and families to community resources. For example, during participant observation, a staff member organized a group activity at the local library so parents could hear about the activities available for their children.

By giving support for a range of issues that extended beyond traditional healthcare, La Maison Bleue was a direct resource for women and their families. By providing practical information and assistance women felt less alone in facing their adversities and more reassured that problems would be resolved. By connecting women to other resources and by showing them how to do things and find information, they also contributed to women developing their confidence and independence.

### Empowerment approach

Empowerment is a process where people’s capacity to meet their own needs is enhanced and individuals can decide for themselves what actions are best for their health and well-being [32]. La Maison Bleue was a place where women were encouraged and supported to acquire new knowledge, gain confidence and build their skills so that they could be more independent. One woman explained: *“*[*The midwife*] *helped me with the birth stuff*, *where to go*, *what I have to do*, *how to prepare*, *the certificate*, *how to get a passport for the baby*, *and many stuffs like that*, *she gave me information*, *that I know now*, *so I can do it on my own”* (Expectant mother from Eritrea).

The conditions for empowerment were created through the development of supportive relationships over time and through the process of providing information, modeling behaviour, giving positive feedback and encouraging women to find their own solutions. For example, during the educator groups the staff would share their observations of child behaviour to help the mothers advance their knowledge on child development and demonstrate various ways to interact with and stimulate their infants and young children. Women were also encouraged to ask questions and seek information. The team members then responded with guidance that was personalized and adapted to their needs. In one instance a mother (from Morocco) was moving to a new area of the city and wanted to know more about the community and the schools. The midwife provided information and also told her that there was a large Moroccan community in the area, which reassured the mother.

The care-providers also built mothers’ self-esteem by commending them on how they were doing great as mothers. For example, one educator said to a mom as she was pulling out her child’s snack: “*That is a good snack*, *it’s healthy*!*”* and a midwife said to a mother as she was breastfeeding (exclusively): *“Congratulations*!*”*. The care-providers also encouraged mothers to participate in activities and to share and learn from each other. Rather than simply telling them what to do, team members fostered a collaborative dynamic between the mothers. An example of this was observed during one of the pre-natal groups where the midwife asked women who had already given birth to share some advice with others and one participant, a mother of four, gave her recommendation based on her experiences: *“Get your hospital card before you go”—*she went on further to explain that this would avoid having go get the card while in labour.

## Discussion

The study findings highlight how migrant women with young children maintained resilience in the face of multiple challenges and how a health and social centre based within the community, La Maison Bleue, contributed to strengthening this resilience. Resettlement and parenthood are both major life transitions that pose significant challenges. Similar to previous studies, participants reported language barriers, isolation, a loss of support for child-rearing, and cultural and environmental stresses [8–10, 16]. These were especially difficult for mothers who gave birth shortly after their arrival in Canada. Key protective factors were women’s intrinsic drive to overcome their struggles, their positive outlook and ability to find meaning in their adversity, their identity (faith, culture and traditions) and supportive relationships from family and friends, both locally and back home. La Maison Bleue fostered resilience by offering a safe space for women and their families, providing holistic care that responded to their diverse needs and empowering women to achieve their full potential towards better health and well-being for themselves and their families.

### Resilience as a process: Building human and social capital

Recent research emphasizes resilience as a process rather than a static trait, influenced by accumulated life experiences. Resilience is seen as a pathway of learning that leads to personal growth [33]. As individuals encounter adversities they build their capacity to make decisions, create a plan, and move forward with a sense of optimism [16, 33]. For the women in our study who had been in Canada for a while, over time they had become more autonomous and sought out opportunities to learn new life skills such as the ability to speak a new language and drive a car. For those who had not been in Canada for long, women were making plans for the future and describing what they were going to do to attain their goals. All of the women also sought out to make and form new connections and friendships by venturing into the community and also through their participation in activities at La Maison Bleue. For some, developing independence was a gendered experience as they were in part driven by the freedom and rights they had gained since coming to Canada. Moreover, for all women being a mother and caring for their children was a great source of pride and motivation and gave meaning to the women’s experiences. Over time with the development of skills, new friendships and the confidence gained, women felt that they had more control over their lives.

A number of studies have shown that migrant women, including those in more vulnerable and precarious circumstances, build skills, knowledge and social networks over time to overcome challenges and to gain mastery over their situation [12, 16, 18, 34–36]. Both human capital (knowledge and skills), and social capital (social networks), have been identified as key for mitigating the negative effects associated with resettlement challenges and for successful adaptation [37]. As social capital develops, this also further reinforces the development of human and economic capital, as networks can enhance access to information and other resources [38]. Given these effects and the overall positive impact on well-being, providing a milieu that promotes social capital development has been recommended as an important approach for supporting migrants [36, 39].

Resilience is more than developing individual abilities, it is also the capacity of the social environment to provide health enhancing resources [23, 33, 34]. The literature shows that community institutions (schools, churches, organizations) can play a critical role in supporting migrant families [15]. Results from the current study showed that La Maison Bleue played a supportive role for women, and promoted the development of social capital, and hence resilience, by connecting women to one another and by providing a space where women could nurture these relationships. This new social network included women of similar ethnic and also of different ethnic backgrounds, including non-migrants, and thus contributed to both bonding within and bridging communities, two dimensions of social capital found to be essential for migrant populations [40].

Through the creation of relationships directly with women, La Maison Bleue also became a direct resource for women. La Maison Bleue was a safe-space in the healthcare systetm where migrant women felt welcomed and respected, and La Maison Bleue also played an advocacy role, including accepting undocumented women as clients, who would otherwise not be eligible for care. La Maison Bleue was therefore a milieu where there was trust and women felt their struggles were heard and supported. Care-providers provided not only emotional support, but also information and practical assistance, and helped families navigate unfamiliar systems and linked them to other community resources. It was evident in the women’s stories that they felt La Maison Bleue not only responded to their health needs but offered much more (i.e., addressing immigration and resettlement issues) than what is traditionally provided by the healthcare system.

La Maison Bleue also had a direct role in supporting women to develop their human capital through their empowerment approach. By providing a safe-space and advocating on behalf of women, the conditions were created where women had trust and felt they could be themselves, ask for help and actively participate in making their lives, as well as those of their peers at La Maison Bleue, better. Care-providers also contributed to building women’s confidence and their capacity to act, by praising and supporting them in their roles as mothers, by offering guidance and resources and by encouraging them to develop skills and assume their independence. Following women and their families from pregnancy up to age five allowed La Maison Bleue to support the development of “resilience as a process” over time.

### Resilience across borders

Multiple studies have found that family and friends are key sources of resilience as they provide emotional companionship and practical information such as how to navigate unfamiliar systems [9, 18]. Connectedness to their families is also maintained via a shared language and practicing traditions, which have also been shown to enhance dignity, and reduce psychological distress, especially in the face of discrimination and exploitation [15]. In this study, relationships were not only local, transnational ties with family back home were also an important source of social support. There is an emerging body of literature showing that transnational ties may affect health both positively and negatively, including lifestyle behaviours, disease management and psycho-social well-being [41–43]. This research suggests that maintaining social ties across borders may help migrants cope with isolation and provide an alternate space for belonging [41, 44], especially during pregnancy and postpartum [45]. Transnational ties, however may also be associated with stress and sadness [41, 44]. Acknowledging and addressing transnational ties in mental health services has been shown to help migrants deal with social and cultural losses and find meaning in their experiences [46, 47]. During the current study we did not observe or hear about transnational ties, including social support from family back home, in care. The literature also reveals little regarding whether and how, health and social care-providers working with migrant families during pregnancy and the early childhood period, mobilize transnational family support as a resource.

### Limitations

This study has some limitations. Our initial objective was to recruit a mix of mothers, fathers and extended family members. However, it proved difficult to recruit fathers and extended family members since they often did not accompany mothers when they came to La Maison Bleue (fathers usually had to work). For feasibility, we limited our sample to migrants who could speak English or French adequately to be interviewed in one of these languages. The timeframe for data collection was also quite short so we did not interview many women. The breadth of perspectives we were able to gather is therefore limited.

We did not follow women longitudinally so we were unable to capture the challenges and resilience trajectories in real time, however we did include women who were new clients as well as women who had been followed at La Maison Bleue for several years. Lastly, the women had few negative comments regarding La Maison Bleue, making it difficult to identify areas where La Maison Bleue may improve services towards further enhancing resilience. Despite these limitations, the study provides evidence as to how migrant women with young children maintain resilience and how La Maison Bleue promotes these processes.

### Implications and future research

Migrants with a refugee, asylum seeker or undocumented status or those in vulnerable contexts are often deemed as overly needy of assistance. In the current political climate, neediness of migrants is viewed negatively and a drain on scarce resources. Contrary to this discourse, migrant mothers in our study had many strengths and were highly motivated to succeed and integrate into their new country. La Maison Bleue was an important community resource in this process by providing support and promoting women’s independence towards building their capacity to be more self-reliant over the long-term. The results are consistent with Walsh (2003) that suggest that when programs/organizations and care-providers are oriented on strengths and resources and support key processes (“belief systems”, “organizational patterns” and “communication/problem-solving”), family functioning and well-being can be enhanced. The results provide insight on what strengths may be identified including social supports locally and abroad, women’s pride in their identity, and their faith, hope and drive to have a better future for themselves and their children. Based on results care-providers may support the resilience process by providing a safe space where women can celebrate their traditions, language and culture, offering support for the range of psychosocial challenges migrant families face (not only addressing medical needs), and creating the conditions where women feel empowered to learn and grow.

La Maison Bleue provides continuity of care and early intervention at a critical time period (0–5 years). Pregnancy is also a prime opportunity to reach more isolated migrant women who may otherwise not seek health services. The findings highlight how La Maison Bleue’s approach aligns with the Ottawa Charter health promotion model including creating a supportive environment, promoting the development of personal skills, advocating for policies to address inequities, and emphasizing health promotion [26]. Furthermore, how La Maison Bleue promotes resilience reflects the key elements of equity-oriented care for marginalized and disadvantaged populations, including “contextually tailored care” (by adapting services to migrant women’s needs, and addressing a range of social determinants); “trauma and violence-informed care” (by being empathetic, providing a safe space and empowering women who have experienced oppression, violence and/or trauma); and “culturally safe care” (by creating a safe space free from discrimination and racism and where women feel respected) [48]. Regarding the social determinants of health, La Maison Bleue addresses a broad array including housing, employment and immigration concerns, access to health and social services (for those with limited or no health insurance), personal health practices, early childhood development, and social support, which have all been identified by childbearing migrant women in previous research as key areas for intervention [49]. The results therefore align with the earlier report, that concluded that La Maison Bleue’s hybrid community-healthcare model and approach to care are strengths of the organization, with the potential to positively impact a range of health and social outcomes [28].

Regarding future research, there is a dearth of literature on how resilience may be promoted among migrant fathers. How to support extended family members and enhance resilience of the whole family, also remains an unexplored area of inquiry. Future research should also examine how transnational support may be fostered by community and healthcare providers.

## Conclusion

Resilience is a dynamic process of building and learning to develop meaningful lives. Key to the migrant mothers’ resilience, were their motivation and drive to succeed to have a better life for their children, their positive outlook, their ability to transcend their negative experiences, and their social networks, which extend across borders. Community-based health and social centres like La Maison Bleue can offer a safe-space and be an empowering resource to assist these mothers in overcoming the multiple challenges that they face while resettling and raising their young children in a new country.

## Supporting information

S1 File Interview guide, English version. (DOCX)

S2 File Interview guide, French version. (DOCX)

S3 File Socio-demographic questionnaire, English version. (DOCX)

S4 File Socio-demographic questionnaire, French version. (DOCX)

S5 File Participation observation guide. (DOCX)

S6 File Data excerpts. (DOCX)
