# Supplementary material for: La Maison Bleue: Strengthening resilience among migrant mothers living in Montreal, Canada
Source: PLoS One. 2019 Jul 25;14(7):e0220107. doi: 10.1371/journal.pone.0220107 (PMC6657858; doi:10.1371/journal.pone.0220107)
Supplement: S1 File — (DOCX) [file pone.0220107.s001.docx]

**Semi-Structured Interview Guide**


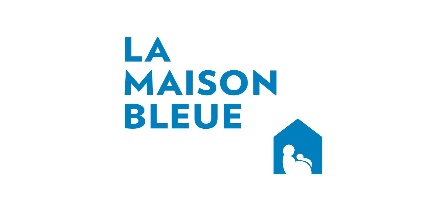
**
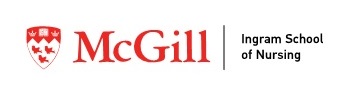
**

Name of interviewer: Date and Time:

**Participant identification number:** ____________

1. Can you tell me a little bit about yourself and when you first came to Canada? Can you tell me about some of the challenges you experienced? Can you tell me about some of the particular difficulties you faced as a parent (or family) with young children?
2. Parlez-moi un peu de vous et lors de votre arrivé au Canada. Quels sont les défis auxquels vous avez fait face?
3. Since you have been here, is there anything that you feel has gotten easier over time? Are there things that you feel have become more difficult?
   - Can you provide examples?
4. Can you share a story about how you and your family managed to overcome some of these challenges that you faced?

- Is there anything about you or your family personally that you feel contributed to your strength to face and overcome challenges?
- Is there anything about your culture or what you took from your experiences in your home country that you feel contributed to your strength to face and overcome challenges?
- Is there anything in your environment or your social surroundings here in Canada that you feel contributed to your strength to face and overcome challenges?

1. In your own words, can you tell me what you think it means to be healthy? What kind of things do you do to keep yourself and your family healthy?

- Is there anything about you or your family personally that you feel contributes to you and your family staying healthy?
- Is there anything about your culture or what you took from your experiences in your home country that you feel contributes to you and your family staying healthy?
- Is there anything in your environment or your social surroundings here in Canada that you feel contributes to you and your family staying healthy?

1. What resources have been helpful for you and your family to stay healthy? And to overcome some of the challenges you and your family have faced since coming to Canada?
   - Can you provide an example?
2. Can you tell me about your experience with La Maison Bleue?
3. What services/programs have you found to be the most helpful? How have they been helpful?
4. What services/programs do you feel have been less helpful? Why do you feel they were less helpful?
5. Do you have any ideas on how the organization can better serve families like yours?
6. Are there any services or resources that you would have liked to have but were not available at La Maison Bleue? If yes, what were they? Is there anything that you feel could be done differently at La Maison Bleue? If yes, can you explain?
7. Is there anything else you would like to say or add?

**This concludes the interview. Thank you again for participating in this research project.**
