# Supplementary material for: La Maison Bleue: Strengthening resilience among migrant mothers living in Montreal, Canada
Source: PLoS One. 2019 Jul 25;14(7):e0220107. doi: 10.1371/journal.pone.0220107 (PMC6657858; doi:10.1371/journal.pone.0220107)
Supplement: S2 File — (DOCX) [file pone.0220107.s002.docx]

**Semi-Structured Interview Guide-French version**


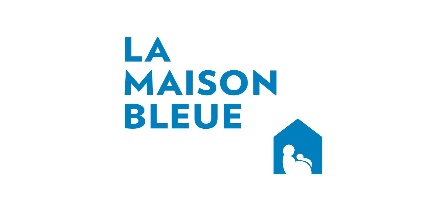
**
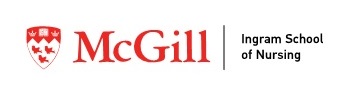
**

Name of interviewer: Date and Time:

**Participant identification number:** ____________

1. Parlez-moi un peu de vous et lors de votre arrivé au Canada. Quels sont les défis auxquels vous avez fait face? Parler-moi des difficultés auxquels vous avez fait face en tant que parent (une famille) avec des jeunes enfants.
2. Depuis que vous êtes arrivés au Canada, est-ce qu’il y a des choses qui sont devenues plus facile au fil du temps? Est-ce qu’il y a des choses qui sont devenues plus difficile au fil du temps?
   - Pouvez-vous donner quelques exemples?
3. Pouvez-vous me parler d’une expérience où vous et votre famille a réussi à surmonter certains de ces défis auxquels vous avez fait face?

- Est-ce qu’il y a quelque chose à propos de vous personnellement ou des caractéristiques familiales que vous croyez ont contribué à votre capacité à surmonter ces défis?
- Est-ce qu’il y a quelque chose à propos de votre culture ou de ce que vous aurez appris de votre pays d’origine que vous croyez a contribué à votre capacité à surmonter ces défis?
- Est-ce qu’il y a quelque chose dans votre environnement ou milieu social ici au Canada que vous croyez a contribué à votre capacité à surmonter ces défis?

1. Dans vos propres mots, que veut dire « être en bonne santé » pour vous? Qu’est-ce que vous faites (vos moyens et stratégies) pour que vous et votre famille restiez en bonne santé?
   - Est-ce qu’il y a quelque chose à propos de vous personnellement ou des caractéristiques familiales que vous croyez ont contribué à votre capacité à demeurer en bonne santé?
   - Est-ce qu’il y a quelque chose à propos de votre culture ou de ce que vous aurez appris de votre pays d’origine que vous croyez a contribué à votre capacité à demeurer en bonne santé?
   - Est-ce qu’il y a quelque chose dans votre environnement ou milieu social ici au Canada que vous croyez a contribué à votre capacité à demeurer en bonne santé?
2. Quelles ressources avez-vous trouvé aidant pour que vous restiez en bonne santé ? Et pour que vous surmontiez les défis auxquels vous avez fait face depuis votre arrivé au Canada ?
   - Pouvez-vous donner quelques exemples?
3. Pouvez-vous me parler de votre expérience par rapport aux services de La Maison Bleue?
4. Quelles services avez-vous trouvez les plus utiles? Comment ont-ils été utiles?
5. Quelles services avez-vous trouvez moins utiles? Pourquoi vous sentez que ces services étaient moins utiles?
6. Avez-vous des idées comment La Maison Bleu pourraient mieux répondre aux besoins des familles migrantes?
7. Est-ce qu’il y a des services ou ressources que vous auriez aimé avoir mais qu’ils n’étaient pas disponibles ou offerts à La Maison Bleue? Si oui, qu’auriez-vous voulu avoir? Est-ce qu’il y a des choses que La Maison Bleue devraient faire différemment? Si oui, pourriez-vous nous en dire un peu plus?
8. Aimeriez-vous nous dire autre chose?

**L’entrevue est maintenant terminer. Merci encore pour avoir participer à ce projet.**
