# Supplementary material for: La Maison Bleue: Strengthening resilience among migrant mothers living in Montreal, Canada
Source: PLoS One. 2019 Jul 25;14(7):e0220107. doi: 10.1371/journal.pone.0220107 (PMC6657858; doi:10.1371/journal.pone.0220107)
Supplement: S3 File — (DOCX) [file pone.0220107.s003.docx]

**Socio-Demographic Form**

**
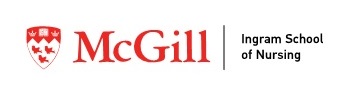
**
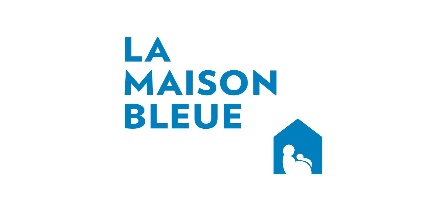


*This information will be collected from study participants at the time of consent for the interview, at which point the right to decline to answer any questions will be explained.*

Participant Identification #: _________________
Participant Family Members________________________________________________

1. **What is your family position in reference to use of services at La Maison Bleue?**

⧠ Mother

⧠ Father

⧠ Extended family member, specify ________________________

**Extended family only need to answer migration questions (Qs. 7-13) and Qs. 21-22.**

**** questions only needed to be completed once per family (either by the Mother or Father).**

1. **How old are you? ___________ (yrs)**
2. **What is your marital status?**
   - Married
   - Consensual union (unmarried partners)
   - Widowed
   - Separated
   - Divorced
   - Single
3. **Who do you live with? Check all that apply. ****
   - Husband/Wife
   - A female or male partner (for unmarried partners)
   - Your Mother/Father
   - Your Brothers/Sisters
   - Your Partner’s Mother/Father
   - Friend(s)
   - I live alone
4. **How many children do you have? How many live with you? ****

**___________ (number of children) ________ (number of children living with you)**

**⧠ currently pregnant**

1. **How many of your children were born in Canada? What year were they born? Which children were followed at La Maison Bleue? ****
2. _________ (year), followed at La Maison Bleue ⧠ Yes ⧠ No
3. _________ (year), followed at La Maison Bleue ⧠ Yes ⧠ No
4. _________ (year), followed at La Maison Bleue ⧠ Yes ⧠ No
5. _________ (year), followed at La Maison Bleue ⧠ Yes ⧠ No
6. _________ (year), followed at La Maison Bleue ⧠ Yes ⧠ No
7. _________ (year), followed at La Maison Bleue ⧠ Yes ⧠ No

**⧠ No children yet born in Canada**

*The next set of questions ask information about your immigration history. We are interested in this information because we want to learn more about the experiences of different migrants to Canada. Any answers you provide will remain confidential, and answering these questions will not affect your immigration application if you are in the process of applying for refugee status, permanent residency, or citizenship.*

1. **In which country were you born?**

**______________________________________**

1. **How long have you lived in Canada?**

**___________ (months) _____________ (years)**

**How long have you lived in Montreal?**

**___________ (months) _____________ (years)**

1. **What is your current immigration status?**

- Immigrant (permanent/landed status)
- Refugee
- Refugee Claimant/Asylum-Seeker
- Temporary worker/Live-in caregiver
- Temporary resident
- Student
- Visitor
- No status
- Undocumented
- Citizen
- Other (please specify): ___________________________

1. **How long have you had this status?**

**___________ (months) ____________ (years)**

1. **Has your immigration status changed since you arrived in Canada?**

- Yes
- No (skip to 13)

1. **If YES, what was your immigration status prior to this status?**

- Immigrant (permanent/landed status)
- Refugee
- Refugee Claimant/Asylum-Seeker
- Temporary worker/Live-in caregiver
- Temporary resident
- Student
- Visitor
- No status
- Undocumented
- Citizen
- Other (please specify): ___________________________
- N/A (did not change status)

1. **Did you ever have refugee status?**

- Yes
- No
- Don’t know

*The next set of questions are more general questions about you.*

1. **What is your highest level of education completed?**

- Primary school
- Secondary diploma
- Postsecondary diploma (e.g trade school, college, university)
- Graduate diploma (Master’s, Doctoral)
- None

1. **What is your current employment status?**

- Working full-time
- Working part-time
- Not working and not looking for work
- Unemployed and looking for work
- Disabled or retired and not looking for work
- Currently in school
- Other (please specify): _________________

1. **If you are working, what is your current job?**

_____________________________________

1. **How do you pay for healthcare (check all that apply)?**

- Government funding (Medicare/RAMQ)
- Refugee insurance (IFHP)
- Private insurance
- Pay out of pocket

1. **What are your sources of income (Check all that apply)? ****

- Employment/Job
- Partner’s employment/job
- Help from relatives
- Social assistance (unemployment, child support, disability)

1. **What is your combined family income for the past 12 months, before taxes from all sources (job, social assistance, help from relatives). If you do not know exactly, please estimate. ****

- Less than $ 9,999
- $ 10, 999 - $ 19,999
- $20,000 - $49,999
- $50,000 - $99,999
- More than $ 100,000
- Don’t know
- Chose not to answer

1. **What is your mother tongue/first language?**

**_______________________________________**

1. **How well do you know French?**

|  | Fluent | Well | With Difficulty | Not at all |
| --- | --- | --- | --- | --- |
| **Speak** |  |  |  |  |
| **Read** |  |  |  |  |
| **Write** |  |  |  |  |
| **Understand** |  |  |  |  |

1. **How well do you know English?**

|  | Fluent | Well | With Difficulty | Not at all |
| --- | --- | --- | --- | --- |
| **Speak** |  |  |  |  |
| **Read** |  |  |  |  |
| **Write** |  |  |  |  |
| **Understand** |  |  |  |  |

*These next questions ask more detailed information about the services you receive from La Maison Bleue. These are the last set of questions before the interview.*

1. **For how long has your family been receiving services from La Maison Bleue? ****

**_________ (months) ___________________ (years)**

1. **From which La Maison Bleue location does your family currently receive services? Check all that apply. ****

- Parc-Extension
- Côte-des-Neiges
- Saint-Michel

1. **Which services or programs does your family receive? Check all that apply. ****

|  | **Currently** | **In the Past** |
| --- | --- | --- |
| Prenatal care |  |  |
| Postnatal Care |  |  |
| Physical health consultations |  |  |
| Immunizations |  |  |
| Psychosocial assistance |  |  |
| Psychotherapeutic assistance |  |  |
| Advocacy services (help with immigration, schools, housing) |  |  |
| Early child care stimulation |  |  |
| Evaluation of child development |  |  |

1. **Which group sessions do you and/or your family members partake in at La Maison Bleue? Check all that apply. ****

|  | **Currently** | **In the Past** |
| --- | --- | --- |
| Family Health Meetings |  |  |
| Prenatal Classes (midwife) |  |  |
| The Art of Parenting |  |  |
| Infant Massage Group |  |  |
| None |  |  |

1. **Which of your family members receive La Maison Bleue services? Check all that apply. ****
   - Myself
   - Husband/Wife
   - A female or male partner (for unmarried partners)
   - Children
