# Supplementary material for: La Maison Bleue: Strengthening resilience among migrant mothers living in Montreal, Canada
Source: PLoS One. 2019 Jul 25;14(7):e0220107. doi: 10.1371/journal.pone.0220107 (PMC6657858; doi:10.1371/journal.pone.0220107)
Supplement: S4 File — (DOCX) [file pone.0220107.s004.docx]

**Formulaire sociodémographique**

**
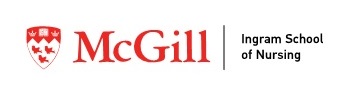
**
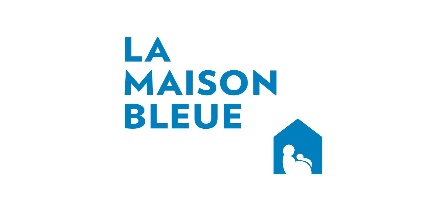


*Cette information sera recueillie au moment du consentement pour l'entrevue. Nous rappellerons aux participants qu’ils ont le droit de refuser de répondre à n’importe quelle question qu’ils ne souhaitent pas y répondre.*

Numéro d’identification du participant: _________________
Membres de la famille participants à l’étude :_______________________________________

1. **Quel est votre rôle dans la famille en référence aux services de La Maison Bleue ?**

⧠ Mère

⧠ Père

⧠ Membre de la famille étendue, précisez ________________________

**Les membres de la famille étendue doivent seulement répondre aux questions de migration (Qs 7-13) et Qs 21-22.**

**Les questions** doivent être complétées seulement une fois par famille (par la mère ou le père)**

1. **Quel âge avez-vous? __________(années)**
2. **Quel est votre état civil?**
   - Marié(e)
   - Conjoint(e) de fait
   - Veuf/veuve
   - Séparé(e)
   - Divorcé(e)
   - Célibataire
3. **Avec qui habitez-vous? (Cochez toutes les réponses qui s’appliquent.) ****

- Femme/mari
  - Conjoint/conjointe
  - Votre mère ou père
  - Vos frère(s) ou sœur(s)
  - La mère ou père de votre mari/femme (conjoint/conjointe)
  - Ami(es)
  - Je vis seul(e)

1. **Combien d’enfants avez-vous? Combien d’enfants vivent avec vous? ****

**__________ (nombre d’enfants) ________ (nombre d’enfants qui vivent avec vous)**

**⧠ Enceinte**

1. **Combien de vos enfants sont nés au Canada? En quelle année sont-ils nés?**

**Quels enfants ont été suivis par La Maison Bleue? ****

1. _________ (année), suivi par La Maison Bleue ⧠ Oui ⧠ Non
2. _________ (année), suivi par La Maison Bleue ⧠ Oui ⧠ Non
3. _________ (année), suivi par La Maison Bleue ⧠ Oui ⧠ Non
4. _________ (année), suivi par La Maison Bleue ⧠ Oui ⧠ Non
5. _________ (année), suivi par La Maison Bleue ⧠ Oui ⧠ Non
6. _________ (année), suivi par La Maison Bleue ⧠ Oui ⧠ Non

**⧠ Aucun enfant né au Canada**

*Les prochaines questions portent sur votre expérience d’immigration. Nous demandons ces questions parce que nous voulons en apprendre davantage sur les différentes expériences vécues par les migrants qui arrivent au Canada. Toute information fournie restera confidentielle, et votre participation à cette étude n'aura aucun impact sur votre demande d'immigration si vous avez une demande pour le statut de réfugié, la résidence permanente ou la citoyenneté en cours.*

1. **Quel est votre pays de naissance?**

**______________________________________**

1. **Depuis combien de temps avez-vous vécu au Canada?**

**___________ (mois) _____________ (années)**

**Depuis combien de temps avez-vous vécu à Montréal?**

**___________ (mois) _____________ (années)**

1. **Quel est votre statut d’immigration actuel?**

- Immigrant(e) (résident(e) permanent(e))
- Réfugié(e)
- Demandeur(e) de statut de réfugié(e)/demandeur(e) d’asile
- Travailleur(e) temporaire/Aides familiaux résidents
- Résident temporaire
- Étudiant(e)
- Visiteur
- Aucun statut
- Sans papiers
- Citoyen(ne) Canadien(ne)
- Autre (précisez): ___________________________

1. **Depuis combien de temps avez-vous ce statut?**

**___________ (mois) ____________ (années)**

1. **Avez-vous changé de statut d’immigration depuis votre arrivé au Canada?**

- Oui
- Non (Passez à la Q-13)

1. **Si OUI, quel était votre statut auparavant?**

- Immigrant€ (résident(e) permanent(e))
- Réfugié(e)
- Demandeur(e) de statut de réfugié(e) / Demandeur(e) d’asile
- Travailleur(e) temporaire/ aides familiaux résidents
- Résident temporaire
- Étudiant(e)
- Visiteur
- Aucun statut
- Sans papiers
- Citoyen(ne) Canadien(ne)
- Autre, précisez _________________
- Ne s’applique pas (pas changé de statut)

1. **Avez-vous déjà eu le statut de réfugié?**

- Oui
- Non
- Vous ne savez pas/vous ne souvenez pas

*Les prochaines questions sont des questions plus générales à propos de vous.*

1. **Quel est le plus haut niveau d’éducation que vous avez complété?**

- École primaire
- École secondaire
- Diplôme post-secondaire (diplôme d’études professionnelles, cégep, diplôme de premier cycle)
- Diplôme de deuxième ou troisième cycle
- Aucun

1. **Quel est votre statut actuel de l’emploi?**

- Travail à temps plein
- Travail à temps partiel
- Ne travaillez pas et ne cherchez pas de travail
- Sans emploi et cherchez de travail
- Handicapé/Invalidité ou retraite et ne cherchez pas de travail
- Aux études
- Autre (préciser) : ________________________

1. **Si vous travaillez, quel est votre emploi actuel?**

_____________________________________

1. **Qui paie pour vos soins de santé? (Cochez les réponses qui s’appliquent)**

- Assurance maladie publique
- Assurance gouvernementale pour les réfugiées et les demandeurs d’asile (PFSI)
- Assurance privée
- Vous payez pour vos services de santé

1. **Quel sont vos sources de revenus (Cochez les réponses qui s’appliquent) ****

- Emploi/Travail
- Emploi/Travail du conjoint(e)
- Aide de la famille
- Aide sociale (chômage, pension alimentaire pour enfants, d’assurance invalidité)

1. **Quel est le revenu total de votre maisonnée pour les douze derniers mois, de toutes sources (emploi, aide sociale, aide de la famille) et avant imposition. Si vous ne savez pas exactement, veuillez estimer. ****

- Moins de $ 9,999
- $ 10, 999 - $ 19,999
- $20,000 - $49,999
- $50,000 - $99,999
- Plus que $ 100,000
- Vous ne savez pas
- Ne choisissiez pas de répondre

1. **Quelle est votre langue maternelle?**

**_______________________________________**

1. **Quel est votre niveau de connaissance de la langue française?**

|  | **Couramment** | **Bon** | **Avec difficulté** | **Pas du tout** |
| --- | --- | --- | --- | --- |
| **Parlé** |  |  |  |  |
| **Lu** |  |  |  |  |
| **Écris** |  |  |  |  |
| **Compris** |  |  |  |  |

1. **Quel est votre niveau de connaissance de la langue anglaise?**

|  | **Couramment** | **Bon** | **Avec difficulté** | **Pas du tout** |
| --- | --- | --- | --- | --- |
| **Parlé** |  |  |  |  |
| **Lu** |  |  |  |  |
| **Écris** |  |  |  |  |
| **Compris** |  |  |  |  |

*Les questions suivantes portent sur les services que vous recevez de La Maison Bleue.*

*Ce sont les dernières questions avant de commence l’entrevue.*

1. **Depuis combien de temps votre famille recevez des services de La Maison Bleue? ****

**_________ (mois) ___________________ (années)**

1. **À quel endroit de La Maison Bleue est-ce que vous recevez actuellement des services? (Cochez toutes les réponses qui s’appliquent) ****

- Parc-Extension
- Côte-des-Neiges
- Saint-Michel

1. **Quels services ou programmes de la Maison Bleue est-ce que vous et votre famille utilise ? (Cochez toutes les réponses qui s’appliquent) ****

|  | **Actuellement** | **Auparavant** |
| --- | --- | --- |
| **Soins prénataux** |  |  |
| **Soins postnatals** |  |  |
| **Consultations médicales (santé physique)** |  |  |
| **Vaccinations** |  |  |
| **Services psychosociaux** |  |  |
| **Psychothérapie** |  |  |
| **Défense des droits (Immigration, logement, système d’éducation)** |  |  |
| **Services de développement destinés aux jeunes enfants** |  |  |
| **Services d’évaluation du développement de l’enfant** |  |  |

1. **Dans quelles activités de groupe participez-vous et/ou les membres de votre famille? (Cochez toutes les réponses qui s’appliquent) ****

|  | **Actuellement** | **Auparavant** |
| --- | --- | --- |
| **Réunions santé familiale** |  |  |
| **Cours prénataux (avec sage-femme)** |  |  |
| **« L’Art d’être parent »** |  |  |
| **Groupe de massage pour bébés (avec psychoéducatrice)** |  |  |
| **Aucun** |  |  |

1. **Quels membres de votre famille reçoivent des services de La Maison Bleue? (Cochez toutes les réponses qui s’appliquent) ****
   - Moi-même
   - Femme/mari
   - Conjoint/conjointe
   - Enfants
