# Supplementary material for: La Maison Bleue: Strengthening resilience among migrant mothers living in Montreal, Canada
Source: PLoS One. 2019 Jul 25;14(7):e0220107. doi: 10.1371/journal.pone.0220107 (PMC6657858; doi:10.1371/journal.pone.0220107)
Supplement: S5 File — (DOCX) [file pone.0220107.s005.docx]

**Participant Observation Guide**

**
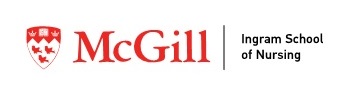
**
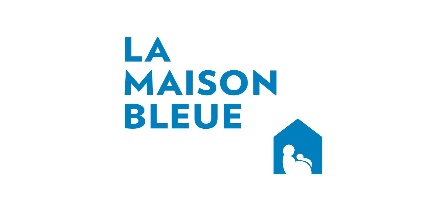


*This information will be collected from study participants at the time of consent for the participant observation at the group sessions.*

Participant ID: ________________________________________________________

1. **Are you a migrant?** (i.e., permanent/landed status, refugee or refugee claimant, temporary resident, visitor, student, or other)/Êtes-vous un migrant (c.à.d. êtes-vous né(e) à l’extérieur du Canada) ?

⧠ Yes / Oui

⧠ No / Non

1. **Did you recently arrive in Canada?** (≤ 10 years in Canada) / Est-ce que vous-êtes arrivés au Canada au cours des dix dernières années ?

⧠ Yes / Oui

⧠ No / Non

1. **What is your family position in reference to use of services at La Maison Bleue?** / Quel est votre rôle dans la famille en référence aux services de La Maison Bleue ?

⧠ Mother / Mère

⧠ Father / Père

⧠ Extended family member/ un membre de la famille étendue, specify/préciser svp:

____________________________________________________

**Participant Observation Guide**

*Fieldnotes will be recorded on the following guiding points. The proposed guidelines are provisional and will be continuously adapted during the course of data collection, aiming to capture the strengths and resiliency factors of recent migrants with young children.*

1. Physical Setting

- What is the group session?
- When is the session taking place? (Date, time, duration, location)
- What are the main features of the setting?
  - What teaching tools or equipment is present and how are they used?
  - Number of facilitators
  - Role of facilitator(s)

1. The Participants

- What are the individual characteristics of participants (age, gender, physical appearance)
- Number of participants present
- Roles of participants

1. Activities and Interactions
   - How are the participants interacting with each other?
     1. Who speaks to whom and for how long
     2. Who initiates the interaction
     3. Language, tone of voice and volume
   - How are the facilitators interacting with the participants?
     1. Who speaks to whom and for how long
     2. Who initiates the interaction
     3. Language, tone of voice and volume
   - What methods of communication are being used?
     1. Non-verbal communication
     2. Gestures
     3. What emotions are being expressed by participants and/or facilitators?
2. Content
   - Which topics are discussed and by whom?
   - What does the group session reveal about recent migrant challenges and strengths?
     1. What are the participants saying about their challenges?
     2. What are the participants saying about their strengths and/or resources that have helped them over come the challenges?
     3. What is being recommended and/or offered by the facilitator(s)?
