# Supplementary material for: La Maison Bleue: Strengthening resilience among migrant mothers living in Montreal, Canada
Source: PLoS One. 2019 Jul 25;14(7):e0220107. doi: 10.1371/journal.pone.0220107 (PMC6657858; doi:10.1371/journal.pone.0220107)
Supplement: S6 File — (DOCX) [file pone.0220107.s006.docx]

**Summary**

| **Themes** | **Categories** | **Details** |
| --- | --- | --- |
|  |  |  |
| **MIGRANT FAMILY CHALLENGES** | **Immigration Process** | Applying for refugee status, not having status, access to services (daycare, healthcare, study), education recognition, discrimination, motivations to leave home country |
|  | **Family Separation** | Worry about family who remained behind |
|  | **Finances** | Paying for Bills, Finding Lodging they can afford, low-income |
|  | **Busy-ness** | Recent arrivals, lots of time to get settled, navigating new environments, less time for leisure or spending time together |
|  | **Living in Underprivileged Areas** | Living in underprivledged neighbourhoods, housing |
|  | **Culture and Environmental Shocks** | Weather Challenges, Cold, Food Differences |
|  | **Language Barriers** | Inability to communicate, participate, employment |
|  | **Isolation** | Loss of support, no sense of community, single-parents |
|  | **Child Development Issues** | Concerns over behaviour, delays (autism) |
|  | **Pregnancy & Parenting Stresses** | Fatigue, pre-eclampsia, prematurity, managing multiple responsibilties, |
|  |  |  |
|  |  |  |
| **RESILIENCY** |  |  |
|  | **Positive Attitude** | Smiling, being Happy, respect the world of others, live and let live approach, focusing on the positive, anf solutions, not getting caught in the negative |
|  | **Support and Love** | Especially, positive relationship with Husband, family support (brothers, sisters, parents), time together |
|  | **Children as Purpose** | Joy, and source of courage |
|  | **Agency** | Determined and Independent, looks for services, training and activities, learning to drive, going to school, learning languages, wanting to work, going out of the house, doing stuff on their own, takes time for self |
|  | **Friends** | Especially other women/mothers who have same background/language, practical, social and informational support |
|  | **The Park** | Environment, almost all women said they met friends in the park, going outside |
|  | **Family Values** | Brought up, family values to be strong, sense of family, healthy behaviours, eating good food |
|  | **Transnational Connections** | Almost all mentioned maintaining contact via WhatsAPP, family back home support, connecting to kids/husband back home is ressuring, business back home as source of revenue |
|  | **Appreciative of Freedom** | Promotes agency and positive attitude |
|  | **Religion** | Prayer, Church |
|  | **Maintaining Traditions and Language** | Use of mother-tongue within family, maintain identity |
|  | **Having Help and Support** | Assistance from government and organizations (CLSC, social assistance) |
|  |  |  |
|  |  |  |
| **LMB FOSTERING RESILIENCY** |  |  |
| 1. Building Safe Space | **Feels Like Home** | Safe Space, Trust with Staff, Proper Equipment |
|  | **Know their Clients** | Building Relationships, Getting to Know, Culturally Attuned |
|  | **Always There** | Giving Time, Never turn me away, answering questions, providing information |
|  |  |  |
|  |  |  |
|  |  |  |
| 2. Holistic Care | **Varied Team** | Nurse, MD, SW, Educator (interdisciplinary team) , dedicated, motivated staff members |
|  | **Helps with All** | How to use metro, gives clothes, coupons, teaching, reference letters, immigration, preparation for birth, access to school, apartment issues, sharing information |
|  | **All Services in One Place** | Comprehensive and faster than other services (availability an issue) |
|  | **Protection of Rights** | No status, stand up against discrimination, right to practice own culture |
|  |  |  |
|  |  |  |
| 3. Empowerment | **Gives Hope** | Builds Confidence, Sense of Self, Normalizes |
|  | **Connects Women** | Creates social contacts between women, meet friends through LMB, children meet children, sharing stories, similar experiences, peer to peer learning |
|  | **Models Behaviour/Active Participation** | Rather than telling mothers what to do , encourage involvement, collaborative approach, demonstrating |
|  | **Navigation of System** | Tell me where to go, how to get stuff, other services, referrals |

**Interviews**

| **Categories** | **Codes** | **Excerpt** | **Source** |
| --- | --- | --- | --- |
| **Agency** | **Autonomy** | and then we can start part-time | Interview #1 |
| **Agency** | **Autonomy** | 01: Yes, yes, after I get my license, it will be easy you know, with a car, go. | Interview #1 |
| **Agency** | **Autonomy** | Now, I can go outside on my own | Interview #1 |
| **Agency** | **Gaining independence to leave house from time and experience** | TA: You know the area more.. | Interview #1 |
| **Agency** | **Obtaining a driver’s license to assist with employment seeking** | TA: Yes, can travel around, | Interview #1 |
| **Agency** | **Speaking a dominant language, facilitating social interactions** | 01: Ya, at that time, I couldn’t talk, now I can talk with people, | Interview #1 |
| **Agency** | **Speaking a dominant language, facilitating social interactions** | 01: Yes, yes…well when I was new, I was pregnant my first time, I couldn’t tell to the doctor that there was a problem , but now, like whenever I come, *** asked me, or the doctor asks me, how is me, how is your mood? Is everything okay or not? If there is anything I can tell them, | Interview #1 |
| **Agency** | **Strategies to build social relationships** | TA: And you met some,,, because in this area, there are more Bangladesh families | Interview #1 |
| **Agency** | **Willingess to Learn – Building Capital** | so we start driving class, a couple months ago, now I am doing that one | Interview #1 |
| **All Services in One Place** | **LMB external referral for child** | and I start coming to the group class,  and then they also send papers to CLSC you know, the CLSC the special group class also , | Interview #1 |
| **Always There** | **LMB taking time for their clients to solve problems** | : Yes, because whenever I need help, they help me, they give me time. | Interview #1 |
| **Always There** | **Seeking-out MB personnel with question about child** | that’s why I talk with *** now about what should we do,  and | Interview #1 |
| **Child Development Issues** | **Behavioral concerns with early childcare development, not understanding signs of danger, falling, eating non-edible items** | I also was thinking, you know he doesn’t go to much, and it was really hard, and he doesn’t understand any danger, and like all the time would put nasty things in his mouth, like chalk, would fall down, would do little things | Interview #1 |
| **Child Development Issues** | **Child development diagnosis, coping** | they say it is Autism, and he is four, but developed to only one or 1.5 year, ohh.. then, like we were heartbroken , | Interview #1 |
| **Child Development Issues** | **Parenting Concerns, Not eating at school,** | He doesn’t eat anything? At school? 01: No drink, no eat, | Interview #1 |
| **Child Development Issues** | **Reduced childhood socialization** | but I tried to come with my husband that we should put him to the daycare, and he will be okay, but he doesn’t like this --- | Interview #1 |
| **Children as Purpose** | **Children as Reason** | if we can help, he will be okay. | Interview #1 |
| **Children as Purpose** | **Children as Reason** | when I can get a job, my son will be better, | Interview #1 |
| **Family Seperation** | **Seperation of Family** | but my family from Bangladesh  and | Interview #1 |
| **Family Seperation** | **Seperation of Family** | everybody cannot come everyday to help you, you know with these things. But this time, maybe my Mother will try… | Interview #1 |
| **Feels Like Home** | **Gaining trust to share experiences with LMB** | But now you are more comfortable, you have some trust with the staff. 01: yes, yes | Interview #1 |
| **Feels Like Home** | **Building (+Maintenance of) Trustworthy Relationships/Accompainement/Being Available** | Yes, connections, yes anything I can tell her | Interview #1 |
| **Feels Like Home** | **Building (+Maintenance of) Trustworthy Relationships/Accompainement/Being Available** | I can talk with *** | Interview #1 |
| **Feels Like Home** | **Social Climate (Safe Space, Respect, Warm, Welcoming, Adequate Equipment)** | it is very good. I really love this place | Interview #1 |
| **Finances** | **Financial challenges of growing the family** | oh yes my husband also realized that ** is also getting bigger now, | Interview #1 |
| **Finances** | **Financial Challenges (Raising Family)** | , and if we are to increase our financial situation , | Interview #1 |
| **Finances** | **Financial challenges of growing the family** | and we should buy house for him | Interview #1 |
| **Friends** | **Building Relationships** | 01: In the family, go outside, talk with families, | Interview #1 |
| **Friends** | **Friendship support and guidance on challenges** | 01: Yes, I have one friend, she also is here, she told me that, because a couple months ago, I was so desperate, what should I do, I want to go French class, Driving class, she told me take time, slowly slowly, you will finish drivers class, get license, then you will start French class, part-time or full-time or whatever | Interview #1 |
| **Friends** | **Strategies to build social relationships** | like when the summer come, I start going out to the baby park, meet new people, from my country you know? | Interview #1 |
| **Friends** | **Building Relationships** | Meet people and make friends | Interview #1 |
| **Friends** | **Peer Support, meeting people with children** | , I meet with new people and friends, and they also have kids – | Interview #1 |
| **Having Help and Support** | **Use of CLSC health services** | also didn’t know about La Maison Bleue, I only went to the CLSC for vaccine or something, | Interview #1 |
| **Having Help and Support** | **Positive outcome from referral to CLSC services from LMB** | Ya, ya, but my social worker gets, we get social worker from CLSC for (A) and they fill lots of papers to the government and we get support, | Interview #1 |
| **Having Help and Support** | **Social referral to LMB** | so, then I get pregnant and one of my friend tell me about La Maison Bleue, | Interview #1 |
| **Having Help and Support** | **Access to Social Assistance** | oh yes, also he loses his job, we are on social assistance, | Interview #1 |
| **Having Help and Support** | **Use of government financial support** | all together | Interview #1 |
| **Helps with All** | **Informational Support** | She is the one that, if there is anything I can ask you know? | Interview #1 |
| **Helps with All** | **Collaborative creation of care plan for child (MB and Family Member)** | I, I make a plan with *** how I can help (A),  and I was working all the time, day and night, to support him , | Interview #1 |
| **Helps with All** | **Instrumental Support** | Felix, the group class, and the meeting with (A) | Interview #1 |
| **Helps with All** | **Educator assessment and evaluation of child development** | but when *** looked, like evaluation, like what he should do at his age, then I realized that (A) doesn’t know lotssss *(emphasized)* of things, lots of things,  then | Interview #1 |
| **Isolation** | **Low forms of social support** | I didn’t have any friends and also didn’t know much people, so all the day I stayed at home alone, | Interview #1 |
| **Isolation** | **Isolation** | it was really difficult – like um depression, | Interview #1 |
| **Isolation** | **Isolation** | because not too much friends, | Interview #1 |
| **Isolation** | **Isolation** | , before that my husband doesn’t like me to go outside , | Interview #1 |
| **Isolation** | **Building Relationships** | 01: Ya Ya, because when I was alone then, I start making friends , | Interview #1 |
| **Isolation** | **Isolation** | TA: Ya, because it is lonely? 01: Ya, ya | Interview #1 |
| **Isolation** | **Isolation** | 01: At that time, I was nervous to go outside, I was always dependent on my husband | Interview #1 |
| **Isolation** | **Isolation** | I go different place, its lots of change, because all the time you stay at home, its very depressing. | Interview #1 |
| **Language Barrier** | **Language barrier for finding employment** | and I, because I also see, its really hard without French, to find a good job, because its linked to all jobs,  and | Interview #1 |
| **Language Barrier** | **Strategy to overcome language barrier** | and that’s why I want to start the French classes | Interview #1 |
| **Language Barrier** | **Language Barriers** | also my English problem and thinking | Interview #1 |
| **LMB Outcome** | **Outcome: Gratitude, Deep Appreciation, Referring Others, Wanting to Remain as Client** | and I like Maison Bleue, I want always | Interview #1 |
| **LMB Recommendation** | **MB Recommendation** | 01: Yes, nutritionist. | Interview #1 |
| **Maintaining Traditions and Language** | **Marital priority for mother to stay at home with children** | And, because his ex-wife also had an affair before, also that’s why he didn’t want me to work outside, | Interview #1 |
| **Maintaining Traditions and Language** | **Marital priority for mother to stay at home with children** | , I don’t know why, sometimes he said that because I have to see the children, because I should be with (A) at home, yes its true, I know, It is really hard, I have to give him much time | Interview #1 |
| **Pregnancy & Parenting Stresses** | **Difficulties with first-time parenting** | I have to do all the work at home, didn’t get much help, so then everything was really difficult at that time because I was young, and didn’t know how to manage a baby, | Interview #1 |
| **Pregnancy & Parenting Stresses** | **Managing multiple responsibilities of being a parent** | And um, difficult is like, only thing is this things, I get very you know nervous if I find job, because language and also manage the house, kids, this things, | Interview #1 |
| **Pregnany Stresses** | **Parenting Concerns, dealing with difficulties of being pregnant** | Difficult is like, when dealing with pregnancy, these things are difficult, | Interview #1 |
| **Religion** | **Religion as source of hope to find strength** | Yes, yes of course, religion, because of, if you trust in God you know, you have that confidence, you know, to do things better,   and | Interview #1 |
| **Support and Love** | **Family Time Together** | hen we went to Bangladesh to see our family and we stay like one-month, so after we come back, | Interview #1 |
| **Support and Love** | **Family Time Together** | and spend good time, | Interview #1 |
| **Support and Love** | **Family Time Together** | TA: Okay – so you eat together around the table every night | Interview #1 |
| **Support and Love** | **Mutual understanding and respect between parents** | My family, this family, like um before, my and my husband, lots of understanding problems you know, so now day by day, now, we have now two kids, he understand more me, and because I --- , he also understand me that, I am not kind of woman. (Laughs) | Interview #1 |
| **Support and Love** | **Lack of Partner Support** | also my husband was not able to understand me,  then | Interview #1 |
| **Support and Love** | **Lack of Partner Support** | things my husband didn’t understand, you know? This is okay, it will be okay. So, I didn’t get that mental support. | Interview #1 |
| **Support and Love** | **Lack of Partner Support** | Then I, come home, try tell my husband, he says no its nothing like that, its bullshit. | Interview #1 |
| **Support and Love** | **Peer Support, spending time with friends** | : Yes, and friends house, for tea and coffee | Interview #1 |
| **TIME - Improvements in Child Interventions** | **TIME – Improvement overtime, over interventions** | , I just want him to be okay.  Then slowly, slowly, with days passing, we like do therapy, we get support from presidents choice and government money, we do therapy, now our son is okay, he is doing well, | Interview #1 |
| **TIME - Improvements in Child Interventions** | **Time- Positive Impact** | there is lots of differences | Interview #1 |
| **TIME - Use of DayCare Servies for 2nd Child** | **Relationship to Code: As time passed and with her experience, not wanting to repeat or create a similar environment for her next child, she seeks out daycare services.** | And you stay at home with your other son? Or is he going to daycare? Ya, Daycare, ya. | Interview #1 |
| **Transnational Connections** | **Family support and guidance on challenges** | also yes, whenever I was depressed, my big sister, I have a sister, she always tell me that try to find happiness by yourself, don’t always depend on your husband, you know? You also can do things better, what will make you happy. | Interview #1 |
| **Transnational Connections** | **Family support and guidance on challenges** | 01: My sister tries to get me to understand, you celebrate, never mind him, you celebrate | Interview #1 |
| **Varied Team** | ***Holistic Approach MB-Continuity of Care** | I also tell her about our son, that my son is still doesn’t understand a lot of things, then Anne said, okay we will make an appointment with Felix , | Interview #1 |

| **Categories** | **Code** | **Excerpt** | **Source** |
| --- | --- | --- | --- |
| **All Services in One Place** | **Health concerns beyond LMB professional care scope, required referrals** | So for the pre-eclampsia, you were followed here, but probably with someone else too.02: Yes, with the pre-eclampsia I had to give birth at Maison Naissance, but with the pre-eclampsia, they ended up sending me to the Jewish. | Interview #2 |
| **All Services in One Place** | **Positive experience with Parnter community resource for labour and delivery** | Yes it is very relaxed there, it feels like home, a nice place to give birth if you don’t have any complications. And very accommodating, the people, the staff, the environment.TA: Yes, *** showed me around, they try to make it homey, and comfortable | Interview #2 |
| **All Services in One Place** | **LMB external capacity to find solutions, refer to most economical alternative** | If they don’t have it, they will give you the information.02: Where to go, or the directions, or for example, if they don’t have the services, they will ask another organization, but you also, but through them, you also get a cheaper price, because if you don’t have your papers, like me, you pay so much money, so they are involved and make it less.\| | Interview #2 |
| **All Services in One Place** | **LMB service support** | ith my son, at the time, I was able to put my daughter back into school, even now, she is in school. 02: Now its government, through la Maison Bleue. | Interview #2 |
| **Always There** | **LMB unconditional support and availability to help their clients** | Well, my experience so far at La Maison Bleue has been a great one, and like, you can always call, they are just a phone call away, they never turn me down, they are always there to assist as you need, and it feels like home, a home away from home. | Interview #2 |
| **Appreciative of Freedom** | **Appreciative on new home country** | Canada is a good place to be. | Interview #2 |
| **Culture and Environmental Shocks** | **Weather Challenges** | sometimes it is cold but. | Interview #2 |
| **Family Seperation** | **Not having Family** | here, if you don’t have family here it is very difficult, | Interview #2 |
| **Family Values** | **Problem-solving learned from parents - Transmitting Values** | Yes, because in your country, you have independence from them, the way you were brought up, your parents bring you up, with, with seeing people, they prepare you for hard times, even if you are not going through hard times, you still find your way through, because you have been brought up to solve problems, to be independent, that prepares you for countries like this, the way you were brought up with your parents. | Interview #2 |
| **Family Values** | **Healthy means eating the family is eating nutritious food** | Yea – with my kids and myself, I try, I don’t give them junk food, once in a, like when they go to parties, I don’t buy junk food, or soda, I try give them a healthy balanced meal, because for health, because without health, you have nothing. | Interview #2 |
| **Family Values** | **Healthy means getting daily physical activity, such as walking** | I used to go to the Park every morning, but when it wasn’t cold . | Interview #2 |
| **Feels Like Home** | **MB Social Climate** | they seem to be likable people, nice people. | Interview #2 |
| **Feels Like Home** | **Positive feedback on LMB space and equipment** | But, when you come inside, you are amazed at the amount of space and material, wow, it is amazing. | Interview #2 |
| **Feels Like Home** | **Positive feedback on LMB** | Well, my experience so far at La Maison Bleue has been a great one, and like, you can always call, they are just a phone call away, they never turn me down, they are always there to assist as you need, and it feels like home, a home away from home. | Interview #2 |
| **Friends** | **Peer Support/Friendship, Spending time with friends** | going over to my friends house every weekend. | Interview #2 |
| **Friends** | **Close friendship from home-country in Canada** | From back home, we went to school together, so yea --. We knew each other from a little girl. | Interview #2 |
| **Friends** | **Children socializing with friend’s children** | Yes, her son is the same age as mine, he is 5 months older, but they have been growing together since baby.Oh yes, best friends, (Laughing), they are inseparable, they growing up, you swear they are twins! | Interview #2 |
| **Helps with All** | **LMB self-capacity to find solutions for clients** | No, they have everything, and they try to find everything for you, they always have a solution or try to find a solution, they make life a bit easier, because the time I met them – that’s when I am like (didn’t know) In Canada. | Interview #2 |
| **Helps with All** | **Most helpful LMB service; vaccinations for children** | the most helpful… I think are the vaccinations with the Nurse. Because, if you go to the CLSC, you have to wait a long time, here it is faster. | Interview #2 |
| **Immigration Process** | **Refugee Claimant Refusal and Loss of Rights** | when I did the refugee.. it was okay, because, but it is a long process, for timing process, but at least your getting somewhere, but when I didn’t get through, the challenges came, she had to stop school, I had to work past jobs, so it was really really frustrating. | Interview #2 |
| **LMB Challenges** | **Staff turnover at LMB, requiring become re-acquainted with personnel** | Faces sometimes change, you see a different person.Yes, by the time I was coming here, the social worker has changed, the doctor, the midwife, but *** has been here all the time, the nurses have been changing.. | Interview #2 |
| **LMB Challenges** | **Seeking assistance with immgiration outside LMB** | well you mentioned before that you did not really use the Social Worker services with immigration, housing advocacy (from socio-demographic form completion).. So who helps you with this?02: Lawyer, I have an immigration lawyer that is paid for by my husband, he is been really helping with this (He is sponsoring her for Permanent Immigration Status). | Interview #2 |
| **LMB Recommendation** | **Recommendation to add an ultrasound machine for pre-natal care** | Yes, they have services, that is the only thing, an ultrasound machine. | Interview #2 |
| **Maintaining Traditions and Language** | **Religion** | we go to Church to every weekend. | Interview #2 |
| **Maintaining Traditions and Language** | **Tradition of cooking specific dishes, continued in Canada** | I always cook, more like how I used to back home, (I don’t like what they sell), so I always cook more, how I used to.02: Yes, because there are little stores, where you can get the food, yes. So, I eat that, what I know, I don’t get the junk. | Interview #2 |
| **Navigation of System** | **LMB offering support, relieving stress, providing hope to clients** | since I have also came to La Maison Bleue, that’s when I started getting though my challenges, they are able to support you, let you know where to go, how to get stuff, they like are the ones that really help me, to, become, to have less stress, to want to be less worried, they give you hope, they give you hope, yes | Interview #2 |
| **Navigation of System** | **Informational Support-MB offering information** | : If they don’t have it, they will give you the information. | Interview #2 |
| **Pregnancy Stresses** | **Health-related concerns for birth outcomes** | Well so far, everything has been okay, the only thing is that when I was pregnant with her, she was premature, I had pre-eclampsia, but apart from that, she in in good health | Interview #2 |
| **Protection of Rights** | **LMB facilitation of educational services with precarious migrant status** | I came to Maison Bleue, when I was pregnant with my son, at the time, I was able to put my daughter back into school, even now, she is in school. | Interview #2 |
| **Seperation of Family** | **Not having family members in Canada and the difficulties** | here, if you don’t have family here it is very difficult, if you don’t, if you’re not strong, you want to run away, | Interview #2 |
| **Support and Love** | **Having family members to support adaptation in Canada** | 02: Yes, my family, family support. TA: So having your brothers here - | Interview #2 |
| **Support and Love** | **Family Involvement – Presence of Family Members, source of support** | having 2 brothers here in Canada, | Interview #2 |
| **Support and Love** | **Family Involvement – Love and support from family as source of strength, impacts health & well-being** | Well just the love, and support, that alone, impacts your health also. | Interview #2 |
| **Support and Love** | **Having family members to support adaptation in Canada** | As long as you have assistance, that you have people surrounding with love, with me, I have my two brothers here.TA: Oh, they were here before? 02: Yes, they have their papers, so I have two people I can count, without family members though, it can be very very challenging. TA: And your brothers have families as well?02: Yes, yes they have their families. | Interview #2 |
| **Support and Love** | **Stress relief from a supportive and providing partner** | But since I am with husband now, it has been really relaxing, I don’t have to worry, he is the one that has to think for me, I can breathe now. | Interview #2 |
| **Support and Love** | **Having family members to support adaptation in Canada** | Yes, my family, family support.TA: So having your brothers here -02: Yes it is a good thing> | Interview #2 |
| **Support and Love** | **Stress relief from a supportive and providing partner** | And your husband as well.02: Oh yes, he has been a good, a good good influence in our lives | Interview #2 |
| **Support and Love** | **Having family members to support adaptation in Canada** | Canada is a good place to be. As long as you have assistance, that you have people surrounding with love, with me, I have my two brothers here. | Interview #2 |
| **Support and Love** | **Family Involvement (Visits from persons overseas, coming to spend time in Canada)** | Yes, she came here for 8 months, | Interview #2 |
| **Support and Love** | **Partner Support** | but since I am with husband now, it has been really relaxing, I don’t have to worry, he is the one that has to think for me, I can breathe now. | Interview #2 |
| **Time - 2nd Baby, less stressed** | **Pregnancy Worries** | No just this baby, yeah, I was saying like, when I was pregnant with my son, I was so stressed out, because the Dad was not alive (well he was okay but?), but now I was happy and relaxed, and I am saying what is this? (Laughing) | Interview #2 |
| **Transnational Connections** | **Frequent communication with at-distant family members** | And your mom you are able to speak with her?Almost every day – and now with WhatsApp, we facetime all the time.So its easier, and its free. She is far, but she is a good support system. | Interview #2 |
| **Transnational Connections** | **Temporary visits from distant family members** | Has your mother ever thought about coming here?02: Yes, she came here for 8 months, but as you get older, you comfortable at home, she doesn’t like the cold, yea she is okay. | Interview #2 |
| **Transnational Connections** | **Visiting family members in home-country** | No I have not gone back, so hopefully, we can go home.Yes, it has been hard being away from her, other than that it is okay. | Interview #2 |
|  |  |  |  |

| **Categories** | **Code** | **Excerpt** | **Source** |
| --- | --- | --- | --- |
| **All Services in One Place** | External referral to CLSC from MB | oh non, pour ça, il faut aller au CLSC . | Interview #3 |
| **Always There** | Seeking assistance, information when child sick | quand ma fille est malade, j’appelle d’abord la Maison Bleue. | Interview #3 |
| **Always There** | Seeking advise from healthcare professionals about children | je demande toujours, avis de le médicéen, | Interview #3 |
| **Always There** | Asking questions to MB, seeking advice | je demande toujours, a ***, à **** (MD), | Interview #3 |
| **Appreciative of Freedom** | Appreciation for recieving-country, positive interactions with recieving population | ça va quand même, il y a la sécurité, on marche, on (pique? ***?) les personnes, du monde, c’est sécuritaire , c’est vraiment sécuritaire, à la Maison Bleue, tout va bien | Interview #3 |
| **Appreciative of Freedom** | Sense of belonging, support | , c’est vraiment sécuritaire, à la Maison Bleue, tout va bien , | Interview #3 |
| **Children as Purpose** | Goals; to have children, gives meaning, strength | je veux avoir mon enfant, | Interview #3 |
| **Children as Purpose** | For the children, gives meaning | quand je regarde ma fille, je veux d’être en bonne santé pour elle, si je suis malade, qui va s’occupe delle pour moi. | Interview #3 |
| **Children as Purpose** | For the children | : La raison c’est puisque ma fille. | Interview #3 |
| **Children as Purpose** | Maintaining self-health for children, children as purpose | Donc, c’est ça, quand je regarde, je dis; il faut que je sois en bonne santé, si je suis malade… | Interview #3 |
| **Connects Women** | LMB environment facilitates relationship building | Oui, J’ai aussi trouvé les amis aussi, comme.. | Interview #3 |
| **Connects Women** | Made friends through MB | on est là sur WhatsApp, comment ça va? Comment ça va les enfants? J’ai trouvé les amis à la Maison Bleue. | Interview #3 |
| **Culture and Environmental Shock** | Weather difficulties | quand ça fait froid | Interview #3 |
| **Culture and Environmental Shock** | Unfamilair new environment, immigration stress | tu ne connais pas Montréal, | Interview #3 |
| **Culture and Environmental Shock** | Weather challenges | je fais froid (pendant les mois du Mars?) ,  je besoin de retourner à l'école pour refaire le (recyclage),  je | Interview #3 |
| **Family Values** | Keeping healthy | Je vais un peu des sports, boire beaucoup de l’eau, ça c’est que je fais. | Interview #3 |
| **Family Values** | Passing on parental values to children | que j'ai parlé, ça c’est quoi je Transfer à mon enfant, donc, pour savoir vivre, pour dire de ne pas faire ça, de ne pas faire ça, donc, la même que je Transfer à mon enfant, donc, l’éducation que J’ai réussie dès mon parents, la moralité. | Interview #3 |
| **Family Values** | Strategies to maintain healthy | , je sais de ne pas manger beaucoup de. des trucs, comme sucre, oui, bon.  Je | Interview #3 |
| **Family Values** | Passing on learned parenting values to children | manger les legumes et ma mere, je (ne pas) normallement mange toutes les legumes, | Interview #3 |
| **Family Values** |  | Oui, ma mere a moi, elle voulait, elle a dit; pour manger des legumes, les autres choses sont pas bonnes pour la santé, ça fait grossi, ça donne les maladies, c’est ça qu’elle a me disait tout temps. | Interview #3 |
| **Gives Hope** | LMB building confidence, empowering them | pour avoir la confiance, | Interview #3 |
| **Having Help and Support** | Access to healthcare services, benefits | comme les soins médicaux, c'est facile, c'est le gouvernement qui s'occupe de ça, et les enfants, oui c'est facile. | Interview #3 |
| **Helps with All** | MB Interdisciplinary team providing instrumental care | Les services de vaccinations, pour mon enfant, le médicéen de famille , | Interview #3 |
| **Helps with All** | Group-interventions, facilitate learning | , et bien dans le groupe postnatal, on apprend des choses, pour apprendre des trucs, comme aujourd’hui, | Interview #3 |
| **Helps with All** | Increased access to healthcare services through LMB, versus other routes | Les services de vaccinations, pour mon enfant, le médicéen de famille, c’est utile, puisque pour avoir un médicéen de la famille,  ce | Interview #3 |
| **Helps with All** | MB Assistance through pregnancy and birth | Quand, jetais enceinte, quand J’ai accouché, donc, c’est la Maison Bleue, | Interview #3 |
| **Helps with All** | Instrumental support, vaccines for children | Les vaccinations, puis le suivi avec l’infirmière. | Interview #3 |
| **Immigration Process** | Migration status limitations | de côté négatif quand J’ai pas encore le statut, quand tu fais une demande, | Interview #3 |
| **Immigration Process** | Immigration-related stress, finding housing quickly | il faut que tu cherches le logement vite , | Interview #3 |
| **Immigration Process** | Migration stress; find housing (unfamiliar, weather-challenges) | Je serais la pendant deux semaines, mon, il faut que je trouve un logement, | Interview #3 |
| **Immigration Process** | Migration challenge; education recognition | je besoin de retourner à l'école pour refaire le (recyclage), | Interview #3 |
| **Immigration Process** | Migration status restrictions/limitations | J’ai pas le droits, donc J’ai pas le droit, donc, (lauses), c'est ça qui est difficile pour moi, je pas droit, | Interview #3 |
| **Immigration Process** | Financial challenges, requirement to pay | je dois faire d'abord permis de payer cher . Je pas encore les moyens, donc, c’est, c'est juste ça, les choses négatifs pour moi, pour le moment. | Interview #3 |
| **Immigration Process** | Detention upon arrival, migration-related stressess | j'ai passé, un mois a centre de détention du Laval | Interview #3 |
| **Immigration Process** | Arriving to Canada, during pregnancy | pendant jetais enceinte, | Interview #3 |
| **Immigration Process** | Difficulties after first arriving | alors ce n’était pas facile pour moi, pas facile pour moi, | Interview #3 |
| **Immigration Process** | Conflict between own plan + recieving country advice? | Jai voulais avorter. (? or they wanted me to abort?) | Interview #3 |
| **Isolation** | Isolation, lack of social support | Je n’ai pas beaucoup des amis. | Interview #3 |
| **LMB Outcome** | Appreciation for LMB and their work/help | , la Maison Bleue, oui la Maison Bleue aide beaucoup. Quand je ne sais pas de faire, j'appelle la Maison Bleue, je parle avec la travailleuse sociale, la Maison Bleue m’aide beaucoup. Beaucoup, beaucoup, beaucoup. | Interview #3 |
| **LMB Outcome** | Gratitude for LMB | , la fondatrice c'est une génie, oui, c’est une génie, faire quelque chose pour les familles migrantes, les gens qui n’ont pas les papiers, donc c’est vraiment, c’est une génie, pour moi c’est génie (Baby crying, she focuses on her). | Interview #3 |
| **LMB Recommendation** | MB recomendation? | Si la Maison Bleue, avoir des chambres pour les femmes qui viennent accoucher  ( | Interview #3 |
| **LMB Recommendation** | Recommendation to MB : Assistance with immigration docuementation | Oui, la Maison Bleue, peuvent nous aidons aussi avec les papiers immigration, | Interview #3 |
| **LMB Recommendation** | Recommendation to LMB : Assistance from Immigration-Lawyer for documentation and process | TA : Comme un avocat dans immigration? 03 : Oui, oui. Ici à la Maison Bleue | Interview #3 |
| **LMB Recommendation** | Recommendation to LMB : Referrals, letters of reference for access to affordable housing | TA : Alors, le logement social, ou un référence…03 : Oui, le référence surtout, oui, ils peuvent faire comme | Interview #3 |
| **LMB Recommendation** | Recommendation to LMB : Area inside for strollers; get cold in Winter, difficult for children | 03 : Quand il neige, il y a des pas de espaces pour les poussettes. | Interview #3 |
| **Protection of Rights** | Migration-status restrictions in accessing healthcare services | Surtout pour moi, surtout pour moi, encore avec des pas de carte assurance maladie, mais telle la maison bleue, J’ai un médicéen de la famille. | Interview #3 |
| **Protection of Rights** | Barrier in accessing healthcare services, after arrival | , je suis allé à CLSC, je présente mon papier de demander daille, et ils ont dit; ça c’est pas l’assurance maladie! (Raises voice). Donc, ça m’a dit quoi, Jaime pas la CLSC comme Je n’ai pas de carte l’assurance maladie,  mais à la Maison Bleue, je suis à l’aise, je montre rien. | Interview #3 |
| **Support and Love** | Family support as source of strength | ça m’aide beaucoup, moralement, pour surmonter des défis | Interview #3 |
| **Support and Love** | Family time spending together | le temps ensemble , | Interview #3 |
| **Support and Love** | Instrinsic family strengths; time together, communication | , la communication, oui le temps passer avec en famille, | Interview #3 |
| **Support and Love** | Family closeness as stregnth | L’ambiance familial oui , | Interview #3 |
| **Transnational Connections** | Maintaining transnational relationships | Ok, et vous parlez avec vos parents? 03: Oui, oui. | Interview #3 |

| **Categories** | **Code** | **Excerpt** | **Source** |
| --- | --- | --- | --- |
| **Agency** | Having autonomy | je sais que j'ai parlé sur la solidarité, | Interview #4 |
| **Agency** | Becoming familiar with available services with time | il fallait chercher, fallait demander et toute ca, maintenant c'est une chose je ne fais plus, donc, ca devenir facile pour moi, | Interview #4 |
| **Agency** | Becoming familiar with available services with time | je sais laquelle autobus pour le prendre, le métro pour arriver à la maison bleue. Aujourd'hui quand me donne une adresse, il faut que j'aille sur google, je donne l'itinéraire, je demand l'itinéraire, comment faire pour y arriver et je sais me sortir. Il y a plus en plus des organismes, | Interview #4 |
| **Agency** | Gaining competencies in reciving country, navigation | je sais laquelle autobus pour le prendre, le métro pour arriver à la maison bleue. Aujourd'hui quand me donne une adresse, il faut que j'aille sur google, je donne l'itinéraire, je demand l'itinéraire, comment faire pour y arriver et je sais me sortir. Il y a plus en plus des organismes, | Interview #4 |
| **Agency** | Demonstrating personal agency, willingness to learn – gain professional competencies | maintenant sur le plan professionnel, ça reste un difficulté.  Et | Interview #4 |
| **Agency** | Leaving the house, overcoming isolation | Oui, c'est ca, il faut que tu sors pour voir les gens, | Interview #4 |
| **Agency** | Social Support, feels integrated socially | Puisque je fais déjà, sur le plan sociale, | Interview #4 |
| **Always There** | LMB availability for clients, willingness | , il y a toujours quelqu'un disponible, il y a toujours un oreille, on peut se poser sa tête,  lorsque vous allez à la maison | Interview #4 |
| **Busy-ness** | Difficulty after arrival, navigating, orientating self to new environment | comment me prendre et toute ca avec un enfant, et le grossesse, et le froid, c'était, tout sauf pénible, quoi donc c'était la pire des choses, c'était un vrai calvaire. Il fallait courir à gauche pour telle démarche, il fallait courir à droite pour telle chose, c'était un nouveau, un nouvel univers et c'était vraiment difficile pour moi, très difficile. | Interview #4 |
| **Children as Purpose** | Children as reason, motiviation for parents *Source of strength | je pense que je me dis c'est pour la bonne cause et pour la bonne cause c'est quoi? C'est pour mes enfants. | Interview #4 |
| **Children as Purpose** | Providing reason, search for better oppurtunities in new country | maintenant mes enfants son père mais je me dis que c'est une bonne pour une bonne | Interview #4 |
| **Children as Purpose** | Goal of family reunification, source of strength | compte on finira par nous unir et être ensemble comme une famille, c'est ce qui me donne la force. | Interview #4 |
| **Children as Purpose** | Sense of purpose | enfants. Quand je le vois, ça me ça me fait la joie, ça me donne encore plus de courage de tenir le coup jusqu'à atteindre l'objectif. | Interview #4 |
| **Connects Women** | LMB facilitating relationship building, within similar ethnic communities | je ne connais pas personne?, mais a grâce de la maison bleue, je fais leur connaissance et de faire connaissance au téléphone et tout ça. | Interview #4 |
| **Culture and Environmental Shocks** | Differences between recieving country and home-country, acculturative stress? | parce que le Canada et mon pays c'est deux mondes différents. | Interview #4 |
| **Culture and Environmental Shocks** | Acculturative stress, differences in culture | peu la solidarité, ici il y a que la solitude, où on peut rester pendant des jours il y a personne qui frappe à votre porte, et quand vous sortez pas, vous voyez pas les gens, ou même les voisins qui à côté, donc c'est des choses ici, que je n'ai pas vécu dans mon pays, donc c'est ça. | Interview #4 |
| **Culture and Environmental Shocks** | Unfamiliar with how to access services | parce que je connaissais pas, je connais rien, | Interview #4 |
| **Family Seperation** | Seperation of family | j'ai laissé mes deux enfants et mon mari pour me retrouver ici. | Interview #4 |
| **Family Seperation** | Goal of family reunification | rapport de la séparation avec les enfants et il faut faire les démarches pour venir ici, alors ca c'est les difficultés? | Interview #4 |
| **Family Seperation** | Challenges of single-parenthood | les jours, jouer les deux rôles; père et mère, | Interview #4 |
| **Family Seperation** | Prenting Concerns, Single-parent, difficulties, reduced support | je suis une mère monoparentale, c'est encore difficile, | Interview #4 |
| **Family Seperation** | Family seperation, difficulties | par rapport de la séparation avec les enfants et il faut faire les démarches pour venir ici, alors ca c'est les difficultés ? | Interview #4 |
| **Family Seperation** | Seperation of Family | moi avec la distance qui me sépare avec mes enfants et mon mari,   qu'on | Interview #4 |
| **Family Values** | Passing on parental values to children | , je parlerai de respect des normes sanitaire, et sur le plan mental je parlerai, de des choses qui nous permettent de garder notre moral en place. | Interview #4 |
| **Feels Like Home** | Gratitude for LMB, closeness with LMB, strong relationship | j'ai toujours dit que la maison bleue finalement, je l'ai vu et est considéré comme ma seconde famille. | Interview #4 |
| **Gives Hope** | LMB accompanying clients through problem-solving | l'assurance et m'accompagner pour que cela et c'est ce qu'ils ont fait c'était pas seul et pour que ca fait arrêter . | Interview #4 |
| **Having Help and Support** | Community resources, access to food | C'est un magasin alimentaire pour les nouveau arrivants, ca aide. | Interview #4 |
| **Having Help and Support** | MB external referral, partnership with other organizations | et la société du logement,  donc toute le monde avait aidé | Interview #4 |
| **Having Help and Support** | Children going to daycare, use of services | comme la première elle va déjà la garderie, le deuxième bientôt il va devoir aussi y aller.  Aussi | Interview #4 |
| **Helps with All** | Offering solutions | je me suis retrouvé que quels que soient les besoins je pouvais le poser et, il y avait des solutions qui pouvait être résolu. | Interview #4 |
| **Helps with All** | Finding solutions | , et j'étais devant mon médecin, et la sage femme, et tout suite, elle m'avait pris dans leurs bras, et c'était le réconfort que je ressentir, | Interview #4 |
| **Helps with All** | Context MB, goals to provide comprehensive family care, restrictions in time and resources to provide | 04:  Ca je sais pas, si le gouvernement donne, ou les gens donne, je sais que ça vient mais, j'aimerais vient encore beaucoup plus pour la maison bleue. Parce que moi je trouve que la maison bleue donne beaucoup, quelle est la même (n'aime) pas? C'est l'impression que moi jai. C'est … comme la maison bleue est un mere, la mere veut toujours donner les meilleures pour ces enfants, même si elle peut pas, elle puissie a les dernières forces, pour donner pour ces enfants. Moi, jai demanderai pas à la maison bleue pour améliorer ces services, parce que ce sont bons, mais je demande que qui peuvent, pour donner aide à la maison bleue pour qu'elle continue à rendre ce service. puisqu'on se sent toujours à la maison donner les meilleurs, même si il y a des limites, mais vous sentez qu'elle veut donner. | Interview #4 |
| **Helps with All** | Comprehensive LMB approach. Holistic approach to clients and care | mais quand on ouvert la paquet maison bleue, i y a tout dedans. | Interview #4 |
| **Helps with All** | Ongoing learning from LMB activities | comme il y a une activité, je dis, wow je savais pas tout ça, et grâce à la maison bleue, on sait. Il | Interview #4 |
| **Helps with All** | Instrumental Support, access to healthcare | que c'était juste pour soins médicaux | Interview #4 |
| **Immigration Process** | Migration stress; finding employment | Il faut que je cherche le travail ou encore il faut que je cherche à avoir commandé à faire la mise à niveau de ma connaissance, par rapport à mes études de pays et ici, pour chercher un emploi.  Donc | Interview #4 |
| **Immigration Process** | Immigration Stress, education recognition | Il faut que je cherche le travail ou encore il faut que je cherche à avoir commandé à faire la mise à niveau de ma connaissance, par rapport à mes études de pays et ici, | Interview #4 |
| **Immigration Process** | Traumatic experiences, country of origin | que j'ai des problèmes avec mon pays avec la politique de mon pays c'est comme ca quand je suis venue un réfugié ici. | Interview #4 |
| **Immigration Process** | Trauma in home-country, concerns for family still there | politique et tout ça et quand là-bas ça ne marche pas bien, tu peux dans ses peurs et tout ça, qu'est-ce qui va arriver et tout ça, ou encore si les enfants sont pas bien, tu apprends ça, on se sent pas, ça | Interview #4 |
| **Isolation** | Isolation | ça signifie les devis que je puisse surmonter, vivre seul, | Interview #4 |
| **Living in Underprivileged Areas?** | Housing related difficulties | j'ai une nouvelle déménager, avec un voisin, ou j'ai tellement de problèmes, | Interview #4 |
| **LMB Outcome** | Gratitude for MB support | je considère la maison bleue ma seconde famille . | Interview #4 |
| **Maintaining Traditions and Language** | Importance of religion, source of strength | , par exemple là je vais à l'église , | Interview #4 |
| **Positive Attitude** | Positive emotional regulation | c'est vraiment difficile, chaque quand je me réveille, (je me dis wow) les jours passe, ca avance, j'arrive à m'en sortir | Interview #4 |
| **Pregnancy & Parenting Stresses** | Single-parent, difficulties | je suis une mère monoparentale, c'est encore difficile, | Interview #4 |
| **Pregnancy & Parenting Stresses** | Difficulty upon arrival, arriving with children and being pregnant | j'étais enceinte. j'avais une enfant qui avait une année et demie. Et c'était totalement difficile, | Interview #4 |
| **Pregnancy & Parenting Stresses** | Parenting Responsibilities, children growing older, advancing in school | puisque maintenant, les enfants grandissent, comme la première elle va déjà la garderie | Interview #4 |
| **Pregnancy & Parenting Stresses** | Parenting challenges | retrouver comme c'est avec deux enfants plus que l'autre avait une année et demi. | Interview #4 |
| **Pregnancy & Parenting Stresses** | Psychological stress, difficulties coping | je vous rappelle même pas une fois quand j'étais enceinte, j'ai fait un crise, et je ne fais rien, mais j'ai fait un crise, j'ai tombé…. Donc c'est que la tête n'arrive pas. | Interview #4 |
| **Religion** | Religion | consulter me soutient, c'est Dieu | Interview #4 |
| **Support and Love** | Family strength; communication | la communication, | Interview #4 |
| **Support and Love** | Family closeness as strength | - des mes enfants est la seule chose qui peut me permettre de les sentir encore près de moi, | Interview #4 |
| **TIME - Coping Better** | Coping better over time, takes time to adapt | je sais ca va prendre belle du temps. | Interview #4 |
| **Transnational Connections** | Maintaining translational bonding, source of strength | temps au téléphone avec eu, de parler, parle du rein, bien avec la distance, avec la communication,  on peut parler ce qu'on a fait pendant la journée, ca aide beaucoup. | Interview #4 |
| **Transnational Connections** | Maintaining transnational family bonding, source of strength | t que je passe peut-être deux ou trois jours sans parler avec, je suis pas bien du tout. | Interview #4 |
| **Transnational Connections** | Transnational bonds, maintaining communication as source of strength | je dis c'est la communication avec mes enfants, | Interview #4 |
| **Varied Team** | Holistic team, interdisciplinary approach | , aussi  à la sage femme et la travailleuse sociale qui c'était tout le monde, | Interview #4 |
| **Varied Team** | Gratitude for interprofessional team members all present at LMB | , comme  je vous avais parlé au début, si il ya les défi de la profession, je parle avec la travailleuse sociale, et elle me donne un rendez-vous, comment ca va prendre . | Interview #4 |

| **Category** | **Codes** | **Excerpt** | **Source** |
| --- | --- | --- | --- |
| **Agency** | **Autonomy + Personal Agency** | I am more independent than before, I have my drivers license now, before I needed my husband for everything, but now it is good . | Interview # 5 |
| **Agency** | **Willingness to learn** | When I arrived here, I began French classess. | Interview # 5 |
| **Agency** | **Willingness to learn** | to find training for children, chidlren education | Interview # 5 |
| **Agency** | **Willingness to Learn** | After I started studying French. | Interview # 5 |
| **Agency** | **Willingness to Learn** | Yes, it has improved . I am more autonomous than before . | Interview # 5 |
| **Agency** | **Willingness to Learn** | yes, I looked for activities, to have training, to learn about the children , | Interview # 5 |
| **All Services in One Place** | **Instrumental Support** | it is good to get appointments with the doctor . | Interview # 5 |
| **All Services in One Place** | **Instrumental Support (Fast Service)** | Yes, the services are faster . | Interview # 5 |
| **Always There** | **MB Approach - Available** | it is at your disposability. | Interview # 5 |
| **Children as Purpose** | **Children as Reason** | Yes my children, I must be in good health for them.  I | Interview # 5 |
| **Children as Purpose** | **Children as Reason** | I must be in good health for them.  I | Interview # 5 |
| **Family Seperation** | **Seperation of Family, Parenting Challenges** | yes to have children and not to have help from the family. | Interview # 5 |
| **Family Values** | **Passing on Values** | The children to be in good personality, to be good in society. Passing the values to the children. I | Interview # 5 |
| **Family Values** | **Values of Family** | The family is very important, | Interview # 5 |
| **Family Values** | **Passing on Values** | Well we try, to, to be in good health, and that they are in good personality, for them, and for society. This helps, to be a good person. | Interview # 5 |
| **Friends** | **Peer Support** | so I go with friends | Interview # 5 |
| **Friends** | **Social Support, Had many friends** | He had many friends already, a lot of family,  so | Interview # 5 |
| **Friends** | **Peer Support, speaking to other Mothers, similar situations** | Speak to other mothers | Interview # 5 |
| **Helps with All** | **Community-Resources** | They organize many things for the children, to discover Montreal, my husband works on Saturday, so I go with friends to these events. | Interview # 5 |
| **Immigration Process** | **Discriminiation?** | when you fall sick, you know/feel you are an immgrant , but | Interview # 5 |
| **Immigration Process** | **Immigration-Related Difficulties** | For my husband, it is difficult, he has a Doctorate in Pharmacology, he tried many times, did many interviews, but he was not selected, he did searching with Emploi Quebec, he works now at a restaurant, this is something with immigration that is not good, I really like Canada, but they want people that have studies but then after, they leave us just like that. But if we return to Morroco, we can work, that is why many people return, because there it is better. | Interview # 5 |
| **Isolation** | **Seperation of Family, Parenting Challenges, loss of traditional forms of family support for child-rearing** | yes to have children and not to have help from the family. | Interview # 5 |
| **Living in Underprivileged Areas** | **Poor Housing, De-favorised Neighbourhoods** | you may end up living in de-favorised areas, like in CDN | Interview # 5 |
| **Living in Underprivileged Areas** | **Discriminiation?** | The problem is that they don’t accept children | Interview # 5 |
| **Living in Underprivileged Areas** | **Poor Housing** | here in CDN, the problem is, yes, you will end up in defavorized areas . | Interview # 5 |
| **LMB Limitation** | **MB Limitation** | For children, it is just to 5 years old . | Interview # 5 |
| **LMB Limitation** | **MB limitation** | It is difficult to see the physician - I have to go to the CLSC. It will take about 2 weeks. If urgent, they will tell you to go to the CLSC. I know it is busy, many appointments. So there is no service urgence here. But when you don’t have a car, it can be hard, it takes time . | Interview # 5 |
| **LMB Limitation** | **MB limitation** | if the LMB, it is only to age 5, because here, right now, the ones that are bigger, I don’t find anything here for them, I have to look elsewhere. | Interview # 5 |
| **LMB Limitation** | **MB limitation** | More information on subjects, because the subjects we hear about post-natal, we repeat, we repeat, it does not change. | Interview # 5 |
| **LMB Limitation** | **MB limitation** | Well, just it is difficult to see the doctors. It takes long. I have to go to the CLSC. If you are sick, you don’t have a car, and it is close here, practical to come here. | Interview # 5 |
| **LMb Outcome, Gratitude** | **MB Outcome, Gratitude** | I have had a nice experience with LMB, I like it a lot, I hope it will continue, to help me. | Interview # 5 |
| **LMB Recommendation** | **MB Recommendation** | More information on other subjects, the post-natal groups, the information is the same, it repeats, it repeats. To do updates on the information. We don’t the schools, there are many schools, with the public schools, we don’t know them, it is only when they start going that we discover them. Not like at home. So I want training on the school systems. The educator does not give information on the schools. After the children, we do the following. It is very important, the schools. People immigrate here for the education and the schools, so it is important. | Interview # 5 |
| **LMB Recommendation** | **MB Recommendation** | Daycare service here. For a reasonable price. | Interview # 5 |
| **LMB Recommendation** | **MB Recommendation** | but we don’t know about schools or anything, it is not like where we are from, there are a variety, public schools, many different ones, so we don’t know. It is only after the children go to school that I learn about the options. I don’t have that information. | Interview # 5 |
| **LMb Recommendation** | **MB Recommendation** | A service, a daycare service here. For a reasonable fee. | Interview # 5 |
| **Maintaining Traditions and Language** | **Cultural Maintenance** | All of the children speak Arab, this is important for the culture. | Interview # 5 |
| **Maintaining Traditions and Language** | **Cultural Maintenance** | I also do Morroccon dishes, they know them well. From Morroco, from everywhere. | Interview # 5 |
| **Maintaining Traditions and Language** | **Cultural Maintenance, keeping traditions such as language** | we try to keep the traditions, to continue them. We speak Arab, we want to keep the good Arab for them. | Interview # 5 |
| **Maintaining Traditions and Language** | **Cultural Maintenance, maintaining language in family, important for identity** | Yes, exactly. It is important for them when they return to Morocco, to be able to communicate and understand. | Interview # 5 |
| **Maintaining Traditions and Language** | **Maintaining Transnational Relationships** | Whatapp to keep in touch with them. | Interview # 5 |
| **MB Limitation** | **MB limitation** | My other children are bigger, I don’t find the information for them, I must find this information elsewhere. | Interview # 5 |
| **Navigation of System** | **Instrumental Support** | They helped me get appointments, | Interview # 5 |
| **Pregnancy & Parenting Stresses** | **Parenting responsibilities** | Having children | Interview # 5 |
| **Support and Love** | **Family Involvement** | help from husband as well, he is only works 4 days, and not 8 hours, about 6 hours, he is available for the children. | Interview # 5 |
| **Support and Love** | **Family Involvement + Support** | My husband was there before me, | Interview # 5 |
| **Support and Love** | **Family Invovlement – Time Together** | We find activities, to leave the house, everyday we go to the park | Interview # 5 |
| **Support and Love** | **Family Involvement and husband presence meant having social support and reducing those difficulties (isolation..)** | so I did not have many difficulties at the social level . | Interview # 5 |
| **TIME** | **Time - Ongoing Challenge** | The status has not changed, he has worked for every long in a restaurant, and continues today. | Interview # 5 |
| **TIME** | **Time Factor – Needed Help** | , I had to wait for my husband to help me with everything | Interview # 5 |
| **TIME** | **Time - Willingess to Learn – Learning Over Time** | For the resources, yes, I looked for activities, to have training, to learn about the children , education, that helped me a lot, | Interview # 5 |
| **TIME** | **Time Factor – Needed Help** | , I had to wait for my husband to help me with everything , | Interview # 5 |
| **TIME -** | **Time - Ongoing Challenge** | The status has not changed, he has worked for every long in a restaurant, and continues today. | Interview # 5 |
| **Varied Team** | **MB Approach** | The team is varied, they are available. | Interview # 5 |

| **Category** | **Code** | **Excerpt** | **Source** |
| --- | --- | --- | --- |
| **Agency** | **Autonomy** | You need to leave the house, | Interview #6 |
| **Agency** | **Willingness to Learn** | I am also studying to become a nurse, at the college level, this program takes 3 years. | Interview #6 |
| **Agency** | **Willingness to Learn? Autonomy** | you can go and discover, me I wanted to discover . | Interview #6 |
| **Agency** | **Willingness to make friends, build relationships** | I am already here, it means I need to be strong , to exit the house, to meet other mothers, to make friends , | Interview #6 |
| **Agency** | **Autonomy** | You need to leave the house, | Interview #6 |
| **All Services in One Place** | **Holistic Care** | Just from the time I came, I did not have to look for other organizations, with MB, they | Interview #6 |
| **Always There** | **Accompainement -Emotional Support** | Certainly, I arrived at the hospital, the doctor came, and came back just until a gave birth, she was there, and she really really supported me and encouraged me, just until she saw the baby, and then she left. The family doctor who works at MB. | Interview #6 |
| **Always There** | **Approach** | Yes, the MB, you can ask them, they have solutions . | Interview #6 |
| **Always There** | **MB Accompagnement** | Yes, followed the pregnancy, had accompaniment of the doctor,  I told him, it was wonderful. | Interview #6 |
| **Appreciative of Freedom** | **Positive Experiences with Receiving Country + Population (Sense of Belonging, Gratitude, Safety, Freedom)** | I want to say thank you to Immigration Canada, it welcomes me here. | Interview #6 |
| **Appreciative of Freedom** | **Gratitude to be in Canada** | , I am happy to be here. | Interview #6 |
| **Appreciative of Freedom** | **Gratitiude for being in Canada** | , I was very happy to be welcomed, welcomed in the sense that,  I | Interview #6 |
| **Busy-ness** | **At beginning, time factor – being busy, running around** | there is a lot of things to do, sometimes I would forget to eat, you have to run around. | Interview #6 |
| **Children as Purpose** | **Children as Reason** | My 2^nd^ girl, I love her – she is 2 years old. She is always happy tho, she is like me, always smiling as well. She is always running, jumping around. She has good humor. | Interview #6 |
| **Children as Purpose** | **For the children** | I must stay, to raise the family. | Interview #6 |
| **Children as Purpose** | **Children as Reason** | so I wanted to join him here and start a family.  It | Interview #6 |
| **Children as Purpose** | **Children as Reason – Partner education for better employment** | , it is worth it, it will help the family, | Interview #6 |
| **Connects Women** | **Peer Support** | but I have friends, that I have met here, at MB. We discuss, we hang out. These are friends I have met here, at MB. We visit each other, when possible. When I had my daughter, we celebrated, we made food, cooked. They also live close to me, some are far, in Laval, other far places. But it is amazing to have them as well. | Interview #6 |
| **Connects Women** | **MB environment, meeting others** | , but I have friends, that I have met here, at MB. | Interview #6 |
| **Culture and Environmental Shocks** | **Acculturative Stress** | not like in Africa, where you see your neighbour, you walk around, it is open there. | Interview #6 |
| **Culture and Environmental Shocks** | **Aculturative Stress** | No one adapts to winter here (laughing).  You | Interview #6 |
| **Culture and Environmental Shocks** | **Weather challenges, acculturative stresses** | it was very difficult, the climate , | Interview #6 |
| **Culture and Environmental Shocks** | **Acculutative Stresses – Lost sense of community – individualism in Canada, 🡪 linked to isolation** | because in Africa, it is not like here, it is open there, you wake up in the morning, here you don’t see your neighbour, there you see them, you ask them is everything going well? We share everything, Here you stay at your house, you have nothing to share, you don’t have anyone to share with. | Interview #6 |
| **Culture and Environmental Shocks** | **Acculturative Stresses, differences in community, home-country to Cameroon** | Yes, independent, individual – what is happening with that? I ask myself why am I here? The individualism. What is happening? The people when you pass them, they don’t say hello. If you do, they will look at you strangely . | Interview #6 |
| **Family Seperation** | **Family Involvement** | If your mother comes, it will be easier with your children? Oh yes. I want to know the best way to bring her here. | Interview #6 |
| **Family Seperation** | **Seperation of Family** | Otherwise, yes it is very difficult for the family you have left. | Interview #6 |
| **Family Seperation** | **Seperation of Family** | I don’t have any family here , | Interview #6 |
| **Family Seperation** | **Seperation of Family** | Otherwise, yes it is very difficult for the family you have left. | Interview #6 |
| **Friends** | **Friendship support, maintaining freindships** | 06: They also live close to me, some are far, in Laval, other far places. But with knowing the metro system, it is wonderful. | Interview #6 |
| **Having Help and Support** | **Education services for children** | 06: Second child in primary school. | Interview #6 |
| **Helps with All** | **Informational Support** | They explained the risks and the advantages, there are forms you need to sign to give birth naturally. | Interview #6 |
| **Helps with All** | **Instrumental support, support through giving birth** | with the birth of my second girl, it was very very good, it went well, with the MB, it was intense | Interview #6 |
| **Helps with All** | **MB benefit for clients, reduce time and increase accessibility to services** | If you want a woman to attend your birth, they can arrange for that. If you need a medication, they can arrange for it quickly. | Interview #6 |
| **Immigratin Process** | **Immigration Process Difficulties** | The application for immigration, no. | Interview #6 |
| **Immigration Process** | **Immigration Related challenges** | The application for immigration, I would say no, because, as a new (migrant?), it was very difficult | Interview #6 |
| **Immigration Process** | **Reason to Leave Home Country** | the conditions (in Cameroon) were unfavourable. | Interview #6 |
| **Isolation** | **Reduced Social Support, Isolation** | I didn’t know anyone, he was only the person I knew in Canada . I was alone with the children , | Interview #6 |
| **Isolation** | **Isolation, at home alone** | TA: And your husband is gone all day? 06: All day, and night, | Interview #6 |
| **Isolation** | **Isolation, staying at home alone with children** | you have to stay all alone, I was alone with the children  , | Interview #6 |
| **Isolation** | **Isolation in community, lack of support** | no one will help you, | Interview #6 |
| **Living in Underpriviledged Areas** | **Poor Housing, Defevorized Areas** | the de-favourable areas and environment, | Interview #6 |
| **LMB Outcome** | **MB Gratitude** | LMB for me, its perfect, its perfect to me. Grace a LMB, I have found my smile, during my pregnancy, just to the end. I am asked If I am okay, if there are other problems. It is genial. | Interview #6 |
| **LMB Recommendation** | **MB recommendation** | Maybe, after the studies, someone who can help find employment. I think the SW can help you… “I don’t know”. Because I want after, training for finding work, someone who can orient me on this. | Interview #6 |
| **Love and Support** | **Partner support, family involvement** | My husband is there , | Interview #6 |
| **Maintaining Traditions and Language** | **Cultural Maintenance** | I do some more traditional medicines, that I have learned from my origin. | Interview #6 |
| **Maintaining Traditions and Language** | **Maintaining transnational relationships** | also my mother who supports me a lot, even with distance, at least 2-3 times per month, | Interview #6 |
| **Maintaining Traditions and Language** | **Seperation of Family** | . I also have my sister. TA: In Cameroon? | Interview #6 |
| **Maintaining Traditions and Language** | **Maintaining transnational relationships** | 06: My brother yes, he is on social media, like WhatsApp, I see him… with face time. | Interview #6 |
| **Maintaining Traditions and Language** | **Maintaining traditions, celebrating a birth** | it is a tradition to celebrate, so we celebrated, we made food, we cooked. | Interview #6 |
| **Maintaining Traditions and Language** | **Cultural Maintenance, traditional medicines learnt from home-country** | , I do some more traditional medicines, that I have learned from my origin. | Interview #6 |
| **Navigation of System** | **Continuity of Care/Holistic** | Did MB help you find schools? They helped me, but I selected the schools that were right beside me. | Interview #6 |
| **Navigation of System** | **Contuinity of Care - Referrals** | Formations des visits, that have helped me. An association of women, a group of women, who have migrated here, that are difficulty here, I had a visitor, that comes to the house, about 3 hours, I can sleep, I can study. It is the MB that referred me to them. With my first, I used this service. Formations des visites. | Interview #6 |
| **Navigation of System** | **Exsternal Referrals, help with finding schools for children** | TA: Did MB help you find schools? 06: Yes, They helped me, | Interview #6 |
| **Positive Attitude** | **Willingess to Learn…Autonomy** | you must remain calm, ask the questions – what will I do – ask yourself these questions | Interview #6 |
| **Positive Attitude** | **Time – Over time – Challenges overcame** | it is very difficult. But now, it is going better. But now it is going well,  I | Interview #6 |
| **Positive Attitude** | **Cultural value to be confident – personal agency** | 06: For culture, I would say, to have confidence in yourself, that is it. | Interview #6 |
| **Positive Attitude** | **Keeping Healthy, maintaining a positive attitude towards challenges and stresses** | For me what I do to keep healthy, is to just smile all the time, that is my secret, even if you are anger, you must remain calm, and relax. | Interview #6 |
| **Positive Attitude** | **Willingess to Learn…Autonomy** | 06: Yes, remain calm and ask the questions – what will I do – | Interview #6 |
| **Positive Attitude** | **Positive Attitude** | it impacts the physical and mental health. I think the smiling is the best gift I can give. | Interview #6 |
| **Pregnancy & Parenting Stresses** | **Emotional Support…Esteem Support** | Really positive experience here, I don’t need to find a midwife that can follow my whole pregnancy, I was really scared during my 2^nd^ pregnancy, because my first was done by caesarean, and now I am in a new country, I was scared, but Valerie said no, and encouraged me during the pregnancy, it was amazing  . | Interview #6 |
| **Support and Love** | **Coming to live with Partner, family involvement** | Me I left Cameroon because my husband is here. | Interview #6 |
| **TIME = Coping** | **Over time, adaptation and increased coping/relaxation with new situation** | 06: But now, not rosey, but I would say it is going well. Well enough. | Interview #6 |
| **Transnational Connections** | **Transnational Relationships** | My mother who supports me a lot, even with distance, at least 2-3 times per month, even if the conversation is not long, I also have my sister, in Cameroon, who I like a lot, who also supports me. I have brother as well. They are part of my support. My brother yes, whatsapp, with face time. So yes, communication. | Interview #6 |
| **Varied Team** | **Holistic Care** | , when I heard about, they have a SW, a nurse, a midwife, a family doctor, it is genial – all of the team here . | Interview #6 |
| **Varied Team** | **MB Instrumental Support, providing access to healthcare services** | that I could also find a family physician | Interview #6 |

| **Categories** | **Codes** | **Excerpt** | **Source** |
| --- | --- | --- | --- |
| **Agency** | **Demonstrating autonomy, coming alone to a new country** | My first time coming to a place alone | Interview # 7 |
| **Agency** | **Personal Agency, seeking studies or employment** | , but at the same time, once you get a chance to, for example when the child grows up, they can go to daycare or school, you have can go to work or study, or somewhere , | Interview # 7 |
| **Agency** | **Personal Autonomy/Independence** | alone, I need a family or someone with me, but here I am independent, I can make it by my own, I don’t need any help, like family, or brother, you can do it by your own ,as long as you want that thing, that is itself a challenge for me. | Interview # 7 |
| **Agency** | **Positive experiences, recieving country, more freedoms** | but here in Canada, the good thing is that, nothing is hard, you can make it, as long as you want it, here everything is open, like available for you, you just have to work for it, its not like a complicated country, | Interview # 7 |
| **Always There** | **Being available** | Yes, so any questions I have, I can ask (Midwife ), | Interview # 7 |
| **Appreciative of Freedom** | **Acculturation, difference in values** | culture tells the mothers to be at home and the family life is really important but here, they say to go outside, and socialize and things… | Interview # 7 |
| **Appreciative of Freedom** | **Acculturation, reverse effect, motivation to leave, more freedom** | . I think here actually is more, I like it, the culture, the culture helps you to be, in a good way. | Interview # 7 |
| **Appreciative of Freedom** | **Acculturation, reverse effect, motivation to leave, more freedom** | pregnant woman should stay at home, pregnant women should not go out, many stuffs are there, normal things, but here, | Interview # 7 |
| **Appreciative of Freedom** | **Embracing new values, maintaining traditional ones too, forming new identity** | but you are enjoying being here and going out, or do you hold on to those values to be at home with the baby? Or both? | Interview # 7 |
| **Busy-ness** | **Keeping busy, navigating new environments** | you have many things to do being new, | Interview # 7 |
| **Children as Purpose** | **Children as Purpose, motivation to leave country behind, change, give oppurtunity** | I want to stay here, for me and my child, I don’t want my child to have the same life or same future, so I decided to come to Canada. | Interview # 7 |
| **Children as Purpose** | **Children as reason, being a mother as meaningful** | you feel like you in something in your life, as a Mother, you give your whole life to your child. I see it as a good thing. | Interview # 7 |
| **Children as Purpose** | **Children as reason, spending time raising them** | you have to be with your children of course , | Interview # 7 |
| **Culture and Environmental Shocks** | **Acculturative Stress – Weather changes** | have to get a real coat, I have never been to, here, the cold is zero degrees. Our place, it is 20, our place, it is cold!  ( | Interview # 7 |
| **Culture and Environmental Shocks** | **Acculturative Stress? Differences in food** | was in the shelter, we weren’t eating what we wanted, I wasn’t, it was from the cafeteria, it was okay. But the bad thing, you can’t eat the traditional food, it is more what is there. | Interview # 7 |
| **Family Seperation** | **Seperation of Family** | No, I don’t have any family or know anybody here, I don’t have. | Interview # 7 |
| **Feels Like Home** | **Building Safe Space** | It is a really simple and nice place, the people, midwife, doctors, all the social workers, they help you from the bottom of their heart, they do their best to make you satisfied, it’s a really nice place, and I am lucky to be here at MB, they help me a lot. | Interview # 7 |
| **Feels Like Home** | **Building Safe Space/Gratitude** | MB is a really nice place, and so.. and there is nothing else I can say. | Interview # 7 |
| **Friends** | **Peer Support, living with friend** | And this roommate, she is a friend? I met here actually…at YMCA. And when you left, you said to get an apartment together? 07: Yes, and we cook together, with | Interview # 7 |
| **Having Help and Support** | **Access to Social Assistance, income support** | – And once social assistance is set up , | Interview # 7 |
| **Having Help and Support** | **Other government assistance : PRAIDA Shelter** | TA – When you first came, they gave you shelter as well? 07: Yes, I stayed there for about 1 month and 2 weeks. | Interview # 7 |
| **Having Help and Support** | **Other government assistance : PRAIDA Shelter** | , the government were helping me a lot. | Interview # 7 |
| **Having Help and Support** | **Referral to LMB, from Community** | Yes. The nurse that works there, she told me to come here. | Interview # 7 |
| **Helps with All** | **Informational Support** | and the midwife and doctor are following me, and giving me information | Interview # 7 |
| **Helps with All** | **Informational Support, preparing for birth** | o get ready for the baby, or they give me information about what I have to do, how to go to the hospital, I should first register myself and get a card, prepare for my child, stock clothes, they help me with many stuffs, I wouldn’t do it by my own. | Interview # 7 |
| **Helps with All** | **Informational Support/Holistic Care, getting ready for birth** | She helped me with the birth stuff, where to go, what I have to do, how to prepare, the certificate, how to get a passport for the baby, and many stuffs like that she gave me information, that I know now, so I can do it on my own. | Interview # 7 |
| **Helps with All** | **Instrumental support, access to healthcare workers** | : Yes, they have helped me find a doctor at the hospital | Interview # 7 |
| **Helps with All** | **Instrumental Support, giving clothing** | Yes, they have helped me, they have given me clothes. And they are still helping me as well. | Interview # 7 |
| **Helps with All** | **Instrumental, pre-natal check ups** | , they make a meeting every week, for pregnant women . | Interview # 7 |
| **Helps with All** | **MB Informational support, ease with navigating** | information through websites, through the social worker, | Interview # 7 |
| **Helps with All** | **MB inforrmational support, about healthy eating behaviours** | Yea, these things, they advice you here, give you information that you don’t know, especially, when I am in this position, they tell you to do this and that, better to eat this food, avoid this, sleep well, they tell you many stuffs. | Interview # 7 |
| **Immigration Process** | **Traumatic experiences?** | because I came as Refugee, | Interview # 7 |
| **Immigration Process** | **Traumatic Experiences? Motivations to Leave** | Saudi Arabia, wasn’t born there but raised for 24 years, what made me leave that country, was I couldn’t find want I wanted in the future what I want, I lived there for 24 years, but I could not continue my education, could not work, no freedom for me, everything was complicated, it is a difficult country, to live how you want, since I have been married and moved to other Arab countries, it was almost the same, | Interview # 7 |
| **Immigration Process** | **Traumatic Experiences? Motivations to Leave** | and also if you want to drive, they just allowed females to drive to the car, to work in the shop it, anything, was not easy, the society were not helping you to do whatever you want. | Interview # 7 |
| **Immigration Process** | **Traumatic Experiences? Reasons to Leave** | My home, if I was talking about SA, it would be worse. I don’t see SA as my home-country. I don’t like that country. | Interview # 7 |
| **Isolation** | **Isolation, differences in community from home-country to here** | Yes, you feel a bit isolated, compared to before | Interview # 7 |
| **Isolation** | **Reduced Social Support/Isolation** | it is hard when you are alone, | Interview # 7 |
| **LMB Recommendation** | **MB recommendation** | Yes, they need to be bigger, LMB (laughing). | Interview # 7 |
| **Maintaining Traditions and Language** | **Cultural Maintenance, cooking home dishes** | but now I can cook, the traditional things, If you need to get the recipe, get it from youtube or the family…my mom, she tells me. | Interview # 7 |
| **Maintaining Traditions and Language** | **Cultural Maintenance, cooking home dishes** | a powder, it is very good for your health and everything, you just need to cook with the onion, tomato and garlic and with hot water, you can eat it with bread, and we can find this in the shops. You put salad on it…And we can make any soup, chicken soup. | Interview # 7 |
| **Positive Attitude** | **Maintaining Health, healthy behaviours** | sleep well, relaxed, positive, eat well, exercise, there are many things to avoid, | Interview # 7 |
| **Positive Attitude** | **Positive Experiences Recieving Country, nice, friendly people** | people here, that can show you around. | Interview # 7 |
| **Positive Attitude** | **Positive experiences with Recieving Country** | really nice country, nice place, nice people here too, | Interview # 7 |
| **Positive Attitude** | **Positive experiences with Recieving Country** | Even coming today, I was a bit lost, one lady was following me the whole time, and I asked her if she knew where LMB was, she is like, trying to help , | Interview # 7 |
| **Pregnancy & Parenting Stresses** | **Difficulties with being pregnant** | I was pregnant, for 7 months and it was kind of difficult for me at first, | Interview # 7 |
| **Pregnancy & Parenting Stresses** | **Pregnancy difficulties, fatgiue** | actually, as a pregnant woman yes, you feel tired, | Interview # 7 |
| **Support and Love** | **Partner Support** | Yes, always talk with my husband , | Interview # 7 |
| **Transnational Connections** | **Maintaining transnational relationships, speaking to family daily** | and my mom, through the phone of course, that makes me feel, like they give me more support | Interview # 7 |
| **Transnational Connections** | **Source of strength, family communication** | it is really nice, to find the support from the people you really know. | Interview # 7 |

| **Categories** | **Code** | **Excerpt** | **Source** |
| --- | --- | --- | --- |
| **Agency** | **Partner support to seek opportunities in Canada, integrate into community to adapt to new life** | Interviewer asked her about if she felt any differences in Freedoms, from Pakistan to coming to Canada. She explained that her husband has always been willing to give her freedoms. He says “do whatever you like”, go to the shopping center, go to classes, whatever you like, enjoy your life. Even in Pakistan, same thing, go and see your family. | Interview # 8 |
| **Agency** | **Learning new languages, part of settlement process/integration into society** | .  She also mentioned that she wants to start learning French in the near future | Interview # 8 |
| **Agency** | **Learning new languages, part of settlement process/integration into society** | She explained that her English has improved a lot since she has been here. | Interview # 8 |
| **Agency** | **Learning new languages, empowerment and building human capital for settlement and integration** | She said she would like to have some French classes. | Interview # 8 |
| **Agency** | **Relationship Building, meeting other mothers** | She also brings her kids to school and talks and meets with the other Parents . | Interview # 8 |
| **Agency** | **Learning new languages, empowerment and building human capital for settlement and integration** | She said she would like to have some French classes. | Interview # 8 |
| **Appreciative of Freedom** | **Feeling fortunate, gratitude about coming to Canada** | She was happy to come to Canada , | Interview # 8 |
| **Appreciative of Freedom** | **Partner support to seek opportunities in Canada, integrate into community to adapt to new life (family Involvement)** | She explained that her husband has always been willing to give her freedoms. He says “do whatever you like”, go to the shopping center, go to classes, whatever you like, enjoy your life. Even in Pakistan, same thing, go and see your family. | Interview # 8 |
| **Children as Purpose** | **Children as source of support for parents, for parental responsibilities, day-to-day living** | – I asked about her boys, and she said sometimes they help. | Interview # 8 |
| **Connects Women** | **MB as a milieu for fostering relationships between parents** | She said she also met friends through MB. About 10 to 15 friends | Interview # 8 |
| **Connects Women** | **MB groups as milieu for building relationships with other parents** | She also mentioned the groups (pre-natal groups) have allowed her to make some friends too. | Interview # 8 |
| **Connects Women** | **Building and maintaining friendships as source of strength (within similar ethnic community)** | She also explained that what helps her be happy is the friends she has made. Meeting  them | Interview # 8 |
| **Connects Women** | **Environment + Space (Sharing Stories, Meeting Others, Making Friendships)** | She said she also met friends through MB. About 10 to 15 friends . | Interview # 8 |
| **Connects Women** | **MB groups as milieu for building relationships with other parents** | from the groups with *** to the meetings with the nurse and doctor.  She also mentioned the groups (pre-natal groups) have allowed her to make some friends too. | Interview # 8 |
| **Culture and Environmental Shocks** | **Exposure to and disliking parts of Canadian culture (food)** | The food provided in the schools, they did not like. It was mostly macaroni and cheese, chicken, s o | Interview # 8 |
| **Culture and Environmental Shocks** | **Fear related to unfamiliar systems in new country (transportation)** | .  She said at first, she was scared to use it, she said she was scared of the escalator too! | Interview # 8 |
| **Family Seperation** | **Leaving family in home-country, difficulties with separation** | ) – I asked her about her family in Pakistan, who was there.She explained that her mother is there. – I asked her if the separation was difficult or not.She said yes, it is hard, but going okay now.  She | Interview # 8 |
| **Family Seperation** | **Distant family relationships, difficult when family member sick, or in event of death** | She also shared that her Father passed away to 2 years ago.She replied saying, yes that it was hard, but that she is better now. | Interview # 8 |
| **Family Seperation** | **Separation of Family** | She explained that her mother is there. | Interview # 8 |
| **Family Seperation** | **Separation of Family** | She also shared that her Father passed away to 2 years ago. | Interview # 8 |
| **Family Seperation** | **Separation of family** | She said that she had 2 brothers, and 1 sister, her sister is married and has 4 children. Her brothers are studying . | Interview # 8 |
| **Feels like Home** | **LMB as safe and familiar space for clients** | ,  but she said she likes it here (at MB). | Interview # 8 |
| **Feels like Home** | **Building (+Maintenance of) Trustworthy Relationships/Accompainement/Being Available** | she likes to spend time with the staff members too. | Interview # 8 |
| **Friends** | **Community-environment as resource for building relationships with other parents** | them through the parcs. She also brings her kids to school and talks and meets with the other Parents. The Parc-Extension area has many Pakistani families. She said she has made about 4 friends in the area. | Interview # 8 |
| **Friends** | **Building Relationships, efforts to make friends** | She explained that would go out and try to make friends too. | Interview # 8 |
| **Friends** | **Peer Support, friendships made source of happiness** | She also explained that what helps her be happy is the friends she has made. | Interview # 8 |
| **Helps with All** | **Positive outcome with health-care individual intervention of vaccination for family** | She said when she first came to LMB, they updated her and her children’s vaccinations, which was good. | Interview # 8 |
| **Helps with All** | **MB strategy to build knowledge and skills for navigating unfamiliar systems (the transportation system)** | She also said that the Educator also showed her the metro system. Her and another client were taken down to the metro system and explained how it works. | Interview # 8 |
| **Helps with All** | **Instrumental Support** | receiving good care from LMB. | Interview # 8 |
| **Helps with All** | **Holistic Care (Interdisciplinary Approach to Care, Personalized** | , and she maintains health by coming to LMB. | Interview # 8 |
| **Isolation** | **Isolation** | When she first arrived, she said she was very shy, because of her inability to speak English. | Interview # 8 |
| **Isolation** | **Separation of Family** | She explained that she did not have any extended-family when she arrived here, | Interview # 8 |
| **Language Barriers** | **Language barriers, not fluent in English, difficulties expressing self** | There were language barriers with this participant. Her English is about intermediate level, she can mostly understand, but has some difficulties expressing herself. I believe this impacted on the quality of her responses and her ability to answer the questions or go more into depth. | Interview # 8 |
| **Language Barriers** | **Language barriers, not fluent in English, difficulties expressing self** | When she first arrived, she said she was very shy, because of her inability to speak English. | Interview # 8 |
| **Language Barriers** | **Language Barriers** | But at first, she was scared and shy of speaking in English, as she would often make mistakes. | Interview # 8 |
| **LMB Outcome** | **Reflection of positive feedback of MB; referring others to the organization** | She said she also referred some of her friends to MB. | Interview # 8 |
| **LMB Outcome** | **Expressed gratitude for LMB work and team** | She said she appreciates MB, | Interview # 8 |
| **Maintaining Traditions and Language** | **Maintaining culture, maintained through home-country dishes** | So she would make their lunches for them to bring to school. She would cook more Pakistani dishes. | Interview # 8 |
| **Maintaining Traditions and Language** | **Maintaining religion and religious-traditions, importance of transmitting religion to children** | From Pakistan, she said some traditions they maintain her are celebrating the Ramadan, with the fasting that happens in the Spring. She said the children do not participate, only when they are old than 12 years old, so it is just her and her husband. | Interview # 8 |
| **Maintaining Traditions and Language** | **Cultural Maintenance – Cooking dishes from home** | She would cook more Pakistani dishes . | Interview # 8 |
| **Maintaining Traditions and Language** | **Maintaining Transnational Relationships** | She said she also speaks to them with Skype or the phone. But there are sometimes internet problems over there. | Interview # 8 |
| **Navigation of System** | **For services not offered by MB, outside referrals (bridging) to other community resources to address need** | She said she has found some elsewhere, also the Educator helped her find some at the library, | Interview # 8 |
| **Navigation of System** | **For services not offered by MB, outside referrals (bridging) to other community resources to address need** | She said she has found some elsewhere, also the Educator helped her find some at the library, | Interview # 8 |
| **Positive Attitude** | **Positive Attitude** | Smiling often | Interview # 8 |
| **Religion** | **Cultural Maintenance - Religion** | She said also that she prays at home. | Interview # 8 |
| **Support and Love** | **Family closeness as source of strength during settlement** | She explained that she did not have any extended-family when she arrived here, but she was with her husband and children. Because of family being here (Children and husband), it was not difficult. | Interview # 8 |
| **Support and Love** | **Presence of marriage partner as source of strength** | , so what has helped you settle here has been your loving husband | Interview # 8 |
| **Support and Love** | **Children as source of support for parents, for parental responsibilities, day-to-day living** | also her daughter has helped her too, she said her daughter is very intelligent, and she would help her with changing the baby diapers.She explains that she helps with cleaning, but not really cooking yet. | Interview # 8 |
| **Support and Love** | **Family closeness and spending time together as source of strength** | She explained that they eat together, the husband goes to work, the kids are doing well, sleeping well, she cooks for the entire family . | Interview # 8 |
| **Support and Love** | **Family closeness as source of strength during settlement** | Because of family being here (Children and husband), it was not difficult. | Interview # 8 |
| **Support and Love** | **Family Involvement, Children as source of support** | She also explains that having her children here, is a good thing. | Interview # 8 |
| **Support and Love** | **Family Involvement, Partner support and presence** | She said her husband helped her a lot,  a | Interview # 8 |
| **The Park** | **Building relationships within ethnic community** | She found friends in the park that were also Pakistani and would speak Pashtu. She would simply go up to people and say “hello/hi” in Pashtu to see if they would understand her. | Interview # 8 |
| **Transnational Connections** | **Maintaining distant-family community as a source of strength and coping with separation** | She said it is hard to communicate because of the time differences. She explained that they speak often, every two days, using the phone. | Interview # 8 |
| **Transnational Connections** | **Maintaining distant-family community as a source of strength and coping with separation** | She said that she had 2 brothers, and 1 sister, her sister is married and has 4 children. Her brothers are studying. She said she also speaks to them with Skype or the phone. But there are sometimes internet problems over there. | Interview # 8 |
| **Transnational Connections** | **Maintaining Transnational Relationships** | She said it is hard to communicate because of the time differences. She explained that they speak often, every two days, using the phone  . | Interview # 8 |

| **Categories** | **Codes** | **Excerpt** | **Source** |
| --- | --- | --- | --- |
| **Agency** | **Personal Agency?** | Yes, I like it. But I told you, no I am on a diet, I want to lose weight, these days , | Interview #9 |
| **Agency** | **Personal Autonomy** | On our own, we go to outside, we meet the people , | Interview #9 |
| **Agency** | **Wilingness to Learn** | No, not French. I want to learn, but … | Interview #9 |
| **Agency** | **Willingess to Learn** | , but evening classes, for the 3 months, we took evening classes, because day classes were finished. | Interview #9 |
| **Agency** | **Willingness to Learn** | then I went to school, for 2-3 months, I developed myself. And for my other family, it was the same thing. | Interview #9 |
| **Agency** | **Acculturative Stress (Weather, Differences, Unfamiliarities with New Environment** | , where to find a good school, then we went to the school | Interview #9 |
| **Agency** | **Personal Autonomy, going outside** | On our own, we go to outside, we meet the people  , | Interview #9 |
| **Agency** | **Maintaining Health, Self-Care** | , oh Im sad, then I go outside, when I like, take the fresh air, then my brain is very fresh, and it helps me a lot. | Interview #9 |
| **Always There** | **Building Safe Space** | so my doctor was here with me, when I have some problems, I call her, and she helps me, | Interview #9 |
| **Always There** | **Creatng Safe Space/Being Available** | When we have some problems, I call here, and right away they help me, when I had my baby, she had some problem, I would just call here, | Interview #9 |
| **Always There** | **Holistic Care** | the Social worker, I just met her one time, but If I need, I will meet with her. | Interview #9 |
| **Always There** | **Creating Safe Space- Available** | Because when I have some problem, and they help me | Interview #9 |
| **Always There** | **Building Safe Space** | when I have some problems, I call her, and she helps me, | Interview #9 |
| **Always There** | **Creating Safe Space=being Available** | 09: Because when I have some problem, and they help me. | Interview #9 |
| **Appreciative of Freedom** | **Acculturative Stress - Opposite** | Yes, more freedom, What we want we can do.. but there, we cant do, because of the people, they are talking behind you, then we get tired, someone is talking behind me, you don’t want this, so here, no one is talking behind you, you can what you want, more freedom, and also, like more safe… | Interview #9 |
| **Appreciative of Freedom** | **Positive experiences in Canada** | My mom always said, I like Canada, here, you can do what you want, you can follow your religion, and they don’t force you to do this, what you want, you can do everything here, this is the best thing. | Interview #9 |
| **Appreciative of Freedom** | **Positive Experiences in Canada** | Yes, the best things is more safety.. | Interview #9 |
| **Appreciative of Freedom** | **Positive Experiences in Canada** | Go to work, you are safe here, you can go alone. Everywhere, you can go alone, everywhere . | Interview #9 |
| **Appreciative of Freedom** | **Positive Experiences with Recieving Population** | and we don’t know the place, then we ask the people, all the people are very nice. | Interview #9 |
| **Appreciative of Freedom** | **Acculturative Stress - Opposite** | my country you cant go to work easily, when you go to outside, you have to pass many challenges. People will say, why do you go to work, she has a husband, but here, it is more easy for us. | Interview #9 |
| **Appreciative of Freedom** | **Traumatic Experiences?** | There is like robbers, for your children too, there are many robbers there | Interview #9 |
| **Appreciative of Freedom** | **Positive Experiences in Canada** | Go to work, you are safe here, you can go alone. Everywhere, you can go alone, everywhere . | Interview #9 |
| **Appreciative of Freedom** | **Positive Experiences with Receiving Country + Population (Sense of Belonging, Gratitude)** | 09: No, my father said, here, it is very safe place, you can go outside, alone. | Interview #9 |
| **Busy-ness** | **Acculturative Stress—Unfamiliar with new Environment** | The first time when we came here, finding schools, for English, this was difficult, and also a doctor too. | Interview #9 |
| **Culture and Environmental Shocks** | **Acculturative Stress** | When I came first? The first thing was the weather, ahh.. oh my god, in my country, there is not a lot of snow, the weather was hard for us, | Interview #9 |
| **Culture and Environmental Shocks** | **Acculturative Stress (Weather, Differences, Unfamiliarities with New Environment** | TA- And when you first tried, you didn’t know where you were going? | Interview #9 |
| **Friends** | **Peer Support** | we had friends, | Interview #9 |
| **Friends** | **Peer Support** | you have a party, sometimes we just invite friends | Interview #9 |
| **Friends** | **Peer Support/Referral To LMB** | Just my friend said to go there , | Interview #9 |
| **Friends** | **Peer Support** | I phone call my friend | Interview #9 |
| **Friends** | **Family Involvement, relatives providing help** | I phone call my friend,  sometimes my relatives, they will help me. | Interview #9 |
| **Friends** | **Peer Support** | Yes, yes, like my close friends. | Interview #9 |
| **Gives Hope** | **Emotional Support** | Yes, the first week, I called everyday and I didn’t know anything. I called the nurse and she explained everything; “don’t worry – don’t worry”, its fine, its fine | Interview #9 |
| **Having Help and Support** | **Access to CLSC Services, negative** | But there, 2 hour wait, outside the gate. It starts at 6am, then I think at 8am, it opens . | Interview #9 |
| **Having Help and Support** | **Access to Health Care Services** | now my mom, and brothers and sisters they have a doctor. | Interview #9 |
| **Having Help and Support** | **Other Government Assistance** | : MY one sister, two sister and one brother, they were under 18, they had some tests, and they went to French school, | Interview #9 |
| **Helps with All** | **Creating Safe Space** | But here, it is good, doctor is good, nurse, everyone is very nice, very nice people, very helpful | Interview #9 |
| **Helps with All** | **Gratitude** | everything at LMB, is very good, doctors…. I found that, my experience is very good here, and we have like, we find, very good services, the doctors, the nurse. | Interview #9 |
| **Language Barriers** | **Language Barriers** | the second thing was the language, I understood English but it was a problem , | Interview #9 |
| **Language Barriers** | **Language Barriers** | Ya, the first time when I came, I was like oh-my-god, when the people were speaking, I understood, but not much, but now I understand everything. | Interview #9 |
| **Language Barriers** | **Language Barriers** | No, I didn’t go there.. because 2-3 times I went there, to Saint-Justine, I had too much problems, difficulties… | Interview #9 |
| **LMB Limitation** | **MB Context – High patient Volume** | No, no. I thought, if its like this, we have some problems, and they say come, I want it to be like this, but its not. To see the doctor and see the nurse, we cant see them at the same time, we have to go take the appointment. Last week, I took an appointment and got it today. | Interview #9 |
| **LMB Limitation** | **MB context – High Patient Volume** | For me its the doctor, when she is sick, I have to go to the CLSC or Emergency.. | Interview #9 |
| **LMB LImitation** | **MB Recommendation/Limitations** | …. The one thing, when I, take appointment, it is very long, its like a month. My question is, when we need appointment, we take when we have problems, if we can get earlier, it is good for us. | Interview #9 |
| **LMB Limitation** | **Wanting to Remain as Client** | TA – And you plan to stay to here with them, until she is a bit older.09: Yes. Yes | Interview #9 |
| **LMB Recommendation** | **MB Recommendation** | Just, I want here, to be, if we call, doctor is available, when I have problems or when my daughter has problems, I want okay, doctor is here, you can come. | Interview #9 |
| **Maintaining Traditions and Language** | **Cultural Maintenance** | Yes, all the things we had in Pakistan, we will do here too, like special occasion, we do the same thing, like Pakistan, like religion, friends, same thing like Pakistan. | Interview #9 |
| **Religion** | **Cultural Maintenance** | Ya, the, I think, dates, if you eat one date in the morning, on a empty stomach, it will help you all day, don’t eat anything only one date. When we have Ramadan, we wake up for eating, at 3’oclock, then we forced, we drink, glass of water, and take, 1-2 dates, then we eat something, so all day, didn’t eat, it is good for us, and we are not hungry or thirsty. | Interview #9 |
| **Maintaining Traditions and Language** | **Cultural Maintenance (Religion, Values, Language, Traditions)** | 09: Eating…. After Ramadan. When having babies, newborns babies have parties, we invite friends and families. | Interview #9 |
| **Navigation of System** | **Continuity of Care** | Yes, fever..and also, too much sick, and the doctor told me to go there. | Interview #9 |
| **Navigation of System** | **HealthCare Services (CLSC, Hospital, Other)** | then I called to the Parc CLSC, and I took the appointment there. | Interview #9 |
| **Park** | **Relationship Building (within, across ethnic boundaries)** | TA- You meet people where? 09: Yes, in the park.  Info | Interview #9 |
| **Religion** | **Cultural Maintenance, Religion** | Yes, I follow the religion here. | Interview #9 |
| **Support and Love** | **Family Involvement** | because my father was here for a long time, when we have some problem, he help us, because that’s why we didn’t have.. | Interview #9 |
| **Support and Love** | **Family Involvement** | I am very happy here, because my whole family is here, my mom, my brother, my sister. We are very happy, because we are living in front of each other. | Interview #9 |
| **Support and Love** | **Family Involvement** | Yes, my father was here, that’s why we didn’t have problems, we had some, but not many. | Interview #9 |
| **Support and Love** | **Family Involvement** | when we finish everything, we, my sister, we go to my moms, we are together, we sit together, we talk to each other, for 1-2 hours, at my moms home, sisters, brothers, I think this is the best thing. | Interview #9 |
| **Support and Love** | **Family Involvement** | , just to get together and eat together, and talk to each other. It is going to be healthy too for us. Mentally. | Interview #9 |
| **Support and Love** | **Family Involvement** | TA – Is that part of the reason why you wanted to come? Yes, and he sponsored my whole family, my mom, my brother, sisters. | Interview #9 |
| **Support and Love** | **Family Involvement, family living close in proximity** | TA – And everyone is still here? And you live close to each other. 09: Yes, close to each other. | Interview #9 |
| **Support and Love** | **Family Involvement, spending time together, at end of day** | 09: Sometimes, when we finish work, and our homework, clean… and cooking.. then we go together, my mom’s home. | Interview #9 |
| **Support and Love** | **Family Involvement, presence of many relatives in Cnada** | we have many relatives, | Interview #9 |
| **TIME - ADAPTED** | **Time – More Adjusted** | When I was pregnant, I had, 3-4 years I was in Canada , so I was used to it. | Interview #9 |
| **TIME - ADAPTED** | **Time-Adjustment, Navigating** | 09: Yea, after 2-3 months, we like, we used to everything, | Interview #9 |
| **TIME - ADAPTED** | **Time- Language Development, Building Capital** | English has gotten easier…. Like practicing, using it more, the language | Interview #9 |
| **Varied Team** | **Holistic Care** | she told everything about LMB, you have your own doctor, nurse, | Interview #9 |
| **Varied Team** | **Holistic Care** | she told everything about LMB, you have your own doctor, nurse,  and everything she explained to me, | Interview #9 |

**Notes**

| **Categories** | **Code** | **Excerpt** | **Source** |
| --- | --- | --- | --- |
| **Active Participation** | **LMB Goal to ensure Parents are partners in group intervention, learning and taking lessons learned home** | The mother’s are familiar with each other. From attending the group previously, three times before, I have seen the mothers come to the group prior. | Group 1 |
| **Active Participation** | **Strength: Motivation to communicate and learn about similar parent’s experiences** | o   To supervise their children, to provide discipline as needed, to participate in the activities and help facilitate the activities, translate for their children as needed to increase their understanding of instructions. | Group 1 |
| **Active Participation** | **LMB goal to teach parents about proper child behaviours through explanation during groups** | The mother’s would help provide instruction to their children, once *** would explain it, they would repeat in their mother-tongue throughout the activity to ensure their children understood the instructions. | Group 1 |
| **Agency** | **Cultural value of raising children independently (non-use of daycare services)** | she explained the mother’s decision to not put her son in daycare was more cultural reasons, | Group 1 |
| **Child Development Issues** | **Migrant-status related barrier to accessing daycare services for children** | these families were selected to be part of this group as the children have minimal stimulation as they do not go to daycare. | Group 1 |
| **Child Development Issues** | **Consequence of reduced access to service can be lead to behavioural concerns and missing out on learning activities for children** | *** explained that if children are not properly socialized, there can be problems when they enter into school (Kindergarten or Primary School). She said these environments are very structured and if there are behavioural issues (for example, any aggression, hitting, not sharing, not listening to instruction or the adults), the child will end up being disciplined and may miss out on learning activities. | Group 1 |
| **Child Development Issues** | **LMB sharing instruction for how parents can properly discipline their child based on certain behaviour** | For when the children’s behaviour was not appropriate, not sharing or waiting their turn, *** did instruct them to hold onto their children when the game continued to make them wait. Also, *** provided some instruction on games that could help with learning behavioural skills (waiting turns; bean bag game). | Group 1 |
| **Child Development Issues** | **LMB intervention for childhood stimulation for migrant families not accessing them through the social system** | *** also explained that there is a stigma attached for children who do not go to daycare. She said that children may have difficulties making friends and may not be as ‘socialable’ as the other children. | Group 1 |
| **Children as Purpose** | **Entire purpose of the group** | Observation. | Group 1 |
| **Connects Women** | **Resource: Other parents participating in group, similar situation (recent migrant, young children, same borough)** | For example; “Yes, I have one more child, I have 3 daughters” “We are Muslim, not Hindi”. Also, # 12 shared with her that her “English was not great, and was learning”. | Group 1 |
| **Connects Women** | **LMB intervention for childhood stimulation for migrant families not accessing them through the social system** | Although not said, a resource itself is the weekly group that provides the children with an opportunity to play with other children, learn rules and listen to both French and English. | Group 1 |
| **Connects Women** | **Language barrier as challenge to communicating with other group participants** | The children are also familiar with each other. | Group 1 |
| **Feels Like Home** | **LMB goal to create long-term relationships with and between participants for group activities** | Equipment used include the floor mat on the ground, games were played on the mat, other activities were also played at the small table, including snack time. Other equipment used included; playdough, crayons, toy cars, bowling pins, bean bag game. What was also used was a stereo to play a song at the beginning. | Group 1 |
| **Feels Like Home** | **LMB environment seen as safe space, shown by comfort to breastfeed during group session** | 12 is now breastfeeding her baby during the game. | Group 1 |
| **Feels Like Home** | **MB seen as a Safe Space (Mothers show comfort in space, taking off headscarf, breastfeeding, letting children play freely)** | Observation. | Group 1 |
| **Gives Hope** | **LMB reinforcing to parent healthy selection of snack for child** | *** to 12; “that is a good snack, it is healthy!” (yogurt and apple). | Group 1 |
| **Helps with All** | **Resource: LMB staff as resource for information for parents concerning community (available schools, safety of borough)** | 05 asked some questions during the group about her moving. She is planning to move to Hochelaga and asked *** about the area and the schools as well. She also asked if the area was safe . | Group 1 |
| **Helps with All** | **LMB sharing with parents rationale for selecting activities** | *** “Okay another few minutes of free play”. The children grab different toys and bring them to the table. *** to parents “I like to see what they choose during free play – How they play” “The boys are playing with blocks – and the girl is playing with the stuffed animals”. | Group 1 |
| **Helps with All** | **MB Staff as Resource for information and for advice (Schools, resources in community)** | Observation. | Group 1 |
| **Helps with All** | **Mother’s show interest in learning about resources in their environment (Schools).** | Observation. | Group 1 |
| **Helps with All** | **Resource: LMB offering French-language books to facilitate francization of children and parents** | Also, from previous group participation, *** also does offer the mothers with French reading books for the children. She recommends bringing them home and reading them on a daily basis. This will help increase their exposure to the French language. | Group 1 |
| **Immigration Process** | **Migrant-status related financial barrier to accessing daycare services for children** | For participant 11 and 12, *** explained that due to their migrant statuses, there are restrictions to accessing daycare services that are full-time low-cost/subsidized by the government. They are unable to pay for private services. Therefore, both mothers are waiting to place them in daycare pending their statuses. | Group 1 |
| **Language Barriers** | **Resource: Other parents participating in group, similar situation (recent migrant, young children, same borough)** | There are language barriers that exist between the mothers making verbal communication difficult. For instance, 11 speaks Tamal and some English, 12 speaks limited English and Bengali and 05 speaks French (quite fluently). | Group 1 |
| **Models Behaviour** | **Parents supportive and learning from discipline given to children for unacceptable behaviour** | ·         Tone of voice also became firm, when *** disciplined any of the children. A firm “no”, with her finger symbolling as well was done a few times. | Group 1 |
| **Models Behaviour** | **LMB approach towards participants with language barriers, non-verbal communication to facilitate understanding** | ·         The parents seemed supportive of ***’s disciplining, at times they reinforced her message (after child goes to parent, they would discipline in mother tongue as well), or they would simply watch ***, but did not appear concerned, alarmed or it was wrongly-deserved. | Group 1 |
| **Models Behaviour** | **Offering positive reinforcement for ‘good’ child behaviour** | *** “Thank you”, while showing were to put them. “Good job!” | Group 1 |
| **Models Behaviour** | **Parents supportive and learning from discipline given to children for unacceptable behaviour** | *** had to hold back 11 son from throwing out of turn. He was also unwilling to give the bean bags to 12 daughter at her turn. *** held him and counted “1-2-3” “Give”. He did not, so *** took them out of his hand. 11 holds him. | Group 1 |
| **Models Behaviour** | **Parents supportive and learning from discipline given to children for unacceptable behaviour** | 11 tried to take the roller from *** “No-No-No” he finally stops. Mom does not intervene yet is watching interaction. | Group 1 |
| **Models Behaviour** | **Parental involvement in group activities and reinforcing positive behaviour of children** | the mothers and also between *** and the mothers and children. For explaining games for example, she would gesture what to do (for example, throw a ball would be imitated). | Group 1 |
| **Models Behaviour** | **LMB goal to teach parents about proper child behaviours through explanation during groups** | *** “Next game is Bean Bag Toss”, she pulls out the game and explains to the Mothers that it is a good game for teaching them to take turns. *** shows how to play, demonstrates throwing. “We wait our turn” “Throw 3 bags” 11 Mother explains to her son in Tamal what to do (loudly) 11+12 watching her children play and are smiling. | Group 1 |
| **Models Behaviour** | **LMB teaching children and parents about personal hygiene (hand washing)** | *** “We are finished – now we are going to have a snack!” “Lets go wash our hands” | Group 1 |
| **Models Behaviour** | **Demonstrating positive behaviours with children (saying thank you when receiving something)** | We say Thank you”, she repeats until they say. | Group 1 |
| **Pregnancy & Parenting Stresses** | **Challenge; Parental concern of moving children to another borough, concerns related to changing school and friends** | From speaking to this participant before, she has also expressed her challenge of moving her children away from their schools and friends. | Group 1 |
| **Protection of Rights** | **Parents supported to translate and teach in mother-tongue to enhance child’s understanding of activity** | *** “Next activity is Mom and child massage – to say body parts while massaging”, “It can be in your own language if you want”. *** has 12’s daughter to demonstrate. *** takes small toy cars and rolls them over her arms and legs saying the body parts in English. She turns to 12, “What is arm in Bengali?”. | Group 1 |

| **Category** | **Codes** | **LM Code** | | | | | **Excerpt** | | **Source** | | | |
| --- | --- | --- | --- | --- | --- | --- | --- | --- | --- | --- | --- | --- |
| **Agency** | **Overcoming Barriers, Building Capital (Knowledge + Skills, New Languages)** | **interested to learn/have informaiton** | listening very attentively | | | | | | | | | Group 2 |
| **Agency** | **Overcoming Barriers, Building Capital (Knowledge + Skills, New Languages)** | **eager to learn** | | | What other questions are there?” I feel that she does not want to miss any information. | | | | | | Group 2 | |
| **Agency** | **Overcoming Barriers, Building Capital (Knowledge + Skills, New Languages)** | **eager to learn, seeking information** | | | | but maybe you can show me a few games now that I can play with my baby?” | | | | | Group 2 | |
| **Always There** | **Building (+Maintenance of) Relationships/Accompainement/Being Available** | **giving time** | | | willing to share at this time with her ) | | | | | Group 2 | | |
| **Always There** | **Social Climate (Safe Space, Respect, Warm, Welcoming, Adequate Equipment)** | **Giving time?** | | this group went a little longer | | | | | | Group 2 | | |
| **Children as Purpose** | **Children (Family) as Reason/Sense of Purpose** | **children as motivation** | | | | | | the baby is worth it ” | | | Group 2 | |
| **Children as Purpose** | **Children (Family) as Reason/Sense of Purpose** | **mom baby relationship** | | | | | | it is worth it, I have a baby now ” | | | Group 2 | |
| **Connects Women** | **Environment + Space (Sharing Stories, Meeting Others, Making Friendships)** | **Encouraging sharing?** | | | | | | *** also likes for the group to share their birth experience . | | | Group 2 | |
| **Feels like Home** | **Social Climate (Safe Space, Respect, Warm, Welcoming, Adequate Equipment)** | **Informal, comfortable, warm** | | | | | | sat on the floor mats . | | | Group 2 | |
| **Feels like Home** | **Social Climate (Safe Space, Respect, Warm, Welcoming, Adequate Equipment)** | **comfortable** | | | | | | seemed comfortable | | | Group 2 | |
| **Feels like Home** | **Social Climate (Safe Space, Respect, Warm, Welcoming, Adequate Equipment)** | **Comfortable, safe** | | | | | | she begins to breastfeed her baby in the waiting room . | | | Group 2 | |
| **Feels like Home** | **Social Climate (Safe Space, Respect, Warm, Welcoming, Adequate Equipment)** | **Friendly, comfortable** | | | | | | please take your time , I | | | Group 2 | |
| **Feels like Home** | **Social Climate (Safe Space, Respect, Warm, Welcoming, Adequate Equipment)** | **Warmth,** | | | | | | lightly touches her arm . | | | Group 2 | |
| **Feels like Home** | **Social Climate (Safe Space, Respect, Warm, Welcoming, Adequate Equipment)** | **Informal, comfortable** | | | | | | baby blanket on the floor mat . | | | Group 2 | |
| **Feels like Home** | **Social Climate (Safe Space, Respect, Warm, Welcoming, Adequate Equipment)** | **Personal, friendly** | | | | | | how are you ?” | | | Group 2 | |
| **Feels like Home** | **Social Climate (Safe Space, Respect, Warm, Welcoming, Adequate Equipment)** | **smiling and laughing** | | | | | | smiles and laughs . | | | Group 2 | |
| **Feels like Home** | **Social Climate (Safe Space, Respect, Warm, Welcoming, Adequate Equipment)** | **Warmth, comfortable, informal** | | | | | | Oh – I forgot abut the tea!”  ( | | | Group 2 | |
| **Feels like Home** | **Social Climate (Safe Space, Respect, Warm, Welcoming, Adequate Equipment)** | **warmth** | | | | | | smiling, okay | | | Group 2 | |
| **Gives Hope** | **Emotional (Personal) Support (Caring, Empathy, Responsive)** | **personal** | | | | | | why are you not having fun ?” | | | Group 2 | |
| **Gives Hope** | **Emotional (Personal) Support (Caring, Empathy, Responsive)** | **Warmth,** | | | | | | MB is your family now , | | | Group 2 | |
| **Gives Hope** | **Esteem Support (Offering Hope + Self-Confidence)** | **Encouraging** | | | | | | Congratulations!” | | | Group 2 | |
| **Helps with All** | **Emotional (Personal) Support (Caring, Empathy, Responsive)** | **Support? Attentive? Listening?** | | | | | | “this topic gives me a headache”. *** jumps in, “Okay – lets talk about something else then! ” | | | Group 2 | |
| **Helps with All** | **Informational Support (Teaching, Advice, Demonstration)** | **teaching** | | | | | | explains how a fungal infection can spread; | | | Group 2 | |
| **Helps with All** | **Informational Support (Teaching, Advice, Demonstration)** | **Directive?** | | | | | | “You have to follow these steps ”. | | | Group 2 | |
| **Helps with All** | **Informational Support (Teaching, Advice, Demonstration)** | **teaching** | | | | | | discussed with you Contraception ?” | | | Group 2 | |
| **Helps with All** | **Informational Support (Teaching, Advice, Demonstration)** | **teaching** | | | | | | she will have to space out the pregnancies, with a 2^nd^ pregnancy, the due date should not be within 18 months from birth date of first – or else it is high risk” | | | Group 2 | |
| **Helps with All** | **Informational Support (Teaching, Advice, Demonstration)** | **teaching, directive** | | | | | | explains that she will need to watch better what she eats, cut out the sugars, replace the white breads and pastas with whole wheat or ‘brown’ rice or pasta or bread, she advised | | | Group 2 | |
| **Helps with All** | **Informational Support (Teaching, Advice, Demonstration)** | **teaching, directive** | | | | | | disapproving of this information. *** says “there is not bad weather, just bad clothes” | | | Group 2 | |
| **Helps with All** | **Informational Support (Teaching, Advice, Demonstration, Directive)** | **diretive** | | | | | | She repeats this three times. | | | Group 2 | |
| **Helps with All** | **Informational Support (Teaching, Advice, Demonstration, Directive)** | **directive, teaching** | | | | | | I suggest you walk everyday | | | Group 2 | |
| **Helps with All** | **Informational Support (Teaching, Advice, Demonstration, Directive)** | **directive, teaching, adapting/tailoring/responsive to needs** | | | | | | “I really suggest that for you – it is good for your mental health and for losing weight” | | | Group 2 | |
| **Helps with All** | **Instrumental + Practical Support (Interventions)** | **giving ? warmth, friendly** | | | | | | “I have something for you!” | | | Group 2 | |
| **Helps with All** | **Instrumental + Practical Support (Interventions)** | **Practical support** | | | | | | do you have any more prenatal vitamins?, she replies that is out, and *** says okay, I am going to give you some and some coupons as well (milk and eggs). | | | Group 2 | |
| **Helps with All** | **Instrumental + Practical Support (Interventions)** | **Attentive, adapted care** | | | | | | yes “that is an infection – I will write the px at the end for you” | | | Group 2 | |
| **Immigration Process** | **Discrimination (+Barriers to accessing services)** | **discrimination?** | | | | | | discrimination | | | Group 2 | |
| **Immigration Process** | **Discrimination (+Barriers to accessing services)** | **stress,** | | | | | | bill she got from giving birth . | | | Group 2 | |
| **Immigration Process** | **Discrimination (+Barriers to accessing services)** | **stress, discrimination?** | | | | | | she was not treated very well there because she does not have RAMQ, by the staff, “it was really bad”. | | | Group 2 | |
| **Immigration Process** | **Discrimination (+Barriers to accessing services)** | **Poor treatment** | | | | | | she requested a stronger pain killer than Tylenol, but that they said “No – you  are | | | Group 2 | |
| **Immigration Process** | **Emotional (Personal) Support (Caring, Empathy, Responsive)** | **empathy** | | | | | | she feels that the bill is very burdensome for new migrant families if not ridiculous for these families to pay that much). | | | Group 2 | |
| **Immigration Process** | **Immigration Process (Difficulties + Stress)** | **Migration challenges** | | | | | | immigration process  has | | | Group 2 | |
| **Immigration Process** | **Immigration Process (Difficulties + Stress)** | **stress (immigration? Motherhood? Other?)** | | | | | | a lot on her mind ” | | | Group 2 | |
| **Immigration Process** | **Traumatic Experiences** | **Tramatic experience** | | | | | | They held a gun to my head’ . | | | Group 2 | |
| **Isolation** | **Changing Family Composition (Seperation of Family)** | **No family here** | | | | | | family is not coming here , | | | Group 2 | |
| **Isolation** | **Social Support and Isolation** | **alone** | | | | | | husband is not here right now , | | | Group 2 | |
| **Knows their Clients** | **Approach; Empowerment, Encouraging Active Partnership in Care** | **adapting to women’s needs** | | | | | | So you want to lose weight” states *** “What sugary things do you like ?” | | | Group 2 | |
| **Knows their Clients** | **Approach; Empowerment, Encouraging Active Partnership in Care, Personalized** | **personal** | | | | | | says her full name to her) | | | Group 2 | |
| **Knows their Clients** | **Approach; Empowerment, Encouraging Active Partnership in Care, Personalized** | **adapting/responsive to needs** | | | | | | (anything the mothers want – she explained ) | | | Group 2 | |
| **Maintaining Traditions and Language** | **Cultural Maintenance (Religion, Values, Language, Traditions)** | **mom baby relationship** | | | | | | speaking in baby talk and in Spanish . | | | Group 2 | |
| **Models Behaviour** | **Approach; Empowerment, Encouraging Active Partnership in Care** | **Asking women what they think** | | | | | | ask her for how long she thinks she should exclusively | | | Group 2 | |
| **Models Behaviour** | **Approach; Empowerment, Encouraging Active Partnership in Care** | **Balance of teaching (clear direction) with empowering (letting women direct care)** | | | | | | mostly try to avoid “should statements” and asks what the mothers know first before doing some teaching. My feeling is that she wants to be non-judgemental but also be firm of giving them with the correct information | | | Group 2 | |
| **Models Behaviour** | **Building (+Maintenance of) Relationships/Accompainement/Being Available** | **Teaching, taking the time** | | | | | | showing | | | Group 2 | |
| **Models Behaviour** | **Esteem Support (Offering Hope + Self-Confidence)** | **encouraging, positive** | | | | | | I like the way you are with her | | | Group 2 | |
| **Models Behaviour** | **Informational Support (Teaching, Advice, Demonstration)** | **teaching** | | | | | | discussion were initiated by *** , | | | Group 2 | |
| **Positive Attitude** | **Esteem Support (Offering Hope + Self-Confidence)** | **friendly** | | | | | | and I will lose 25 lbs?!” (Excitingly, half sarcastically said) | | | Group 2 | |
| **Pregnancy & Parenting Stresses** | **Parenting Responsibilities + Concerns (+Prenatal)** | **early motherhood challenges** | | | | | | I have a lot of nipple pain . | | | Group 2 | |
| **Pregnancy & Parenting Stresses** | **Parenting Responsibilities + Concerns (+Prenatal)** | **early motherhood challenges?** | | | | | | cannot sleep, has insomnia | | | Group 2 | |

| **Category** | **Code** | **Excerpt** | **Source** |
| --- | --- | --- | --- |
| **Agency** | **Obtaining citizenship and the sense of pride with new status** | ·         she said she just obtained her Canadian citizenship last week.   *** smiling and said “congratulations!”. Everyone in the group smiled and applauded her. | Group 3 |
| **All Services in One Place** | **Internal referral to another MB personnel member to address health concern** | *** shared that LMB does offer Osteopaths for their clients, she said that outside MB, they can be about $80 per hour. “So if you are in a lot of pain, please tell me and I can try to arrange for an appointment. | Group 3 |
| **All Services in One Place** | **Internal referral to another MB personnel member to address health concern** | It doesn’t cost anything, it can be for now or during the post-partum period”. | Group 3 |
| **Always There** | **Recommending that MB be first-line for health or other concerns for clients** | But, if it is between 9-5pm, call me first and I will do some initial triage | Group 3 |
| **Always There** | **Facilitator role to answer questions, provide accurate information about healthcare interventions, managing expectations** | 35 asks; I have a question about the epidural. Can I walk after with it? *** also what is needed with an epidural is a foley catheter, because you cannot feel the need to urinate. | Group 3 |
| **Connects Women** | **Teaching strategy; encouraging peer-to-peer learning** | *** returns then says, okay lets talk about what happens at the hospital. *** asks 32 to explain the steps. | Group 3 |
| **Connects Women** | **Informing and Encouraging participation in MB activities and social events** | As she stands up, she also invites all the Mothers to the Christmas party that coming up. | Group 3 |
| **Connects Women** | **Sharing concerns related to pregnancy, seeking advice from professional** | 36 jumps in and shared that she has had a pinching pain. *** says “Yes, like an ache?” 36 replies say that she has been having the pain for 2 days, the pinching. | Group 3 |
| **Connects Women** | **Building community closeness** | *** started the group (132) by saying that many women had given birth in the last week, there were 5 births. She gave updates on each of them. When she would update them, she would ask if the participants knew anything of them. | Group 3 |
| **Connects Women** | **Sharing stories and similar experiences, maintaining humor** | ·         36 jumps in and says; yes-yes, I can never sleep on my left side. (Laughing). Saying to baby; Baby come on this side! (in a joking manner). The participants laugh. | Group 3 |
| **Connects Women** | **Teaching strategy, encouraging peer to peer teaching and learning, encouraging their own experiences as important knowledge** | *** then asks, so what medications are there for pain?32 stands up and demonstrates the positioning when the epidural is inserted. | Group 3 |
| **Connects Women** | **Relationship-building** | 35 leans forward to get some water, 32 standing up, pours some and gives it to her. | Group 3 |
| **Connects Women** | **Sharing similar stories, building relationships and rapport among participants** | 36 said she had an emergency c-section, as her heart rate was dropping (or the infant – not sure). After the delivery, she explained it was difficult, had trouble urinating. | Group 3 |
| **Connects Women** | **Positive results from letter writing and support from MB personnel for job searching** | She sat down and shared with the group that the SW had written a reference letter for her to help her with her employment searching and with the letter, she said she had some good job leads. | Group 3 |
| **Connects Women** | **Providing information about healthy-fetal behaviours, normal and abnormal (when to seek help)** | *** moves on to the next topic asking the group, how many times they should they feel the baby moving, over 28 weeks. *** says yes, that’s right. That shows that the baby is healthy, receiving oxygen. | Group 3 |
| **Connects Women** | **Providing information for possible future healthcare interventions (outside MB)** | *** asks the group – so why would you want to give this? Give contractions? | Group 3 |
| **Connects Women** | **Participant interaction of listening to others and being respectful** | The other participants nodded, stayed silent. | Group 3 |
| **Connects Women** | **Coping with past (traumatic) birth-related experiences** | Observation. | Group 3 |
| **Cultural and Environmental Shocks** | **Unfamiliarity with HealthCare Services (where to go, when to go, common interventions)** | Observation. | Group 3 |
| **Feels like Home** | **Offering resources to reduce barriers (cost) of transporting to MB** | I have to be downtown by 3pm (230 at this time). *** says okay no problem. 33 asks how she can get a bus ticket. *** says oh yes, and goes up stairs to get her one . | Group 3 |
| **Busy-ness** | **Groups starting late as clients arrive late** | As we all went to the living room to begin the session, it was already 1:30. 31 turned to me and said that she had to leave. She seemed disappointed that the group had started late. | Group 3 |
| **Feels like Home** | **Feeling safe and willing to share difficult past experiences with other participants** | 32 shared that she had a C-section and lost her first baby girl. (She had a neutral facial expression when sharing this difficult information). | Group 3 |
| **Gives Hope** | **Facilitator easing anxieties about health risks by providing information** | ·         *** said that is called uterine prolapse, can be caused by the lifting, but “don’t worry, it is very rare”. The participants seemed relieved to hear that. | Group 3 |
| **Helps with All** | **Addressing participant questions, normalizing concerns** | ·         33 then said she is having some difficult controlling her bladder, when I cough or sneeze, a little pee comes out (she laughs). *** also smiling, says yes it is normal. | Group 3 |
| **Helps with All** | **Facilitator remaining neutral, judgement free, encouraging the clients to own their labour and delivery experience** | *** says that there is currently no evidence or research showing any long-term or permanent damage to the back from the epidural | Group 3 |
| **Helps with All** | **Sharing personal experiences (participant and facilitator), removes professional-only position, sense of relatedness** | 32 replied saying, yes in the later months of the pregnancy, she also said she felt disjointed after giving birth. | Group 3 |
| **Helps with All** | **Linking information with past experience (teaching strategy), increasing understanding for health interventions** | *** says; it is for opening the cervix, it is called “Augmentation of Labour”. There can also be “Induction of Labour” . It may be given if there is no progression in the labour, to accelerate it. | Group 3 |
| **Helps with All** | **Providing information about navigating healthcare services for labour and delivery** | objectives for today’s session, saying she would talk about the stages of labour and where and when to go to the hospital. | Group 3 |
| **Helps with All** | **Empowering the women by sharing knowledge** | her equipment (pelvic bone replica) and the baby doll to explain about the hormone Relaxin. “It relaxes and makes everything loose so the baby can pass through the pelvis”. | Group 3 |
| **Helps with All** | **Addressing participant questions, normalizing concerns** | *** then grabs her diagrams-poster boards and shows the uterus ligaments and their stretching. She explains that this occurs around 5 months. | Group 3 |
| **Helps with All** | **Prevention and Health Promotion for safe and healthy behaviours (post-partum)** | She said that after delivery, it is important to not be lifting anything too early after birth (again, 2 weeks vaginal, and 4 weeks C-section), nothing heavier than the baby. | Group 3 |
| **Helps with All** | **Teaching tools (visuals and equipment) to increase learning opportunity** | ·         *** pulls out the large poster boards and shows a uterus with a third trimester fetus, she points to where the bladder is and how it is being pressed down by the uterus. | Group 3 |
| **Helps with All** | **Reviewing information for accessing unfamiliar (or complex) healthcare systems** | hospital card ready. ·         *** jumps in and gives more information on the hospital card. She adds that there is a pre-registration for giving birth at the hospital.    32 continues, so yes, when you go with the card, you can go right to the birthing center. | Group 3 |
| **Helps with All** | **Correcting misunderstandings about the stages of labour, proper timing for when to access care** | 2 possibilities to go the hospital, 1; the water breaks (this happens about ¼ of the time – she continues to say that some Moms believe that it is only when the water breaks that they should go to the hospital. “No, No” she says, also for the contraction times. | Group 3 |
| **Helps with All** | **Managing expectations, providing insights into future experience with healthcare services and care** | *** then explains the normalities of being on the labour and delivery floor. Routine monitoring of the baby, if everything is okay, you are allowed to remove it. | Group 3 |
| **Helps with All** | **Providing information, rationale and expectations for a health intervention (epidural and its effects)** | Saying there is a difference from the C-section (spinal anaesthia) and Vaginal epidural. She grabs her pelvis to demonstrate where the needle goes, above into the back. First there is a local anaesthetic given, then 2^nd^ the large needle, the plastic tube remains and will be connected to another tube. When it functions well, it will reduce pain, but cannot move legs. | Group 3 |
| **Isolation** | **Loss of family (no support during pregnancy)** | give birth did not have any partner support during the labour, she then looked to a few participants and said to 36 and 37, your husbands are not here either right? They both nodded. 36 explained that her husband was in India and 37 said that her husband was in Cote-d’Ivoire. | Group 3 |
| **Isolation** | **Reduced Family Support (some do not have their Husbands in Canada)** | Observation. |  |
| **Know their Clients** | **MB personnel knowing patient, directing advice to specific situation** | *** looking at 33 during this statement. | Group 3 |
| **Know their Clients** | **Knowing clients, making information relevant, directed and specific** | ·         *** looks at 35, so for first-baby (35 – it was her first pregnancy), she says it should be 4 minutes for the contractions or if the water breaks. | Group 3 |
| **Modeling Behaviour** | **Providing referral to health-related products to address issue (pain)** | *** recommended using a pregnancy-belt to help with supporting the uterus, especially during the later months of pregnancy. She stood up and demonstrated how it is worn. | Group 3 |
| **Modeling Behaviour** | **Giving recommendation for daily physical activities, setting small realistic goals with clients** | And so with the back pain, it is better to move. So get in the habit of moving, even 15 minutes a day. I felt that *** was being more firm with her recommendation but also used an encouraging tone to try to motivate them to move, at least with some minimal goals that can be achieved (15 minutes a day). | Group 3 |
| **Modeling Behaviour** | **Sharing best-practices in post-partum care (muscle re-strengthening)** | ·         *** says yes, the Kegel Exercises, she then proceeds to explain the exercises, how many times to be effective, saying that repetition is important. | Group 3 |
| **Models Behaviour/Active Participation** | **Asking questions to participants, ensuring active participation and engagement** | ·         *** then asks the group “How can we make the muscles strong again? | Group 3 |
| **Models Behaviour/Active Participation** | **Empowering clients to be self-reliant on knowing best for their baby, cues to be aware of, steps to take** | you will know about it first. Using her diagram, she points to the large blood vessels on the right side, that bring blood and oxygen to the baby. If you don’t feel well, then yes, move positions, for instance you can feel dizzy or out of breath. | Group 3 |
| **Navigation of System** | **Bridging to other support services to navigate and learn healthcare systems** | ·         *** adds that you can also do the virtual visit, which is done once per month, in English and French, the dates are near the reception, to see the Labour and delivery floor beforehand, so you can orient yourself. | Group 3 |
| **Navigation of System** | **Bridging to other healthcare services, encouraging their use, removing barriers** | *** then adds that if you have an issue during your pregnancy to go to Pavillon K – 3rd floor, they will put the belts on and listen to the baby. | Group 3 |
| **Protection of Rights** | **Encouraging control of the client’s birthing experience (not to just be a passive recipient of care, to understand the care)** | *** explains that often there will be students as part of the care team, as it is a teaching hospital. She adds, to not be afraid to voice any concerns, if you don’t want an ‘open-door’ to your delivery experience. | Group 3 |
| **Protection of Rights** | **Empowering clients to their rights to receive respect and be treated with dignity when accessing healthcare (outside MB).** | *** concludes by encouraging the participants to raise any concerns that they may have about their birthing experience, you should be treated with respect, if there is any abusive or inappropriate behaviour, it is not acceptable, so please let us know, there are steps we can take. We take out work here seriously (at MB) and we hope to that are colleagues act the same, and treat you with respect and dignity. | Group 3 |
| **Protection of Rights** | **The potential for discrimination due to social status when accessing care (Visible Minority, Migrant-Statuses)** | Observation. | Group 3 |

| **Category** | **Code** | **Excerpt** | **Source** |
| --- | --- | --- | --- |
| **Busy-ness** | **Groups starting late as clients arrive late** | The group started, by the time everyone arrived, at 10:15 (planned for 9:30). | Group 4 |
| **Busy-ness** | **Allowing time** | 47 came in at this time late, | Group 4 |
| **Busy-ness** | **Allowing flexibility with participation** | 41 left early from the group (1050). | Group 4 |
| **Busy-ness?** | **Allowing flexibility in participation and timing** | 43 has put on her jacket, looks not very interested anymore, wanting to leave I think. | Group 4 |
| **Child Development Issues** | **Behavioural challenges with child** | He is walking away, distracted. | Group 4 |
| **Child Development Issues** | **Challenge: Child behaviour, requiring frequent interventions** | 44 -is standing hovering over her son, she is also talking photos of him while he plays. I feel that she feels she has to stay close to her son, because he is prone to hitting and needing interventions and frequent corrections. | Group 4 |
| **Child Development Issues** | **Getting to know other parents** | 43 also comes over to try and console the daughter. Asking the mom ‘whats wrong”. She replies saying; she wants to go home. | Group 4 |
| **Child Development Issues** | **Normalizing Behaviours** | Educator said, we see this misbehaviour in this type of environment, they is a lot of stimulation for them. 44 explains he is okay at home, F replies that is normal. | Group 4 |
| **Child Development Issues** | **Facilitator providing discipline** | 44 hitting again, he intervenes saying “ No, no! On frappe pas!” | Group 4 |
| **Child Development Issues** | **Parents also intervening to provide discipline** | 44 mom notices and intervenes as well, pulling him off the blocks. | Group 4 |
| **Child Development Issues** | **Parents providing discipline** | 44 is having some trouble with her son, she closes his food box, he gets up from his chair and starts to whine. | Group 4 |
| **Children as Purpose** | **Children as motivation, reason** | and watching all of the kids playng. | Group 4 |
| **Children as Purpose** | **Children as reason, happiness from parenting** | 42,44, and 46 are all standing up watching the kids | Group 4 |
| **Connects Women** | **Building relationships within similar ethnic communities** | 43 and 44 are sitting next to each other, speaking in their first language together in between the songs. (urdu?) They are gesturing towards their children, seems like they are asking questions about each other’s children. Asking about their age. | Group 4 |
| **Connects Women** | **Relationships within ethnic community, comfort** | talking in their first-language, look at ease, relaxed while interacting. | Group 4 |
| **Connects Women** | **Relationships within ethnic community, comfort** | 44 and 46, are chit chatting during the free play times, in their mother tongue. | Group 4 |
| **Connects Women** | **Relationships between participants** | 42 and 46 still side by side. Talking at times, laughing and smiling. | Group 4 |
| **Connects Women** | **Warm, humor** | All of the moms laugh at what he says. | Group 4 |
| **Feels like Home** | **Comfort** | between their legs, sit on the mats. | Group 4 |
| **Feels like Home** | **Children’s comfort with each other during group, familiarity? Comfort?** | All of the kids are sitting at the small tables during snack time. | Group 4 |
| **Feels like Home** | **Creating a calm and relaxing environment** | setting a calmer atmosphere after all the higher-pace activities. | Group 4 |
| **Gives Hope** | **Appreciation of referrals and support** | 44 looked appreciative of these comments. | Group 4 |
| **Gives Hope** | **Positive reinforcement for speaking French** | oh tu parle francais?! (Loud and excited). He high-fives him. “Bravo! Champion!” | Group 4 |
| **Helps with All** | **Facilitor addressing behavioural cocnerns** | Educator approaches 45, says’ she is crying a lot?” is this normal? She replies saying no. | Group 4 |
| **Helps with All** | **Providing practical resources to parents** | Educator brings a book to 47, have you seen this? It is a book for helping Moms to explain the pregnancy to young ones . | Group 4 |
| **Language Barrier?** | **Not following directives** | 42 is nodding throughout the instruction but does not remove her daughter’s sweater. | Group 4 |
| **Maintaining Traditions and Language** | **Maintenance of mother-tongue, first language with children** | . During the snack time, 46 is speaking in her mother tongue to her child. | Group 4 |
| **Modeling Behaviour** | **Encouraging personal hygiene practices** | now it is time for snack time. Lets go wash our hands. | Group 4 |
| **Modeling Behaviour** | **Facilitator demonstration of child discipline, used by parent following** | This has been previously demonstrated by the Educator in the group I attended before, that the child should be sitting when eating). | Group 4 |
| **Modeling Behaviour** | **Offering suggestions** | Educator shared that she may not be feeling well, then it might be better to go home, if it is more behaviour based, (due to many people), it is better that she stays here and gets used to it . | Group 4 |
| **Modeling Behaviour** | **Offering recommendations, re adapting to cold weather** | remove her daughter’s coat while inside, or else she will become cold when she goes outside (due to perspiration). | Group 4 |
| **Modeling Behaviour** | **Providing practical advice** | “I am sweating, she must be” Pointing to her daughter. The mom laughs, but still does not remove the sweater. | Group 4 |
| **Modeling Behaviour** | **Facilitator providing discipline** | Educator and he quickly disciplines him. “No, No!” “On frappe pas!”.  ( | Group 4 |
| **Modeling Behaviour** | **Parents observing and agreeing with discipline provided** | 44 mother watches, looks on agreeingly. | Group 4 |
| **Modeling Behaviour** | **Facilitator demonstrating being gentle** | “This is how you touch Mommy” – as he pats her shoulder gently | Group 4 |
| **Modeling Behaviour** | **Providing discipline to children** | educator intervenes saying; No, No! On pousse pas!” | Group 4 |
| **Modeling Behaviour** | **Receptive to input and advice** | 44 is listening to him, nodding, listening attentively. | Group 4 |
| **Models Behaviour/Active Participation** | **Facilitator asking questions, re child behaviour** | Educator turns to 45 mother, what is happening? The daughter is crying quite loudly. | Group 4 |
| **Models Behaviour/Active Participation** | **Facilitator asking questions about child behaviours, addressing concerns** | what do you mean when you said she doesn’t eat well?” | Group 4 |
| **Models Behaviour/Active Participation** | **Facilitor encouraging parent participation** | Come with her, she wants to try new things, and you have to come help” She agrees, nodding, and gets up and goes with her daughter to the toy tent. | Group 4 |
| **Models Behaviour/Active Participation** | **Encouraging parental involvement** | F turns to her – we have 1 more activity, will you stay for 1 more? She agrees. | Group 4 |
| **Models Behaviour/Active Participation** | **Mother participation with children** | Everyone is singing along, clapping their hands, grabbing their child’s hands and clapping with them. | Group 4 |
| **Navigation of System** | **Offering referral support to parents (daycare)** | ”. He explains that it may be hard for their first part or few months, but when finding a day care, | Group 4 |
| **Navigation of System** | **Facilitating referral to external resource** | tell them you are being followed by MB, and I can talk to them;  so | Group 4 |
| **Pregnancy Stresses** | **Allowing for parents to rest** | 47 is sitting at the back of the room, I feel that she is tired and wants to rest given her pregnancy and later gestational age. She starts reading the book, also while watching her two kids play. | Group 4 |
| **Varied Team** | **Facilitator animation** | Educator is really animated during the song, | Group 4 |

**General notes**

| **Category** | **Excerpt** | **Source** | **Date (September-December 2017)** |
| --- | --- | --- | --- |
| **Active Participation** | She went around the table and asked who planned to breastfeed and also, who had some difficulties breastfeeding with their last baby. | **Pre-Natal Group PEX** | October 18th |
| **Active Participation** | Throughout the teaching, she ensured it was more participative, by asking questions before simply giving answers. | **Pre-Natal Group PEX** | October 18th |
| **Agency** | Some mothers seemed more interested than others. Some were actively listening (eye contact, nodding, asking questions), | **Pre-Natal Group PEX** | October 18th |
| **Agency** | The parents were “very interested to learn about the topic”. | **Team Mtg - PEX** | October 10th |
| **Agency** | She was from New Guinea and is currently learning French. | **PEX** | October 5th |
| **Agency** | However, she wanted to keep the baby . | **CDN Team Meeting** | September 19th |
| **Appreciative of Freedom** | However, the father and mother are both trying to teach their children equality here, saying that all members of the family are equal because they are now in Canada. The | **PEX Team Meeting** | September 26th |
| **Appreciative of Freedom?** | She comes from Pakistan. S.W involved. She was not aware that abortion was legal in Canada, when she was told about this option. | **PEX Team Meeting** | September 26th |
| **Busy-ness** | as the parents and siblings are busy. | **Team Mtg - PEX** | October 17th |
| **Busy-ness** | One Mother had to leave early to pick up her children from daycare? School ? | **CDN Post-Natal Group** | September 7th |
| **Child Development Delays** | Her child is showing signs of TSR (autism). | **CDN Team Meeting** | October 3rd |
| **Child Development Issues** | 2 years old would benefit from daycare to receive more social interactions as his stimulation at home is limited | **Team Mtg - PEX** | October 17th |
| **Child Development Issues** | Child crying a lot in the group sessions, yet it is improving and going better. | **Team Mtg - PEX** | October 10th |
| **Child Development Issues** | Educator (said difficult case), the child has development delays. | **Team Mtg - PEX** | October 10th |
| **Child Development Issues** | . The parents are concerned as well as their eldest child is showing signs of having a lazy eye | **Team Mtg - PEX** | October 10th |
| **Connects (Men)** | Des groupes pour des papas”. | **Team Mtg - PEX** | October 10th |
| **Connects Women** | There were 11 mothers. | **Pre-Natal Group PEX** | October 18th |
| **Connects Women** | table were the mothers introduced themselves, where they were from, how many children they had and their estimated due date | **Pre-Natal Group PEX** | October 18th |
| **Connects Women** | Most of the women attended the group regularly , | **Pre-Natal Group PEX** | October 18th |
| **Connects Women** | Some mothers also brought their other children. 3 mothers had children, one was 8 months old and stayed with her the entire time. Another had a 3-year-old, who played just outside the meeting room (we left the door open to maintain watch of him). Another mother had a 2-year-old. | **Pre-Natal Group PEX** | October 18th |
| **Connects Women** | There was a total of 10 mothers who attended the session . | **CDN Post-Natal Group** | September 28th |
| **Connects Women** | What was also neat was one mother translated  for | **CDN Post-Natal Group** | September 28th |
| **Connects Women** | The nurse at CDN said that there were some mothers who regularly attended the group sessions on Thursdays | **CDN Post-Natal Group** | September 21st |
| **Connects Women** | There were a total of 10 mothers | **CDN Post-Natal Group** | September 21st |
| **Connects Women** | At last week’s fete d’automne, there were 38 mothers who are present, saying it was a successful event | **CDN Team Meeting** | September 19th |
| **Connects Women** | There were 4 Mothers Present. 3 with infants. | **CDN Post-Natal Group** | September 7th |
| **Family Seperation** | She is from Senegal, and she wishes for her mother to emigrate from there. | **CDN Team Meeting** | September 19th |
| **Finances** | The couple has 4 children, difficult financial situation. He works, has a car. The team is wondering how there is financial difficulties (send money back home?). | **CDN Team Meeting** | October 3rd |
| **Finances** | as he did not want the child due to her not being covered by health insurance and the costs would be too high. | **CDN Team Meeting** | September 19th |
| **Finances** | he also pressured her to have an abortion because again, she was not covered by medicare . | **CDN Team Meeting** | September 19th |
| **Finances** | She also recently lost her housing, and what was inside the apartment. | **CDN Team Meeting** | September 12th |
| **Friends** | She has some friends in the building that she lives in. | **CDN Team Meeting** | September 19th |
| **Gives Hope** | . Mother strength discussed was she is very stimulant/active with her babies . | **CDN Team Meeting** | September 12th |
| **Help and Support** | She mentioned to the team that the “CLSC est correcte” for her pre-natal following . | **Team Mtg - PEX** | October 17th |
| **Help and Support** | She now receives $1 per day for daycare for each child | **PEX Team Meeting** | September 26th |
| **Helps with All** | The midwife talked about the size of the baby’s stomach (a cherry) and the first milk that is there after birth (colostrum, of only a few drops). She explained the feedback loop for breastmilk – infant stimulation to keep replacing the milk. She did this through drawing on the whiteboard. | **Pre-Natal Group PEX** | October 18th |
| **Helps with All** | She then asked the group what the hunger signals were. 1 mother said crying. The midwife shared what they were (bringing hands to mouth, moving around etc), and said crying was a late sign. She said it is important to notice the signs because it is more difficult for the baby to latch when they are crying. | **Pre-Natal Group PEX** | October 18th |
| **Helps with All** | The team also spoke about “Operation Pere-Noel”. This is a Montreal-initiative where volunteers purchase presents for children for Christmas. MB can recommend their clients who they feel are most in need to receive gifts for their children at the holidays. | **Team Mtg - PEX** | October 17th |
| **Helps with All** | Family in need of household items. MB to donate a fridge to the family. | **Team Mtg - PEX** | October 10th |
| **Helps with All** | SW suggested starting with the Immigration process to achieve a baseline with the clients. | **Team Mtg - PEX** | October 10th |
| **Helps with All** | We began by going to a nearby market to get some vegetables. Then, we pickd up her 4-year old son from school. Back at MB, we demonstrated (with her doing as much as possible as well) how to make purees for her 8 month year old . | **PEX** | October 5th |
| **Helps with All** | the SW spoke about the Holidays, including the ability for the mothers to receive one gift for child and non-perishable food items, if desired. | **CDN Post-Natal Group** | September 28th |
| **Helps with All** | *** is to do a cooking and kitchen session with her for healthy eating. | **PEX Team Meeting** | September 26th |
| **Helps with All** | it was an information session on dental care and hygiene for the mothers and their children. | **CDN Post-Natal Group** | September 21st |
| **Helps with All** | Follow-up plan is to make the request for her mother to immigrate to Canada. | **CDN Team Meeting** | September 19th |
| **Helps with All** | Plan from Social Work 🡪 Home visit. Make a financial aid demand for the mother. | **CDN Team Meeting** | September 12th |
| **Helps with All** | PLAN: SW to make the request for financial aid for their third child and to follow-up regarding their immigrations status request and decisions. | **CDN Team Meeting** | September 12th |
| **Helps with All** | The MB team also gave her formula, as she was having some difficulties and anxieties that her baby was not feeding enough. | **PEX Team Meeting** | September 26th |
| **Helps with All** | The team will also follow-up to see if we can collect some clothing, creams and also if there is a double-stroller available for her. | **CDN Team Meeting** | September 19th |
| **Immigration Process** | The team spoke about prioritizing refugees and refugee claimants and families that are low-income . | **Team Mtg - PEX** | October 17th |
| **Immigration Process** | No RAMQ. | **CDN Team Meeting** | October 3rd |
| **Immigration Process** | explain in her application for status, how she is implicated into Canadian society, submit the birth declaration to the government and find an immigration lawyer. | **CDN Team Meeting** | October 3rd |
| **Immigration Process** | As this client has no insurance, the cost the family owed to the hospital was $ 1,200 for the delivery and infant care (Baby was premature).  The | **PEX Team Meeting** | September 26th |
| **Immigration Process** | She has found an apartment, however she needs a co-signer because of her immigrations status and because she is social aid. | **CDN Team Meeting** | September 19th |
| **Immigration Process** | She was refused status. | **CDN Team Meeting** | September 12th |
| **Isolation** | This mother is alone at the home. | **Team Mtg - PEX** | October 10th |
| **Isolation** | From the Philippines, 40 years old. Single-parent. | **CDN Team Meeting** | October 3rd |
| **Isolation** | She is also recently separated from her husband, who is back in Mauritania. | **CDN Team Meeting** | October 3rd |
| **Isolation** | Because she is keeping the baby, her partner has left her. | **PEX Team Meeting** | September 26th |
| **Isolation** | – From Burkina Faso. She is a comedian, specializes in puppetry. She has come to Canada alone, currently pregnant (14-weeks). | **PEX Team Meeting** | September 26th |
| **Isolation** | Came on September 3^rd^. 5 children. No partner. | **CDN Team Meeting** | September 19th |
| **Isolation** | The father of the baby is very critical and ended up not being helpful and left.  The | **CDN Team Meeting** | September 19th |
| **Isolation** | She has two daughters. She is a single mother – mono-parental. | **CDN Team Meeting** | September 12th |
| **Isolation** | Woman from Niger. Her partner does not want to be involved. | **CDN Team Meeting** | September 12th |
| **Isolation** | First pregnancy. 19 years old. From Mauritania. Single-parent. | **CDN Team Meeting** | September 12th |
| **Knows Clients** | The SW jumped in and said that being reserved was a cultural phenomenon from Eritrea. | **CDN Team Meeting** | October 3rd |
| **Knows Clients** | Client is not ready to hear this at all, rather the team has been approaching it by speaking of development delays instead. | **CDN Team Meeting** | October 3rd |
| **Knows their Clients** | . The midwife shared that she did a home-visit to see more of the couple dynamics . | **PEX Team Meeting** | September 26th |
| **Language Barriers** | There remains some language barriers as she does not always understand or able to reply to questions. | **PEX** | October 5th |
| **Language Barriers** | From Eritrea. 26 years old, has been in Canada for one year. Speaks a bit of Arabic, she has a private interpreter (friend?). | **CDN Team Meeting** | October 3rd |
| **Language Barriers** | The midwife said that it was difficult to get information from her as she did not speak English or French , | **CDN Team Meeting** | October 3rd |
| **Language Barriers** | for two women in Arabic, as they did not understand both English or French | **CDN Post-Natal Group** | September 28th |
| **Language Barriers** | The wife does understand French, yet the husband does the translations, requiring his presence for her appointments. | **PEX Team Meeting** | September 26th |
| **Living in Underprivileged Areas?** | She is currently renting one-bedroom in a shared apartment. The SW said she will have to move after the baby comes as the place is too small. | **PEX Team Meeting** | September 26th |
| **Living in Underprivledged Areas/Finances** | One case; 37 weeks pregnant, lived in Montreal-Nord, the reason to be a LMB client is her unstable housing situation. | **CDN Team Meeting** | September 5th |
| **LMB Challenge** | The SW also explained some of the budgetary concerns that MB has. For instance, the organization is coming under stricter supervision by the Government . | **CDN Post-Natal Group** | September 28th |
| **LMB Limiation** | There was discussion of transferring these phone calls to the nurse, but it would be too time consuming. | **Doublons Clinique** | October 5th |
| **LMB Limitation** | For him, he said it is important to follow the children from 0-5. | **Team Mtg - PEX** | October 17th |
| **LMB Limitation** | Speaking about the tool led into the discussion (which has come up quick often), of the difficulties in balancing the MB philosophy and practicing holistic and big picture care with families versus the limited resources and time that the organization has. | **Team Mtg - PEX** | October 10th |
| **LMB Limitation** | The problem that is facing the organization is that it has been 10 years since their founding and there are now many or even too many clients, which will continue to grow.  This | **Doublons Clinique** | October 5th |
| **LMB Limitation** | The philosophy of MB is to follow the entire family, but the doctors were recommending maybe only taking the mothers in the future.  In | **Doublons Clinique** | October 5th |
| **LMB Limitation** | In addition, it was said there is a conflict or discrepancy between the capacity of MB and its mission . | **Doublons Clinique** | October 5th |
| **LMB Limitation** | The team expressed concerns that they are quite full with clients at the moment but may have to turn down new clients that are very vulnerable, referring them to the CLSC. | **PEX Team Meeting** | September 26th |
| **LMB Limitation, Time** | The M.D again expressed concerns for making appointments only with the husbands | **Team Mtg - PEX** | October 17th |
| **LMB Limitation-Challenge** | . This seems to be under the circumstance of high client loads and demands on the team . | **Team Mtg - PEX** | October 17th |
| **LMB Limitation-Challenge** | MD wants to transfer the husband file to the CLSC, she said that he takes too much time up during the appointments for himself, | **Team Mtg - PEX** | October 10th |
| **Love and Support** | Refugee from Haiti from CLSC CDN. Partner with her. G2P1. | **CDN Team Meeting** | September 19th |
| **Love and Support** | Her husband said he is willing to take the role of the father with the children, even if he is not the biological father. | **CDN Team Meeting** | September 12th |
| **Modeling Behaviour** | She also brought out her cotton breast to also demonstrate where the milk comes from (the areola, not the nipple – which can lead to pain during breastfeeding). | **Pre-Natal Group PEX** | October 18th |
| **Models Behaviour** | He gave out books to the parents and did some demonstrations on how to discipline effectively. | **Team Mtg - PEX** | October 10th |
| **Navigating System** | Psychoeducator confirmed that the foster home is a go. The family is located in Saint-Lazare. It will be a 30 day placement. She has a meeting with Batshaw (Youth and Family Centers). | **Team Mtg - PEX** | October 17th |
| **Navigating System** | The CLSC representative mentioned that this will reduce the waiting times that families currently wait for receiving services. This hour took up the rest of the meeting. As it related mostly to the educator, he asked a lot of questions about the procedure for referrals. | **Team Mtg - PEX** | October 17th |
| **Navigating System** | The dental students demonstrated proper brushing and flossing techniques, spoke about cavities, baby teeth, teething and dental coverage in Quebec (up to 10 years old, free for the exams, not the cleaning costs). | **CDN Post-Natal Group** | September 21st |
| **Navigation of System** | Need to be linked to another midwife for delivery. | **Team Mtg - PEX** | October 10th |
| **Navigation of System** | Yet requires proper links to be made for family before closing and transferring the file. | **Team Mtg - PEX** | October 10th |
| **Navigation of System** | The CLSC teams in the quartier are not really aware of MB services. It has been 2-3 years since MB has presented the organization to the CLSC | **Team Mtg - PEX** | October 10th |
| **Navigation of System** | The careplan must be discussed with the new family doctors before transferring the files. | **Doublons Clinique** | October 5th |
| **Navigation of System** | The main objective of the group today was to share information on Femme de St-Laurent, where a representative came to speak about their services for which the SW said are nicely complementary to MB . | **CDN Post-Natal Group** | September 28th |
| **Navigation of System** | The SW should explain what other services are available to the family if need be. | **CDN Team Meeting** | September 12th |
| **Parenting Stresses** | She does all of the work at the house. Has a new baby and is very tired . | **CDN Team Meeting** | October 3rd |
| **Parenting Stresses** | This woman visited her at the home, where she was crying a few times due to her situation. She has two babies (twins), 2 weeks post-partum. | **CDN Team Meeting** | September 19th |
| **Pregnancy & Parenting Stresses** | Mental health history. Having difficulties in the post-partum period. | **CDN Team Meeting** | October 3rd |
| **Pregnancy Stresses** | She recently gave birth to a large baby through Cesarean. History of High Blood pressure (Preclampsia) during the pregnancy. | **CDN Team Meeting** | September 19th |
| **Protection of Rights** | Also, there has been some issues where MB clients walk-in to the CLSC and are told to come to MB. Yet, as they live in the quartier, they have the right to the walk-in services at the CLSC. | **Doublons Clinique** | October 5th |
| **Protection of Rights** | She is a mother ‘sans papier’. | **PEX Team Meeting** | September 26th |
| **Protection of Rights** | MB has limits for how many clients they can take without RAMQ, as the organization is financed by the public system. | **CDN Team Meeting** | September 12th |
| **Protection of Rights** | During the meeting, the team is diligent with keeping the doors closed etc when discussing patient cases. + Confidentiality. | **CDN Team Meeting** | September 12th |
| **Culture and Environmental Shocks** | Due in March 2018, also has a 9 year old child. From the Philippines, came to Canada in 2015. Troubles with adaptation | **CDN Team Meeting** | October 3rd |
| **Immgration Process** | She said she wants him to help cover for the medical costs. About $5000 of fees. | **CDN Team Meeting** | September 19th |
